# Validation of a Bioinformatics Workflow for Routine Analysis of Whole-Genome Sequencing Data and Related Challenges for Pathogen Typing in a European National Reference Center: *Neisseria meningitidis* as a Proof-of-Concept

**DOI:** 10.3389/fmicb.2019.00362

**Published:** 2019-03-06

**Authors:** Bert Bogaerts, Raf Winand, Qiang Fu, Julien Van Braekel, Pieter-Jan Ceyssens, Wesley Mattheus, Sophie Bertrand, Sigrid C. J. De Keersmaecker, Nancy H. C. Roosens, Kevin Vanneste

**Affiliations:** ^1^Transversal Activities in Applied Genomics, Sciensano, Brussels, Belgium; ^2^Bacterial Diseases, Sciensano, Brussels, Belgium

**Keywords:** *Neisseria meningitidis*, whole-genome sequencing, validation, public health, national reference center

## Abstract

Despite being a well-established research method, the use of whole-genome sequencing (WGS) for routine molecular typing and pathogen characterization remains a substantial challenge due to the required bioinformatics resources and/or expertise. Moreover, many national reference laboratories and centers, as well as other laboratories working under a quality system, require extensive validation to demonstrate that employed methods are “fit-for-purpose” and provide high-quality results. A harmonized framework with guidelines for the validation of WGS workflows does currently, however, not exist yet, despite several recent case studies highlighting the urgent need thereof. We present a validation strategy focusing specifically on the exhaustive characterization of the bioinformatics analysis of a WGS workflow designed to replace conventionally employed molecular typing methods for microbial isolates in a representative small-scale laboratory, using the pathogen *Neisseria meningitidis* as a proof-of-concept. We adapted several classically employed performance metrics specifically toward three different bioinformatics assays: resistance gene characterization (based on the ARG-ANNOT, ResFinder, CARD, and NDARO databases), several commonly employed typing schemas (including, among others, core genome multilocus sequence typing), and serogroup determination. We analyzed a core validation dataset of 67 well-characterized samples typed by means of classical genotypic and/or phenotypic methods that were sequenced in-house, allowing to evaluate repeatability, reproducibility, accuracy, precision, sensitivity, and specificity of the different bioinformatics assays. We also analyzed an extended validation dataset composed of publicly available WGS data for 64 samples by comparing results of the different bioinformatics assays against results obtained from commonly used bioinformatics tools. We demonstrate high performance, with values for all performance metrics >87%, >97%, and >90% for the resistance gene characterization, sequence typing, and serogroup determination assays, respectively, for both validation datasets. Our WGS workflow has been made publicly available as a “push-button” pipeline for Illumina data at https://galaxy.sciensano.be to showcase its implementation for non-profit and/or academic usage. Our validation strategy can be adapted to other WGS workflows for other pathogens of interest and demonstrates the added value and feasibility of employing WGS with the aim of being integrated into routine use in an applied public health setting.

## Introduction

Whole-genome sequencing (WGS) has become a well-established technique, spurred by the rapid development of different next-generation sequencing (NGS) technologies, and ample case studies have been published in recent years that demonstrate the added value of WGS for surveillance monitoring and outbreak cases for many microbial pathogens of interest in public health (Mellmann et al., 2011; Kwong et al., 2015; Aanensen et al., 2016; Charpentier et al., 2017; Harris et al., 2018). WGS offers the potential to replace traditional molecular approaches for typing of microbial pathogens because of several advantages: more cost-efficient, less labor-intensive, faster, more information per sample and at a higher resolution (Gilmour et al., 2013; Kwong et al., 2015; Allard, 2016; Deurenberg et al., 2017). WGS for instance enabled the development of novel typing methods such as core genome multilocus sequence typing (cgMLST), which expands the breadth of standard MLST by including several hundreds of loci (Maiden et al., 2013). Additionally, the resolution up to the nucleotide level enables pathogen comparison and clustering with unprecedented precision (Carriço et al., 2013; Dallman et al., 2015; Ronholm et al., 2016). A gap nevertheless still exists between the acclaimed success and the everyday implementation and usage of this technology in a public health setting, especially for many national reference laboratories (NRLs) and centers (NRCs) in smaller and/or less developed countries, which do not always have access to the same resources that are available for public health agencies in larger and/or more developed countries that already routinely process large volumes of samples with NGS technologies (WHO, 2018). In Europe, recent surveys in 2016 by both the European Food Safety Authority (EFSA) (García Fierro et al., 2018) and the European Centre for Disease Prevention and Control (ECDC) (Revez et al., 2017) indicated that NGS was being used in 17 out of 30 and 25 out 29 responding constituents, respectively, and that large discrepancies existed between different European countries in the advancement of implementing this technology for different microbial pathogens of interest, for which the lack of expertise and financial resources were often quoted.

The data analysis bottleneck in particular represents a serious obstacle because it typically consists out of a stepwise process that is complex and cumbersome for non-experts. An overview of data analysis tools that can be used for capacity building was recently published by the ENGAGE consortium, which aims to establish the NGS ability for genomic analysis in Europe (Hendriksen et al., 2018). Many of these tools still require substantial expertise because they are only available using the command line on Linux, but a subset is also available as web-based platforms with a user-friendly interface open to the scientific community. For instance, an entire suite of tools for pathogen characterization through WGS data has been made available by the Center for Genomic Epidemiology^1^ hosted at the Technical University of Denmark allowing, among others, assembly, serotyping, virulence detection, plasmid replicon detection, MLST, and phylogenetic clustering (Deng et al., 2016), and is frequently used by the different enforcement laboratories in Europe (García Fierro et al., 2018). PubMLST^2^ is another popular web-based platform that maintains databases with sequence typing information and schemas for a wide variety of pathogens, and can be queried with WGS data (Jolley and Maiden, 2010). Some resources have also been developed tailored specifically toward certain pathogens, such as NeisseriaBase as a platform for comparative genomics of *Neisseria meningitidis* (Katz et al., 2011). While these resources are most definitely useful, they do have some disadvantages. Several databases and tools typically still need to be combined manually, whereas an integrated approach encompassing all required analytical steps is preferred for a public health laboratory (Lindsey et al., 2016). In addition, a set of standardized tools and guidelines is not defined yet, limiting the collaboration and reproducibility between different NRCs and NRLs that all have their own way of analyzing WGS data (Rossen et al., 2018). Many of these resources are also lacking traceability. Database versions, and tool parameters and versions, can be missing in the output or change without notice, making it hard to compare and exchange results with other laboratories. Systematic international collaboration between different NRCs and NRLs across multiple years is, however, only possible when a standardized workflow is used. The time between submitting data to the webserver and receiving results varies and could be a limiting factor in emergency cases, unless the application is locally installed.

Moreover, most NRCs and NRLs operate according to a strict quality system that requires an extensive validation to demonstrate that methods are “fit-for-purpose,” thereby fulfilling the task for which the method was developed in order to produce high-quality results, which is also important to obtain accreditation (Rossen et al., 2018). For classical typing methods, the process of validation is typically dependent upon the exact type of analysis and the (often limited) number of well-characterized samples. A standardized approach to validate WGS for routine use in public health laboratories for microbiological applications is not available yet and still under development by the International Organization for Standardization (ISO) in the working group “WGS for typing and genomic characterization” (ISO TC34-SC9-WG25) (Portmann et al., 2018). Although this working group is expected to lead to an official standard in the next few years, many NRCs and NRLs already face the need for validation at the current moment, as evidenced by many recent case studies that describe the validation of components of the WGS workflow. Portmann et al. (2018) presented the validation of an end-to-end WGS workflow for source tracking of *Listeria monocytogenes* and *Salmonella enterica*. Holmes et al. (2018) reported the validation of a WGS workflow for the identification and characterization of Shiga toxin-producing *Escherichia coli* (STEC) focusing on standardization between different public health agencies. Mellmann et al. (2017) described external quality assessment options for WGS. Lindsey et al. (2016) documented the validation of WGS for a commercial solution for STEC. Dallman et al. (2015) reported the validation of WGS for outbreak detection and clustering of STEC. Recently, Kozyreva et al. (2017) detailed an entire modular template for the validation of the WGS process not limited to certain species but generally applicable for a public health microbiology laboratory. Such case studies help to propel the implementation of WGS for clinical microbiology, but the comprehensive validation of the underlying bioinformatics analysis has not been documented yet. This is however of paramount importance as bioinformatics analysis is inherently part of the evaluation of every step of the entire WGS workflow going from sample isolation, DNA extraction, library preparation, sequencing, to the actual bioinformatics assays. It is therefore imperative to thoroughly validate this step before the other levels of the WGS workflow are evaluated (Angers-Loustau et al., 2018). The bioinformatics analysis acts as the “most common denominator” between these different steps, allowing to compare and evaluate their performance. An exhaustive validation of the bioinformatics analysis for WGS for clinical and/or public health microbiology has, however, not yet been described, and is not an easy task because classical performance metrics cannot be directly applied to bioinformatics analyses, and it is often not possible to obtain a realistic ‘gold standard’ for systematic evaluation (Kozyreva et al., 2017).

As a proof-of-concept, we focus here on *N. meningitidis*, a Gram-negative bacterium responsible for invasive meningococcal disease, causing symptoms such as meningitis, septicemia, pneumonia, septic arthritis, and occasionally inflammatory heart disorders. The Belgian NRC *Neisseria* analyses approximately 100–130 strains per year, and traditionally employed the following molecular techniques for pathogen surveillance: species identification by real-time polymerase chain reaction (qPCR); matrix assisted laser desorption/ionization; or biochemistry to verify that the infection is *N. meningitidis* and not another pathogen also causing bacterial meningitis such as *Streptococcus pneumonia* or *L. monocytogenes*; serogroup determination by slide agglutination or qPCR of the capsule genes; drug susceptibility and antibiotics resistance testing by determining the minimum inhibitory concentration on plated samples; and subtyping by PCR followed by Sanger sequencing of several loci of interest such as for instance the classic seven MLST genes (Maiden et al., 1998) and vaccine candidates such as Factor H-binding protein (*fHbp*) (Brehony et al., 2009). The rapid progression, high fatality rate, and frequent complications render *N. meningitidis* an important public health priority in Belgium and an ideal candidate to investigate the feasibility of using WGS while effectively mitigating the data analysis bottleneck. At the same time, the strict requirement for ISO15189 accreditation, which deals with the quality and competence of medical laboratories for all employed tests and is demanded by the Belgian national stakeholder (the national institute for health and disability insurance), renders it an ideal proof-of-concept to investigate how to validate the bioinformatics workflow.

We describe here the first exhaustive validation of a bioinformatics workflow for microbiological isolate WGS data, extensively documenting the performance of different bioinformatics assays at the genotypic level by means of a set of traditional performance metrics with corresponding definitions and formulas that were adapted specifically for WGS data. The WGS workflow was evaluated both on a set of sequenced reference samples and collected public data generated by means of the Illumina sequencing platforms, and demonstrates high performance. Our validation strategy can serve as a basis to validate other bioinformatics workflows that employ WGS data, irrespective of their targeted pathogen, and illustrates the feasibility of employing WGS as an alternative to traditional molecular techniques for a relatively small-scale laboratory in a public health context.

## Materials and Methods

### Bioinformatics Workflow

#### Data (Pre-)processing and Quality Control

**FIGURE 1:**
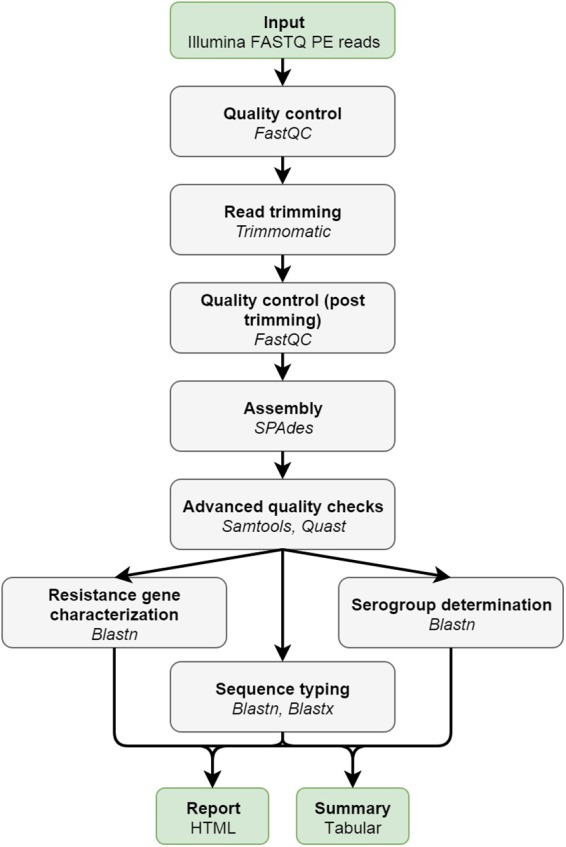
Overview of the bioinformatics workflow. Each box represents a component corresponding to a series of tasks that provide a certain well-defined functionality (indicated in bold). Major bioinformatics utilities employed in each module are also mentioned (indicated in italics). Abbreviations: paired-end (PE).

Figure 1 provides an overview of the bioinformatics workflow. The workflow supports all WGS data generated by means of the Illumina technology. Pre-trimming data quality reports are first generated to obtain an overview of raw read quality with FastQC 0.11.5 (available at https://www.bioinformatics.babraham.ac.uk/projects/fastqc/) using default settings. Afterward, Trimmomatic 0.36 (Bolger et al., 2014) is used to trim raw reads using the following settings: “LEADING:10” (i.e., first remove all residues at the beginning of reads with a *Q*-score < 10), “TRAILING:10” (i.e., then remove all residues at the ending of reads with a *Q*-score < 10), “SLIDINGWINDOW:4:20” (i.e., then clip reads as soon as the average *Q*-score is <20 over a sliding window of four residues), and “MINLEN:40” (i.e., then remove all reads that are <40 residues after the previous steps). The “ILLUMINACLIP” option can be set to either “Nextera,” “TruSeq2,” or “TruSeq3” dependent upon the used sequencing protocol (see below), and is added to the command as “[adapter]-PE:2:30:10.” All other options are left at their default values. Post-trimming data quality reports are then generated using FastQC to obtain an overview of processed read quality. Afterward, processed paired-end reads are *de novo* assembled using SPAdes 3.10.0 (Bankevich et al., 2012). Orphaned reads resulting from trimming (i.e., reads where only one read of the pair survived) are provided to the assembler as unpaired reads. The “–careful” option is used. All other options are left at their default values (kmers are chosen automatically based on the maximum read length). Assembly statistics such as N50 and number of contigs are calculated with QUAST 4.4 (Gurevich et al., 2013) using default settings. The processed reads are then mapped back onto the assembled contigs using Bowtie2 2.3.0 (Langmead and Salzberg, 2012) with the following settings: “–sensitive,” “–end-to-end,” and “–phred33” (all other options are left at their default values). The mapped reads are used to estimate the coverage by calculating the median of the per position depth values reported by SAMtools depth 1.3.1 (Li et al., 2009) using default settings (SPAdes by default also maps reads back to the assembly but reports coverage in terms of kmer coverage). Lastly, several quality metrics are checked to determine whether data quality are sufficient before proceedings toward the actual bioinformatics assays. Threshold values for these quality metrics were set based on the quality ranges observed during validation by selecting more and less stringent values for metrics exhibiting less and more variation between samples/runs, respectively. An overview of all quality metrics and their corresponding warning and failure thresholds is provided in Table 1.

**Table 1:** Advanced quality control metrics with their associated definitions and threshold values for warnings and failures.

		Warning	Failure
Metric	Definition	threshold	threshold
Median coverage	Median coverage based on mapping of the trimmed reads against the assembly (Desai et al., 2013)	20	10
% reads mapping back to assembly	Percentage of the trimmed reads mapping back to the assembly (Smith-Unna et al., 2016)	95	90
% cgMLST genes identified	Percentage of cgMLST genes identified. Only perfect hits (i.e., full length and 100% identity) are considered (Mellmann et al., 2017)	95	90
Average read quality (*Q*-score)	*Q*-score of the trimmed reads averaged over all reads and positions	30	25
GC-content deviation	Deviation of the average GC content of the trimmed reads from the expected value for *N. meningitidis* (51.5%; Tettelin et al., 2000)	2	4
N-fraction	Average N-fraction per read position of the trimmed reads	0.05	0.10
Mean *Q*-score drop	Average position in the trimmed reads where the average *Q*-score drops below 28, expressed in the percentage of the total read length (e.g., 200 bases and 150 bases when raw input read lengths are 300 bases long)	66.67%	50.00%
Per base sequence content	Difference between AT and GC frequencies averaged at every read position. Since primer artifacts can cause fluctuations at the start of reads due to the non-random nature of enzymatic tagmentation when the Nextera XT protocol is used for library preparation, the first 20 bases are not included in this test. As fluctuations can also exist at the end of reads caused by the low abundance of very long reads because of read trimming, the 0.5% longest reads are similarly excluded	3	6
Minimum read length	Minimum read length after trimming (denoted as percentage of untrimmed read length) that minimum half of all trimmed reads must obtain (e.g., half of all trimmed reads should either be minimally 175 or 150 bases long when raw input reads lengths are 300 bases long)	58.33%	50%

#### Resistance Gene Characterization Assay

Genotypic antimicrobial resistance is detected by identifying resistance genes with nucleotide BLAST+ 2.6.0 (Camacho et al., 2009) using default values against four widely used resistance gene databases: ARG-ANNOT (Gupta et al., 2014), CARD (Jia et al., 2017), ResFinder (Zankari et al., 2012), and NDARO^3^. These databases are automatically pulled in-house and updated on a weekly basis (the date of the last database update is always included in the output). First, hits that cover less than 60% of, or have less than 90% identity to the subject, are removed. Second, overlapping hits (i.e., hits located on the same contig with at least one base overlap) are grouped into clusters. The best hit for each cluster is then determined using the method for allele scoring as described by Larsen et al. (2012). The different possibilities for “hit types” and their corresponding color codes used in the output are detailed in Supplementary Table S1. Visualizations of pair-wise alignments are extracted from the blast output generated with the pair-wise output format (“-outfmt 1”).

#### Sequence Typing Assay

Several relevant databases for sequence typing hosted by the PubMLST platform^4^ (Jolley and Maiden, 2010) are employed for genotypic sequence typing (Table 2). All sequences and profiles are obtained using the REST API (Jolley et al., 2017) and are automatically pulled in-house and updated on a weekly basis (the date of the last database update is always included in the output). For every database, loci are typed separately by aligning the assembled contigs against all allele sequences of that locus using Blastn and Blastx for nucleotide and protein sequences, respectively (Camacho et al., 2009). Filtering and best hit identification are performed as described previously for resistance gene characterization. If multiple exact matches exist, the longest one is reported. For protein sequences, alignment statistics are calculated based on the translated sequences. The different possibilities for “hit types” and their corresponding color codes used in the output are detailed in Supplementary Table S2. If sequence type definitions are available and the detected allele combination matches a known sequence type, this is reported along with associated metadata in the output (for classic MLST: corresponding clonal complex; for *rplF*: genospecies and associated comments). For *rpoB* (rifampicin resistance) and *penA* (penicillin resistance), the phenotypically tested susceptibility to the corresponding antibiotics of strains carrying that particular allele is also retrieved from PubMLST and included in the output.

**Table 2:** Overview of employed typing schemas^#^.

Schema name	#Total loci	# Nucleotide loci	# Protein loci
Classic MLST	7	7	0
*rplF*	1	1	0
cgMLST	1605	1605	0
Bexsero antigen sequence typing	5	0	5
*porA*	2	0	2
*porB*	1	1	0
*fetA*	1	0	1
*fHbp^∗^*	9	2	7
Resistance genes	9	9	0
Vaccine targets (*fHbp^∗^, nadA, nhba*)	3	3	0

*^#^All schemas are extracted from https://pubmlst.org (Jolley and Maiden, 2010; Jolley et al., 2017). A detailed overview of all their source links is provided in Supplementary Table S3. ^∗^*fHbp* is typed twice: for the vaccine targets as the whole gene (nucleotide), and for the *fHbp* schema as the whole gene (nucleotide) but also as a DNA fragment (nucleotide) and seven different peptides corresponding to, among others, the different protein segments.*

#### Serogroup Determination Assay

Serogroup determination is based on the genotypic sequence typing of the capsule loci (Harrison et al., 2013). Serogroup profiles are obtained from PubMLST using the REST API and are automatically pulled in-house and updated on a weekly basis (the date of the last database update is always included in the output). Serogroup profiles are available on PubMLST for the following 10 serogroups: A, B, C, E, H, L, W135, X, Y, and Z. Genotypic profiles for other serogroups are not available and can hence not be detected by the assay. The serogroups are assigned to categories based on the number and type of hits (see Supplementary Table S2) for the corresponding schemas. The first category contains serogroups for which all loci are found as perfect hits. In the second category, all loci are found as perfect or imperfect identity hits. In the third category, loci are detected as perfect hits, imperfect identity hits, imperfect short hits, or multi-hits. In the fourth category, not all loci are detected. The serogroup of the highest possible category is always reported. When there are multiple possible serogroups in the highest category, the serogroup with the highest fraction of perfect hits (i.e., number of perfect hits divided by number of loci in the schema) is reported. Serogroup determination fails when less than 75% of loci are detected for the best serogroup reported according to the above classification.

#### Implementation and Availability

The bioinformatics workflow was implemented in collaboration with the experts from the Belgian NRC *Neisseria* to ensure it complied with the needs of its actual end users by providing a “push-button” pipeline solution. On the front-end, the bioinformatics workflow was integrated as a stand-alone tool into a local instance of the Galaxy Workflow Management System (Afgan et al., 2016) to ensure a user-friendly interface that only requires uploading the data and selecting the desired bioinformatics assays (the data pre-processing and quality control are always executed by default). An illustration of the input interface is provided in Supplementary Figure S1. The bioinformatics workflow is compatible with data from all Illumina sequencing platforms (other sequencing technologies are not supported). Threshold values for some quality metrics such as the sequence length distribution (Table 1) are dynamically adapted based on the detected sequence length. Results are presented as an interactive user-friendly HTML output report and a tabular summary file. The HTML output report presents the results of all quality checks and bioinformatics assays, also containing linked data such as the trimmed reads, assembly, and all alignments, which can easily be accessed and/or downloaded by clicking the interactive link and can afterward be used for additional analyses, that are not part of the routine requirements, within Galaxy or other bioinformatics software. The tabular summary file contains an accumulation of the most important statistics and results in tab-separated format that can be useful for programmatic processing. On the back-end, the bioinformatics workflow was written in Python 2.7 and set up to comply with both the direct and indirect needs of the NRC. All required components (tools, databases, etc.) were directly integrated within the high-performance computational infrastructure at our institute. Employed public databases are pulled automatically in-house on a weekly basis to ensure that results are always up-to-date and that execution can be performed at all times without any direct dependencies on external resources (e.g., for outbreak situations). All tool parameters and options were optimized and validated (see below) to ensure that no parameter tweaking is required. The full workflow including all assays takes on average only 1 h to run to completion for a dataset sequenced at 60× coverage, which is only a fraction of the time compared to the data generation that can take multiple days. All individual components are version controlled and traceable, ranging from tool versions (managed through Lmod, available at https://github.com/TACC/Lmod), databases (managed through Git), in-house code (managed through Git), and workflow runs (managed through storing all essential information for rerunning the workflow in a custom-designed SQL database). This “push-button” pipeline is also available at the public instance of the Galaxy Workflow Management System of our institute, which is accessible at https://galaxy.sciensano.be. This is offered as a free resource for academic and non-profit usage (registration required), specifically intended for scientists and laboratories from other smaller European and/or developing countries that process a limited number of samples and/or do not have access to the required expertise and/or financial means themselves to analyze NGS data for *N. meningitidis* (with the caveat of depending on an external service for which 100% uptime cannot be guaranteed). A specific training video for this resource is also available (see Supplementary Material).

### Validation Data

#### Core Validation Dataset

The core validation dataset consisted out of *N. meningitidis* reference strains selected from the global collection of 107 *N. meningitidis* strains maintained at the University of Oxford (Bratcher et al., 2014) for which sequence data were generated in-house. Reference strains from this biobank were originally used to validate cgMLST at the genotypic level, and were extensively characterized using several sequencing technologies and platforms, thereby constituting a valuable resource that represents the global diversity of *N. meningitidis* for which high-quality genotypic information is available. A subset of 67 samples was selected by specialists from the Belgian NRC *Neisseria* to ensure that these cover the entire spectrum of clonal complexes that are representative for Belgium (see also the section “Discussion”). The selected samples were originally collected from 26 different countries over a timespan of more than 50 years encompassing endemic, epidemic, and pandemic disease cases, as well as asymptotic carriers. At least one sample was selected for each of the disease causing serogroups (A, B, C, W135, X, Y) (Harrison et al., 2009). An overview of these 67 samples is provided in Supplementary Table S4. Genomic DNA was extracted using the column-based GeneElute kit (Sigma), using the manufacturer’s instructions. Sequencing libraries were prepared with an Illumina Nextera XT DNA sample preparation kit and sequenced on an Illumina MiSeq instrument with a 300-bp paired-end protocol (MiSeq v3 chemistry) according to the manufacturer’s instructions. The 67 selected samples were sequenced three times in total. Runs A and B were performed on the same MiSeq instrument using newly created libraries from the samples, whereas run C was performed on a different MiSeq unit but using the same libraries as prepared for run B. Run A was done by a different operator than runs B and C. All WGS data generated for these samples in the three different runs have been deposited in the NCBI Sequence Read Archive (SRA) (Leinonen et al., 2011) under accession number SRP137803. Individual accession numbers for all sequenced samples for all runs are listed in Supplementary Table S14.

#### Extended Validation Dataset

The extended validation dataset consisted out of 64 *N. meningitidis* samples selected from publicly available NGS data. This additional dataset was collected to evaluate our bioinformatics workflow on data coming from different laboratories, as is often the case in real-world applications. In this extended dataset, we included additional strains of serogroups Y and W135, which are underrepresented in the global collection of 107 *N. meningitidis* samples maintained at the University of Oxford, and which are currently causing epidemics in both the United States and Europe (Mustapha et al., 2016; Whittaker et al., 2017) (see also the section “Discussion”). Additionally, the majority of samples in the extended validation dataset were generated by means of the HiSeq instrument in contrast to the MiSeq used for the core validation dataset, and read lengths for this dataset were consequently typically shorter. An overview of these samples with their corresponding NCBI SRA accession numbers is available in Supplementary Table S20.

### Validation of the Bioinformatics Workflow

#### Validation Strategy

We built upon previously described case studies (Lindsey et al., 2016; Kozyreva et al., 2017; Yachison et al., 2017; Holmes et al., 2018; Portmann et al., 2018) by implementing performance metrics that were adapted toward our purpose of exhaustively validating the bioinformatics workflow: repeatability, reproducibility, accuracy, precision, (diagnostic) sensitivity, and (diagnostic) specificity. A full overview of all performance metrics and their corresponding definitions and formulas is presented in Table 3. Some metrics were not evaluated for all bioinformatics assays, since it was not always possible to find suitable definitions in context of the specific analysis (see also the section “Discussion”). Precision specifically refers to the positive predictive value rather than repeatability and reproducibility as is the case in Kozyreva et al. (2017). “Within-run” replicates refer to duplicate (bioinformatics) analysis by executing the bioinformatics workflow twice on the same dataset for the calculation of repeatability. “Between-run” replicates refer to duplicate (bioinformatics) analysis by executing the bioinformatics workflow twice on the same sample generated on a different sequencing run for the calculation of reproducibility. Note that reproducibility could never be calculated for the extended validation dataset because no between-run replicates were available for these samples. The accuracy, precision, sensitivity, and specificity metrics all require the classification of results as either true positives (TPs), false positives (FPs), true negatives (TNs), or false negatives (FN), which by definition all require the comparison against a reference or standard that represents the “truth,” for which we adopted two reference standards. First, genotypic information for the reference strains from the core validation dataset available in the PubMLST isolate database (accessible at https://pubmlst.org/bigsdb?db=pubmlst_neisseria_isolates) was used to compare results from our bioinformatics workflow against, which was referred to as “database standard.” Second, because no such high-quality genotypic information was available for the samples from the extended validation dataset with the sole exception of the serogroup, results of our bioinformatics workflow were compared against the results of tools commonly used and adopted by the scientific community, which was referred to as “tool standard.” This second approach was also employed for the core validation dataset to evaluate consistency between both standards, and also because for the resistance gene characterization assays no genotypic information was available in the associated reference database for the core validation dataset. All analyses were done through a local “push-button” implementation of the bioinformatics workflow (see the section “Implementation and Availability”). All output files were downloaded upon completion and the performance of the different bioinformatics assays was evaluated by querying the output files using in-house scripts that collected the performance metrics presented in Table 3. All output reports of the bioinformatics workflow are publicly available at Zenodo^5^.

**Table 3:** Overview of performance metrics and their corresponding definitions and formulas adopted for our validation strategy.

			Assay-specific
Metric	Definition	Formula	definitions	Bioinformatics assay					
				Resistance gene characterization				Sequence typing (cgMLST)	Serogroup determination
				ARG-ANNOT	ResFinder	CARD	NDARO		
Repeatability	Agreement of the assay based on within-run replicates	Repeatability = 100% × (# within-run replicates in agreement)/(total # within-run replicates)	Within-run replicate	Repeated bioinformatics analysis on the same sample using the same dataset					
Reproducibility	Agreement of the assay based on between-run replicates	Reproducibility = 100% × (# between-run replicates in agreement)/(total # between-run replicates)	Between-run replicate	Repeated bioinformatics analysis on the same sample using a different dataset (generated using a different library and/or sequencing run)					
Accuracy	The likelihood that results of the assay are correct	Accuracy = 100% × (TP+TN)/(TN+FN+TP+FP)	TP result	Detection of a gene present in the reference standard				Detection of the same allele as in the reference standard	Detection of the same serogroup as in reference standard
Precision	The likelihood that detected results of the assay are truly present	Precision = 100% × TP/(TP+FP)	FN result	No detection of a gene present in the reference standard				Detection of a different allele as in the reference standard	Detection of a different serogroup as in the reference standard
Sensitivity	The likelihood that a result will be correctly picked up by the assay when present	Sensitivity = 100% × TP/(TP+FN)	TN result	No detection of a gene not present in the reference standard				No detection of an allele when challenged with the cgMLST schema of *L. monocytogenes*	No detection of a serogroup when challenged with the serogroup schema of *L. monocytogenes*
Specificity	The likelihood that a result will not be falsely picked up by the assay when not present	Specificity = 100% × TN/(TN+FP)	FP result	Detection of a gene not present in the reference standard				Detection of an allele when challenged with the cgMLST schema of *L. monocytogenes*	Detection of a serogroup when challenged with the serogroup schema of *L. monocytogenes*

*TP, true positive; TN, true negative; FP, false positive; FN, false negative.*

#### Resistance Gene Characterization Assay

The reportable range for all three databases corresponds to their respective gene content, and performance was evaluated at the level of the gene. For repeatability and reproducibility, replicates were considered to be in agreement when a gene (also including imperfect hits) was detected or absent in within-run and between-run replicates, respectively. For the database standard, no metrics could be calculated as no such associated information is available for neither the core nor extended validation datasets. For the tool standard, no accompanying reference tool exists for the ARG-ANNOT database, for which performance metrics could therefore not be calculated. For the CARD database, Resistance Gene Identifier (RGI) 4.0.3 (Jia et al., 2017) was used as the tool standard. The program was executed on all assemblies using the following settings: database version 1.1.8, input type parameter set to “contig,” alignment tool set to BLAST (all other settings were left at their default values). Loose hits, hits that covered less than 60% of the query sequence or with less than 90% sequence identity, and hits that aligned to the protein variant model were afterward excluded. For the ResFinder database, the online web service^6^ was used as the reference tool, by analyzing all assemblies using the following settings: 90% identity threshold, 60% minimum length, and all antimicrobial resistance databases selected. For the NDARO database, AMRfinder (alpha version) was used as the tool standard (excluding hits covering less than 60% of the target gene). The following definitions for classification were used: TP as genes detected by both our workflow and the tool standard; FN as genes missed by our workflow but reported by the tool standard; FP as genes detected by our workflow but not reported by the tool standard; and TN as genes not detected by both our workflow and the tool standard.

#### Sequence Typing Assay

The reportable range for all sequence typing schemas corresponds to their respective gene content (see Table 2 and Supplementary Table S3). cgMLST was used as a proxy for the performance of all typing schemas because this schema contains 1605 loci, whereas no single other typing schema included in our workflow contains more than 9 loci (Table 2), so that too few observations would be present to employ the formulas presented in Table 3. Similar to resistance gene characterization, a gene-by-gene approach was taken for calculating performance. For repeatability and reproducibility, replicates were considered to be in agreement when no allele was detected or the same allele was detected as a perfect hit in within-run and between-run sequencing replicates, respectively. Database reference information was extracted directly from the PubMLST isolate database (see Supplementary Table S3 for exact source locations), whereas tool standard information was obtained by using the assemblies as input for the online PubMLST sequence query tool (https://pubmlst.org/bigsdb?db=pubmlst_neisseria_seqdef&page=sequenceQuery). The following definitions for classification were used: TP and FN as alleles where the output of our workflow corresponded, or did not correspond, to the database reference, respectively. In case the database standard contained several alleles for a locus, results were considered concordant as soon as one of them matched with our workflow. TN and FP were evaluated by querying the assemblies against a cgMLST database for an unmatched species for which the sequence typing assay is not expected to identify any alleles (Kozyreva et al., 2017). We employed the *L. monocytogenes* cgMLST schema that is available through BIGSdb hosted at the Institut Pasteur (Moura et al., 2016). TN and FP were defined as unidentified and identified alleles by our workflow, respectively. We verified this approach by checking all assemblies with the sequence query tool of BIGSdb hosted at the Institut Pasteur against the *L. monocytogenes* cgMLST schema, for which never any allele was reported.

#### Serogroup Determination Assay

The reportable range for the serogroup determination assay corresponds to the 10 serogroups for which a schema with capsule loci exists in the PubMLST database (see Supplementary Table S3). Performance was evaluated at the level of the serogroup. For repeatability and reproducibility, replicates were considered to be in agreement when the same or no serogroup was detected in within-run and between-run replicates, respectively. Database standard information was extracted directly from the PubMLST isolate database (see Supplementary Table S3 for exact source location), whereas tool standard information was obtained by using the assemblies as input for the online PubMLST sequence query tool (https://pubmlst.org/bigsdb?db=pubmlst_neisseria_seqdef&page=sequenceQuery). The following definitions for classification were used: TP and FN as serogroups where the output of our workflow corresponded, or did not correspond, to the reference. Note that for the tool standard, however, the sequence query tool of PubMLST does not output serogroups but rather results for all corresponding capsule loci. We therefore considered a serogroup to be detected when all its corresponding loci were detected, and samples for which this was not the case were considered as missing data. TN and FP were evaluated by querying the assemblies against a cgMLST database for an unmatched species for which the serogroup determination assay is not expected to identify any serogroups (Kozyreva et al., 2017). We employed the *L. monocytogenes* serogroup schema that is available through BIGSdb hosted at the Institut Pasteur (Moura et al., 2016). TN and FP were defined as unidentified and identified serogroups by our workflow, respectively. We verified this approach similarly by checking all assemblies with the sequence query tool of BIGSdb hosted at the Institut Pasteur against the *L. monocytogenes* serogrouping schema, for which never any locus of the capsule loci was reported.

### Supplementary Material

A supplementary manuscript containing all supplementary figures and tables is available as “Bogaerts_Neisseria_ supplementaryMaterial.docx.” A supplementary video providing a tutorial for employing the “push-button” pipeline instance of our bioinformatics workflow is also available at Zenodo^7^.

## Results

### Evaluation of Sequence Data Quality

#### Core Validation Dataset

Read counts for raw and trimmed reads for all 67 samples of the core validation dataset in each of the three separate sequencing runs are provided in Supplementary Figure S4 and Supplementary Table S5. The number of raw reads per sample is in the same range for all runs with medians of 341,547; 338,107, and 354,874 paired-end reads for runs A, B, and C, respectively, although run A displayed more variation in the number of reads per sample. The fraction of forward and reverse reads surviving trimming is generally high with medians of 76.91%, 88.83%, and 90.49% for runs A, B, and C, respectively. A larger fraction of forward reads always survived trimming, indicating the reverse reads are generally of lower quality. Assembly statistics for all samples and runs are provided in Supplementary Figure S5 and Supplementary Table S6. The N50, a metric used as a proxy for assembly quality that is defined as the length at which contigs of equal or longer length contain at least 50% of the assembled sequence (Lander et al., 2001), is comparable across all runs with medians of 49,389, 54,223, and 56,526 bases for runs A, B, and C, respectively. Assemblies of run A contained substantially lower numbers of contigs compared to runs B and C, with medians of 182, 822, and 832 contigs for runs A, B, and C, respectively. Results for all runs are, however, comparable with medians of 87, 85, and 82 contigs for runs A, B, and C, respectively, when only contigs >1,000 bases are considered. Sample Z4242 in run A is an outlier for both N50 (8,743 bases) and number of contigs >1,000 bases (295 contigs), indicating a highly fractured assembly. More contigs were hence generated for all samples in runs B and C compared to run A, but these were typically <1,000 bases so that the overall N50 is similar for all runs. Advanced quality statistics for all samples and runs are provided in Supplementary Figure S6 and Supplementary Table S7. The coverage for samples in run A displays more variation and is lower compared to runs B and C with medians of 45, 60, and 65× for runs A, B, and C, respectively, and are above recommendations from other studies that indicated values of 20×–40× are required for high-quality results (Lindsey et al., 2016). The percentage of cgMLST genes identified was very high across all runs with medians of 97.76, 97.94, and 97.94% for runs A, B, and C, respectively, close to the mean observation of 98.8% across five different laboratories during a ring trial for *Staphylococcus aureus* (Mellmann et al., 2017). Sample Z4242 in run A is again an outlier (see before), as it is the only sample in all three runs with <95% identified cgMLST genes. The mapping rate was generally also very high with medians of 99.25, 98.30, and 98.30% for runs A, B, and C, respectively, with the exception of sample Z4242 in run A. All samples for all runs passed all quality checks listed in Table 1, with some warnings but never a failure for any metric.

#### Extended Validation Dataset

Read counts for raw and trimmed reads for all 64 samples of the extended validation dataset are provided in Supplementary Figure S7 and Supplementary Table S21. The median number of raw reads across all samples is 1,826,497. The fraction of forward and reverse reads surviving read trimming is very high with a median value of 99.58% across all samples. These observations are in line with the higher coverage and shorter read lengths provided by the HiSeq sequencing instrument for samples of the extended validation dataset. Assembly statistics for all samples are provided in Supplementary Figure S8 and Supplementary Table S22. The median values for the N50, number of contigs, and number of contigs >1,000 bases are 52,341 bases, 650 contigs, and 170 contigs, respectively. These numbers indicate that the assemblies are more fragmented compared to the core validation dataset, as can be expected through their shorter read length. Three samples, ERR314131, ERR314115, and ERR278691, displayed however extremely fragmented assemblies with a N50 <1,000 bases. Advanced quality statistics for all samples are provided in Supplementary Figure S9 and Supplementary Table S23. The median coverage, percentage of identified cgMLST genes, and mapping rate are 142×, 97.82%, and 99.58%, respectively. Samples ERR314131, ERR314115, and ERR278691 did not have a single cgMLST allele detected, in line with their exceptionally low N50, and also failed multiple of the quality checks mentioned in Table 1. All other samples passed all of these quality checks, the mean *Q*-score drop being the only exception for some samples that had a more pronounced *Q*-score dip at the beginning of reads.

### Evaluation of Performance Metrics

#### Resistance Gene Characterization Assay

Table 4 presents results for the core validation dataset for the three different databases, and a detailed overview of all detected genes for all three databases is available in Supplementary Table S8. For the ARG-ANNOT database, within-run replicates were always fully consistent, resulting in a repeatability of 100%. No perfect hits were detected in any samples of any run. A full-length imperfect hit with 99.67% identity to *tetB* was found in sample Z5826 for all sequencing runs. Another imperfect hit, covering 71.79% of *ermF* with 99.83% identity, was found for sample Z1073 in run B only. As this results in only two between-run replicates not in agreement on a total of 128,439 comparisons (i.e., 639 database genes times 67 samples times 3 runs), the reproducibility rounds up to 100%. Performance metrics could not be calculated for the database standard because no such information was available, nor for the tool standard as a reference tool does not exist for ARG-ANNOT. For the ResFinder database, within-run replicates were always fully consistent, resulting in a repeatability of 100%. No perfect hits were detected in any samples of any run. Similar to ARG-ANNOT, a full length imperfect hit with 99.92% identity to *tetB* was found in sample Z5826 for all sequencing runs. The identity differs slightly between ResFinder and ARG-ANNOT for *tetB* because a different variant is found in both databases. Another imperfect hit, covering 71.79% of *ermF* with 99.83% identity, was found for sample Z1073 in run B only. As this results in only two between-run replicates not in agreement on a total of 106,932 comparisons (i.e., 532 database genes times 67 samples times 3 runs), the reproducibility rounds up to 100%. Performance metrics could not be calculated for the database standard because no such information was available. For the tool standard, all detected genes were always fully consistent with results of the ResFinder web tool. The ensuing confusion matrix (i.e., a contingency table that lists the performance based on actual and predicted classes) is presented in Supplementary Table S15, and results in a perfect accuracy, precision, sensitivity, and specificity of 100%. For the CARD database, within-run replicates were always fully consistent, resulting in a repeatability of 100%. Four genes were detected in several samples as perfect hits for all runs: *farA, farB, mtrC*, and *mtrD*. An imperfect hit, covering 71.79% of *ermF* with 99.83% identity, was found for sample Z1073 in run B only. Another imperfect hit, covering 71.79% of *mtrR* with 99.83% identity, was detected in runs B and C but not in run A for sample Z4242. As these were the only four between-run replicates not in agreement on a total of 158,187 comparisons (i.e., 787 database genes times 67 samples times 3 runs), the reproducibility rounds up to 100%. Performance metrics could not be calculated for the database standard because no such information was available. For the tool standard, all detected genes were always fully consistent with results of the RGI tool, with the exception of *mtrE* and *aac*(*2*′). *mtrE* was detected in all samples across all runs by our workflow but never by RGI, and was therefore classified as a FP in all samples. This was most likely because the *mtrE* sequence displayed very high nucleotide identity (∼94%) with the database variant, but had some frameshift mutations. Since RGI predicts open reading frames followed by protein alignment of the translated sequences, it did never detect *mtrE*. *aac*(*2*′) was not detected by our workflow in sample Z1092 in run B, and was therefore classified as a FN. The ensuing confusion matrix is presented in Supplementary Table S15, and results in an accuracy, precision, sensitivity, and specificity of 99.87, 87.52, 99.93, and 99.87%, respectively. For the NDARO database, within-run replicates were always fully consistent, resulting in a repeatability of 100%. No perfect hits were detected in any samples of any run. Similar to ARG-ANNOT and ResFinder, a full length imperfect hit with 99.93% identity to *tetB* was found in sample Z5826 for all sequencing runs. The identity differs slightly between databases due to different variants that are present in the databases. Another imperfect hit, covering 63.82% of *ermF* with 99.65% identity, was found for sample Z1073 in run B only (corresponding to the ResFinder output for the same sample). The *oxa* gene was found in five more samples (Z4690 run C, Z4707 run B, Z5043 run C, Z6414 run B, and Z6430 run B) with over 99% identity and covering around 65% of the reference sequence. As this results in a total of 12 between-run replicates not in agreement on a total number of 180,699 comparisons (i.e., 899 database genes times 67 samples times 3 runs), the reproducibility rounds up to 100%. Performance metrics could not be calculated for the database standard because no such information was available. For the tool standard, all detected genes were always fully consistent with results of the AMRfinder tool. The ensuing confusion matrix is presented in Supplementary Table S15, and results in an accuracy, precision, sensitivity, and specificity of 100%.

**Table 4:** Results for the core validation dataset.

Metric		Bioinformatics assay					
						Sequence typing	Serogroup
		Resistance gene detection				(cgMLST)	determination
		ARG-ANNOT	ResFinder	CARD	NDARO		
Repeatability		100%	100%	100%	100%	100%	100%
Reproducibility		100%	100%	100%	100%	99.65%	99.50%
Database standard	Accuracy	–	–	–	–	98.62%	95.27%
	Precision	–	–	–	–	100%	100%
	Sensitivity	–	–	–	–	97.12%	90.55%
	Specificity	–	–	–	–	100%	100%
Tool standard	Accuracy	–	100%	99.87%	100%	99.37%	100%
	Precision	–	100%	87.52%	100%	100%	100%
	Sensitivity	–	100%	99.93%	100%	98.68%	100%
	Specificity	–	100%	99.87%	100%	100%	100%

*A dash (“–”) indicates a performance metric could not be calculated/determined.*

Table 5 presents results for the extended validation dataset for the three different databases. A detailed overview of all detected genes for all three databases can be found in Supplementary Table S24. Within-run replicates were always fully consistent, resulting in a repeatability of 100%. Reproducibility could not be assessed as no between-run replicates were available. No perfect hits were detected for any of the resistance gene databases, but several imperfect hits were found. As for the core validation dataset, performance metrics could only be calculated for the tool standard for the ResFinder, CARD, and NDARO databases, and the resulting confusion matrices are presented in Supplementary Table S28. For the NDARO database, not a single gene was detected in the extended validation dataset by both our workflow and AMRfinder, meaning that precision and sensitivity could not be calculated. For the ResFinder database, a perfect accuracy, precision, sensitivity, and specificity of 100% were obtained. For the CARD database, there were again several FP that were all due to the *mtrE* gene being detected in several samples, resulting in an accuracy, precision, sensitivity, and specificity of 99.88, 87.50, 100, and 99.88%, respectively. Results for both databases were therefore in line with results observed for the tool standard for the core validation dataset (Table 4).

**Table 5:** Results for the extended validation dataset.

Metric		Bioinformatics assay					
						Sequence typing	Serogroup
		Resistance gene detection				(cgMLST)	determination
		ARG-ANNOT	ResFinder	CARD	NDARO		
Repeatability		100.00%	100.00%	100.00%	100.00%	100.00%	100.00%
Reproducibility		–	–	–	–	–	–
Database standard	Accuracy	–	–	–	–	–	96.09%
	Precision	–	–	–	–	–	100.00%
	Sensitivity	–	–	–	–	–	92.19%
	Specificity	–	–	–	–	–	100.00%
Tool standard	Accuracy	–	100.00%	99.88%	100.00%	99.78%	100.00%
	Precision	–	100.00%	87.50%	^∗^	100.00%	100.00%
	Sensitivity	–	100.00%	100%	^∗^	99.52%	100.00%
	Specificity	–	100.00%	99.88%	100.00%	100.00%	100.00%

*A dash (“–”) indicates a performance metric could not be calculated/determined because the data were unavailable. An asterisk (^∗^) indicates that the metric could not be calculated, due to division by zero.*

#### Sequence Typing Assay

**FIGURE 2:**
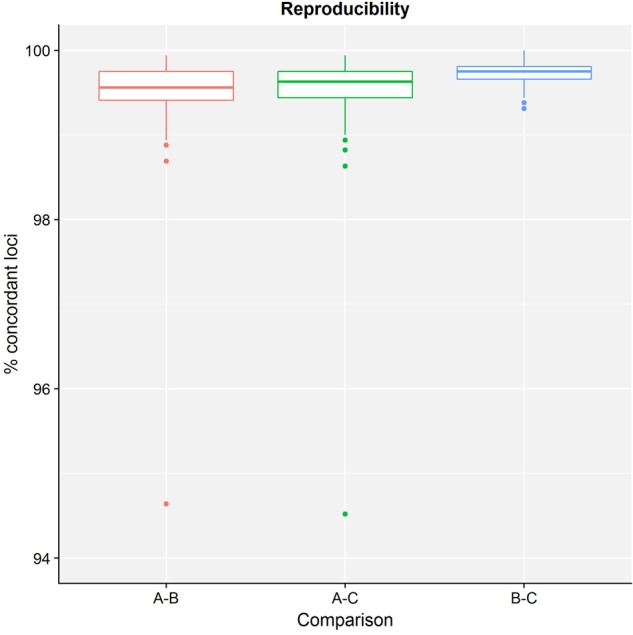
Reproducibility of the sequence typing assay for the core validation dataset. The abscissa depicts the sequencing runs that are being compared, while the ordinate represents the percentage of cgMLST loci that were concordant between the same samples of different sequencing runs. Note that the ordinate starts at 94% instead of 0% to enable illustrating the variation between run comparisons more clearly. Each comparison is presented as a boxplot based on 67 samples where the boundary of the box closest to the abscissa indicates the 25th percentile, the thick line inside the box indicates the median, and the boundary of the box farthest from the abscissa indicates the 75th percentile. See also Supplementary Table S9 for detailed values for all samples and sequencing runs.

**FIGURE 3:**
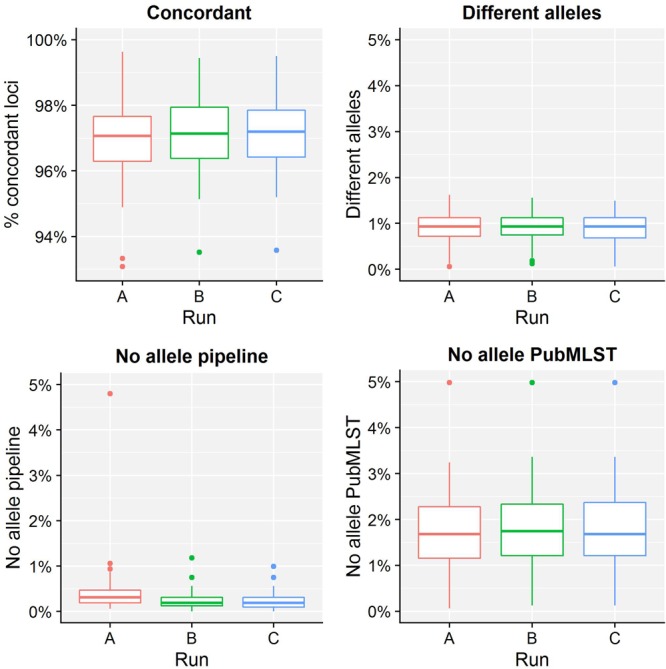
Database standard results of the sequence typing assay for the core validation dataset. The abscissa depicts the sequencing run, while the ordinate represents the percentages of cgMLST loci as indicated by the title above each graph. Each sequencing run is presented as a boxplot based on 67 samples (see the legend of Figure 2 for a brief explanation). The upper left graph depicts the percentage of concordant cgMLST loci, i.e., where our workflow identified the same allele as the database standard, which were classified as TPs. Note that the ordinate starts at 93% instead of 0% to enable illustrating the results more clearly. All other cases were classified as FNs, and encompass three categories. First, the upper right graph depicts the percentage of cgMLST loci for which our workflow detected a different allele than present in the database standard. Second, the bottom left graph depicts the percentage of cgMLST loci for which our workflow did not detect any allele but an allele was nevertheless present in the database standard. Third, the bottom right graph depicts the percentage of cgMLST loci for which our workflow detected an allele but for which no allele was present in the database standard. Most FNs are explained by no information being present in the database standard, followed by an actual mismatch, and only few cases are due to our workflow improperly not detecting an allele. See also Supplementary Table S10 for detailed values for all samples and runs.

**FIGURE 4:**
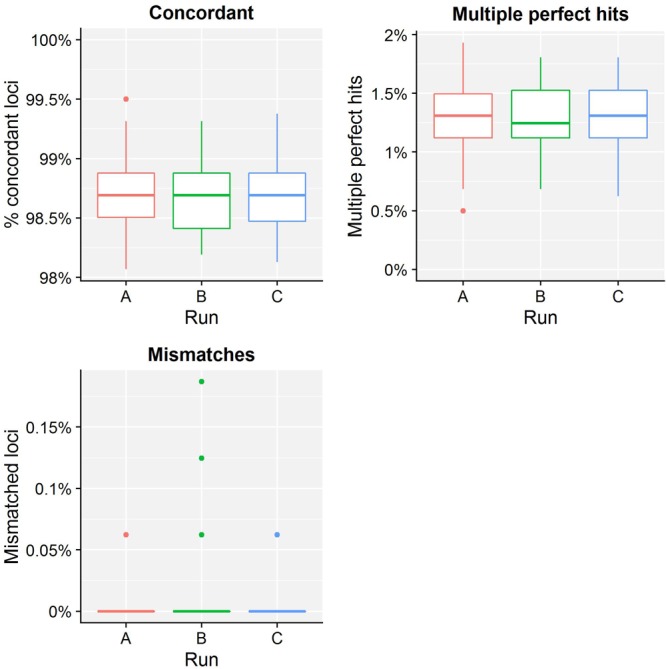
Tool standard results of the sequence typing assay for the core validation dataset. The abscissa depicts the sequencing run, while the ordinate represents the percentages of cgMLST loci as indicated by the title above each graph. Each sequencing run is presented as a boxplot based on 67 samples (see the legend of Figure 2 for a brief explanation). The upper left graph depicts the percentage of concordant cgMLST loci, i.e., where our workflow identified the same allele as the tool standard, which were classified as TPs. Note that the ordinate starts at 98% instead of 0% to enable illustrating the results more clearly. All other cases were classified as FNs, and encompass two categories. First, the upper right graph depicts the percentage of cgMLST loci for which our workflow identified multiple perfect hits, of which at least one corresponded to the tool standard but was reported differently. Second, the lower left graph depicts the percentage of cgMLST loci for which our workflow detected a different allele compared to the tool standard. Most FNs are therefore explained by a different manner of handling multiple perfect hits, and only a small minority are due to an actual mismatch between our workflow and the tool standard. Furthermore, upon closer inspection, these mismatches were due to an artifact of the reference tool used for the tool standard that has been resolved in the meantime (see Supplementary Figure S2). See also Supplementary Table S11 for detailed values for all samples and runs.

Table 4 presents results for the core validation dataset, using cgMLST as a proxy for the performance of all sequence typing schemas (see the section “Materials and Methods”). Within-run replicates were always fully consistent, resulting in a repeatability of 100%. Results for between-run replicates are presented in Figure 2 and Supplementary Table S9. The median reproducibility is 99.56% for run A versus B, 99.63% for run A versus C, and 99.75% for run B versus C, with a reproducibility of 99.65% averaged over all three comparisons. One outlier is present for the comparisons of run A versus B and run A versus C, which is in both cases due to sample Z4242 in run A that only had 94.14% of its cgMLST loci identified in the first place. Because results for this sample were relatively bad in run A compared to runs B and C, we checked whether contamination had occurred but could find no indication thereof (see Supplementary Figure S3). For the database standard, results are presented in Figure 3 and Supplementary Table S10. TP were defined as loci of the cgMLST schema where our workflow identified the same allele as the database standard, with median values of 97.07, 97.13, and 97.20% of cgMLST loci for runs A, B, and C, respectively. All other loci were classified as FN, of which the majority were due to cgMLST loci being identified by our workflow while no information was available in the database standard rather than an actual mismatch between our workflow and the database standard (Figure 3). Values for TN and FP were put at 100% and 0% of cgMLST loci, respectively, to reflect that the database standard represents the “truth” (see also the section “Discussion”). The ensuing confusion matrix is presented in Supplementary Table S16, and results in an accuracy, precision, sensitivity, and specificity of 98.62, 100, 97.12, and 100%, respectively. For the tool standard, results are presented in Figure 4 and Supplementary Table S11. TP were defined as loci of the cgMLST schema where our workflow identified the same allele as the PubMLST sequence query tool, with medians of 98.69, 98.69, and 98.69% of cgMLST loci for runs A, B, and C, respectively. All other loci were classified as FN, of which the majority were due to multiple alleles being identified by our workflow, in which case the longest one is reported in contrast to the reference tool where an allele is picked randomly, rather than an actual mismatch between our workflow and the tool standard (Figure 4). TN and FP were defined as alleles correctly unidentified, or falsely identified, when challenged with the cgMLST schema of *L. monocytogenes*, for which neither our workflow nor the reference tool, however, ever picked up a single allele resulting in values for TN and FP of 100 and 0% of cgMLST loci, respectively. The ensuing confusion matrix is presented in Supplementary Table S17, and results in an accuracy, precision, sensitivity, and specificity of 99.37, 100, 98.68, and 100%, respectively.

Table 5 presents results for the extended validation dataset for the cgMLST assay. Within-run replicates were always fully consistent, resulting in a repeatability of 100%. Reproducibility could not be assessed as no between-run replicates were available. Performance metrics could neither be calculated for the database standard because no such information was available. Results for the tool standard are detailed in Supplementary Table S25. Our workflow identified the same allele as the tool standard for 99.50% of cgMLST loci that were classified as TP. All other loci were classified as FN. No alleles were detected by our workflow nor the reference tool when challenged with the cgMLST schema of *L. monocytogenes* resulting in values for TN and FP of 100 and 0% of cgMLST loci, respectively. The ensuing confusion matrix is presented in Supplementary Table S29, and results in an accuracy, precision, sensitivity, and specificity of 99.78, 100, 99.52, and 100%, respectively, which is in line with results observed for the tool standard for the core validation dataset.

#### Serogroup Determination Assay

Table 4 presents results for the serogroup determination assay for the core validation dataset. Within-run replicates were always fully consistent, resulting in a repeatability of 100%. Between-run replicates were also fully consistent with the exception of sample Z4242 for which serogroup B was detected in run A but serogroup C in runs B and C. Serogroups B and C share four common loci with one and two additional unique loci, respectively. Because this unique locus was not detected due to the low quality of sample Z4242 in run A (see before), it was classified as serogroup B, resulting in a reproducibility of 99.50%. For the database standard, results are presented Supplementary Table S12. TP were defined as samples where our workflow identified the same serogroup as the database standard, which resulted in 89.55, 91.04, and 91.04% correctly predicted serogroups for runs A, B, and C, respectively. All other samples were classified as FN. Values for TN and FP were put at 100 and 0% of serogroups, respectively, to reflect that the database standard represents the “truth” (see also the section “Discussion”). The ensuing confusion matrix is presented in Supplementary Table S18, and results in an accuracy, precision, sensitivity, and specificity of 95.27, 100, 90.55, and 100%, respectively. For the tool standard, results are presented Supplementary Table S13. TP were defined as samples for which our workflow identified the same serogroup as the tool standard, which resulted in values of 100% for all runs so that no single sample was classified as FN. TN and FP were defined as samples where the serogroup was correctly unidentified, or falsely identified, when challenged with the serogroup schema of *L. monocytogenes*, for which neither our workflow nor the reference tool, however, ever picked up a single capsule locus resulting in values for TN and FP of 100 and 0% of samples, respectively. The ensuing confusion matrix is presented in Supplementary Table S19, and results in a perfect accuracy, precision, sensitivity, and specificity of 100%.

Table 5 presents results for the extended validation dataset for the serogroup determination assay. Within-run replicates were always fully consistent, resulting in a repeatability of 100%. Reproducibility could not be assessed as no between-run replicates were available. Results for the database standard are presented in Supplementary Table S26. Our workflow identified the same serogroup as the database standard for 92.19% of samples that were classified as TP. All other samples were classified as FN. Values for TN and FP were put at 100 and 0% of samples, respectively, to reflect that the database standard represents the “truth” (see also the section “Discussion”). The ensuing confusion matrix is presented in Supplementary Table S30 and results in an accuracy, precision, sensitivity, and specificity of 96.09, 100, 92.19, and 100%, respectively. Results for the tool standard are presented in Supplementary Table S27. Our workflow identified the same serogroup as the tool standard for 100% of samples that were classified as TP, and no samples were classified as FN. No capsule loci were detected by our workflow nor the reference tool when challenged with the serogroup schema of *L. monocytogenes* resulting in values for TN and FP of 100 and 0% of samples, respectively. The ensuing confusion matrix is presented in Supplementary Table S31, and results in a perfect accuracy, precision, sensitivity, and specificity of 100%. Results for both the database and tool reference were therefore in line with results observed for the core validation dataset (Table 4).

## Discussion

We report here the first exhaustive validation of a bioinformatics workflow for clinical microbiological isolate WGS data. We employed the pathogen *N. meningitidis* as a proof-of-concept by designing a bioinformatics workflow (Figure 1) that incorporates different quality checks (Table 1) and relevant typing schemas (Table 2) with the aim of either extracting information that ensures backward compatibility with currently existing “classical” molecular biology techniques (e.g., serotyping), or alternatively taking advantage of the full potential offered by WGS by extracting information at the scale of the full genome (e.g., cgMLST). Our study is relevant because recent surveys by both the EFSA (García Fierro et al., 2018) and the ECDC (Revez et al., 2017) have indicated that, at least in Europe, the data analysis and required expertise remain substantial bottlenecks impeding the implementation of NGS for routine use in microbiology. For NRCs and NRLs, as well as other laboratories working under a quality system, a harmonized framework for validation of the WGS workflow presents an additional obstacle (Rossen et al., 2018). A series of recently published studies have, however, showcased the need thereof, and presented validation approaches for certain components of the WGS workflow focusing either on a modular template for the validation of WGS processes (Kozyreva et al., 2017), the entire workflow “end-to-end” (Portmann et al., 2018), standardization (Holmes et al., 2018), external quality assessment (Mellmann et al., 2017), commercial bioinformatics software (Lindsey et al., 2016), outbreak clustering (Dallman et al., 2015), or specific assays such as serotyping (Yachison et al., 2017). We complement these studies by proposing a validation strategy focusing specifically on the bioinformatics analysis of the WGS workflow to exhaustively evaluate performance at this level, which is crucial because the bioinformatics component serves as the “common denominator” that allows to compare the different steps of the WGS workflow (e.g., library preparation, sequencing, etc.) or even different WGS workflows and/or sequencing technologies. Although workflow components and quality metrics listed in Figure 1 and Table 1 will need to be adapted, the validation strategy proposed in our study is platform-agnostic and can be tailored toward other sequencing technologies. The underlying premise of our validation strategy consists of demonstrating that the bioinformatics workflow is “fit-for-purpose,” which is defined by the ISO17025 standard as providing “confirmation by examination and provision of objective evidence that the particular requirements for a specific intended use are fulfilled” (ISO/IEC 17025:2005). We addressed this by employing several classical performance metrics with definitions and formulas adapted specifically toward the nature of the different bioinformatics assays (Table 3), which were evaluated on both a core and extended validation dataset. The core validation dataset was constructed by means of in-house sequencing of a selection of 67 samples from the global collection of 107 *N. meningitidis* strains maintained at the University of Oxford (Bratcher et al., 2014), because it contains strains that encompass much of the genetic diversity encountered within Belgium and therefore can be considered as representative for our country. Moreover, this collection has been extensively characterized and high-quality genotypic information is available for many relevant assays such as cgMLST and serogrouping, thereby providing a database standard to compare results of our bioinformatics workflow against. The extended validation dataset was composed by selecting 64 samples from publicly available NGS data, and therefore allowed to expand the validation scope to genotypes underrepresented in this reference collection and/or sequenced through different (Illumina) platforms. The Y and W135 serogroups are a notable example of recently endemic cases in Belgium that are underrepresented in the global collection of 107 *N. meningitidis* samples. Because no high-quality genotypic information was however available for the sequence typing assay for this dataset, we employed an alternative reference based on results obtained through bioinformatics tools and resources commonly used and adopted by the scientific community (Hendriksen et al., 2018), such as the suite of tools for pathogen typing and characterization of the Center for Genomic Epidemiology (Deng et al., 2016) and PubMLST (Jolley and Maiden, 2010), which were used as a tool standard to compare the results of our bioinformatics workflow against. The same approach was also taken for the core validation dataset to evaluate consistency between the database and tool standard, and because high-quality genotypic information was not available for all bioinformatics assays of the core validation dataset such as resistance gene characterization.

Results for the different performance metrics are presented in Tables 4, 5 for the core and extended validation datasets, respectively, and generally demonstrate high to very high performance in line with results obtained from other case studies (Lindsey et al., 2016; Kozyreva et al., 2017; Yachison et al., 2017; Holmes et al., 2018; Portmann et al., 2018). Repeatability and reproducibility were defined as agreement of within-run and between-run replicates, and evaluate concordance between runs of the bioinformatics workflow on either the same NGS dataset, or a different NGS dataset that was generated for the same sample and/or library, respectively. Repeatability was always 100% for all assays for both the core and extended validation datasets. Although certain components of the workflow employ heuristics to accelerate the computation, they do not appear to have an effect on the final results. Reproducibility could only be evaluated for the core validation dataset, and was also found to be very high reaching values of 100, 99.65, and 99.50% for the resistance gene characterization, sequence typing, and serogroup determination assays, respectively. The small number of discrepancies between sequencing runs could be traced back to differences between runs A and B/C, whereas results of runs B and C were always much more concordant, as illustrated for the sequence typing assay in Figure 2. This could potentially indicate a library preparation effect because runs B and C share the same libraries, sequencing instrument, and operator, but the difference is too small to make any definitive observations and could also be explained by stochastic variation or the lower quality of some samples in sequencing run A (see the section “Results”). Accuracy and precision were defined as the likelihoods that results are correct and truly present, respectively. Sensitivity and specificity were defined as the likelihoods that a result will be correctly picked up when present, and not falsely be picked up when not present, respectively. These four performance metrics all require the classification of results of the different bioinformatics assays as either TP, FN, TN, or FP, for which assay-specific definitions were formulated (Table 3). Classification also requires to use a reference that represents the best possible approximation of the “truth,” for which we used either a database or tool standard (see above). This approach differs from the one employed by Kozyreva et al. (2017), where both standards were used without discrimination for the validation of the bioinformatics component, but for which we consider differentiation relevant to indicate whether performance is evaluated on high-quality genotypic information or rather widely used and adopted bioinformatics tools. Note that our implementation of accuracy for bioinformatics assays also differs from other studies where this was defined as all correct results divided by all results (Kozyreva et al., 2017; Mellmann et al., 2017; Yachison et al., 2017), whereas we adopted the more classically employed definition of all correct classifications (TP + TN) divided by all classifications (TP+FN+TN+FP) (Hardwick et al., 2017), thereby also incorporating results from the negative controls into the calculation of this metric. Additionally, we introduced precision as a performance metric rather than using this term to refer to both repeatability and reproducibility (Kozyreva et al., 2017). This metric is of particular interest because it does not incorporate TN and is more informative than the sensitivity for bioinformatics assays for which an imbalance between the number of positive and negative classes exists, such as is for instance the case for all resistance gene characterization assays. These different performance metrics therefore all provide complementary information, for which it can be helpful to consider that “accuracy and precision provide insight into completeness, whereas sensitivity and specificity measure completeness” (Olson et al., 2015).

For the resistance gene characterization assay, accuracy, precision, sensitivity, and specificity could not be calculated for neither the core nor extended validation datasets for the database standard because no such information was available. They could be calculated for the ResFinder, CARD, and NDARO databases (with the exception of precision and sensitivity for the extended validation dataset for NDARO due to the lack of TP results) for both the core and extended validation datasets for the tool standard, and generally displayed very high performance (all values >99%), with the exception of precision for the CARD database that was lower (∼87.50%) due to the presence of one FP in all samples that most likely represents an artifact from the RGI tool (see the section “Results”). For the sequence typing assay, precision and specificity displayed perfect scores of 100% for the core validation dataset for the database standard. Accuracy (98.62%) and sensitivity (97.12%) were only slightly lower, and could be explained by the presence of some FN (i.e., the allele detected by the workflow does not match the database standard). These were, however, due to no alleles being present for some loci in the database standard (i.e., missing data) rather than real mismatches (Figure 3), implying that both accuracy and sensitivity are underestimated. Precision and specificity also displayed perfect scores of 100% for both the core and extended validation datasets for the tool standard. Accuracy and sensitivity were again slightly lower, but still attained very high values of 99.37 and 98.68%, and 99.78 and 99.52%, for the core and extended validation datasets, respectively. This was similarly explained by the presence of some FN (i.e., the allele detected by the workflow does not match the tool standard), which were in this case due to a different manner between the workflow and tool standard of handling loci for which there were multiple exact matches rather than real mismatches (Figure 4). Both the accuracy and sensitivity are hence again underestimated. Unexpectedly, accuracy and sensitivity were higher for the tool standard than the database standard for the core validation dataset because the reference tool, like our workflow, did manage to identify an allele for all loci for which an identifier was missing in the database standard. For the serogroup determination assay, precision and specificity displayed perfect scores of 100% for both the core and extended validation datasets for the database standard. Accuracy and sensitivity were lower, but still attained high values of 95.27 and 90.55%, and 96.09 and 92.19%, for the core and extended validation datasets, respectively. Similar to the sequence typing assay, these lower scores were caused by some FN (i.e., the detected serogroup does not match the database standard), which were, however, not due to missing data in the database standard but actual mismatches between the workflow and database standard. These mismatches could potentially be explained by the complexity of this assay that represents an algorithmic layer on top of the more “simple” sequence typing (see the section “Materials and Methods”), or alternatively inaccurate information in the database standard (see also below). Nevertheless, values for these performance metrics are in line with other reported results for *in silico* serotyping such as for instance witnessed in the comparison of commonly used *in silico* serotyping tools for *Salmonella* (Yachison et al., 2017). Accuracy, precision, sensitivity, and specificity all displayed perfect scores of 100% for both the core and extended validation datasets for the tool standard. Since the reference tool (i.e., the PubMLST sequence query tool) only presents results for the individual capsule loci, this indicates both our workflow and tool standard experience the same difficulties in predicting the serotype.

The following considerations should be taken into account for our validation strategy. First, all bioinformatics assays are at the level of the genotype, which does not necessarily correspond with the phenotype. It is for instance well documented that antibiotics resistance genes are not always expressed, and therefore not always result in the corresponding resistant phenotype (Didelot et al., 2012; Holmes et al., 2018; Rossen et al., 2018). The same has been witnessed for the serotype (Lindsey et al., 2016; Yachison et al., 2017). Only systematic and long-term monitoring of genotype versus phenotype relationships will allow to evaluate their concordance (Koser et al., 2012; Ellington et al., 2017), but in the genomic era, a paradigm shift should be envisaged where currently existing methods for pathogen typing based on the phenotype are phased out by pathogen characterization based on the genotype, despite issues for backward compatibility with traditional methods (Yachison et al., 2017). Second, some performance metrics could easily be misinterpreted or even manipulated in certain contexts. For instance, the resistance characterization assay for the ResFinder database had a perfect accuracy of 100% for both the core and extended validation datasets for the tool standard, but only very few TP existed because *N. meningitidis* typically only harbors a limited number of antibiotics resistance genes (Rouphael and Stephens, 2012). This implies that most database genes will never be detected but still contribute to the overall accuracy through an overwhelming number of TN compared to TP. Populating any database with irrelevant genes would therefore artificially increase the accuracy of any such assay, and only properly considering and evaluating other metrics such as precision and sensitivity can help to put this in its proper context (Hardwick et al., 2017). Third, evaluation by means of a database standard is preferred over a tool standard because the former contains high-quality genotypic information, but the current reality is that such databases are still scarce and/or incomplete. The database standard employed for *N*. *meningitidis* still contained some missing data, since both our workflow and the reference tool managed to identify several loci for which no identifier was stored in the database standard. Additionally, it always remains a possibility for any biobank that mutations are introduced over time due to micro-evolutionary events caused by freezing, thawing, and repeated cultivations of the reference collection leading to differences (Mellmann et al., 2017). This highlights that the construction of “gold standard” databases will greatly benefit the scientific community by providing a high-quality reference standard to which results of different bioinformatics methods and workflows can be compared against, for which notable efforts are already ongoing within the microbial community such as spearheaded by the Global Microbiological Identifier initiative, and which are expected to aid future validation efforts (Timme et al., 2017). Fourth, the current validation strategy is at the level of the isolate, and does not consider any phylogenomic analysis comprising many isolates. The validation of such a bioinformatics assay, however, represents an additional layer of complexity and constitutes an entire study on its own, first requiring consensus in the scientific community about how WGS workflows for isolates can be validated, to which our study represents a significant contribution.

Because access to the required bioinformatics expertise and/or resources remain obstacles for many NRCs and other laboratories from smaller and/or less developed countries (WHO, 2018), our bioinformatics workflow has been made available as a “push-button” pipeline (compatible with all data generated through the Illumina technology) accessible free-of-charge at the public Galaxy instance of our institute^8^ for non-profit and academic usage by this target audience. Nevertheless, properly evaluating performance through a validation strategy as described in this study is paramount when using this resource to ensure high-quality results are obtained on a set of samples that contain a representative (sub)set of genetic variation typically encountered in the population under investigation. Although both the larger volume and genetic diversity of samples expected to be analyzed by NRCs and other laboratories from larger and/or more developed countries implies that this resource will not scale well with their requirements, our “push-button” implementation can still be used by the latter as a showcase to demonstrate how the bioinformatics workflow was locally implemented and made available to specialists of the Belgian NRC *Neisseria*, since they employ the workflow similarly through an in-house instance of Galaxy (the volume of samples in Belgium is currently not high enough to motivate automated workflow execution nor does it present issues with scalability when using the Galaxy framework). A training video is also available as a tutorial for using this resource (see Supplementary Material).

## Conclusion

We reported a validation strategy focusing specifically on the bioinformatics analysis for clinical microbiological isolate WGS data, demonstrating generally high to very high performance, highlighting the added value and feasibility of employing WGS with the aim of being integrated into routine use under a quality system in an applied public health setting. Our validation strategy can be extended to other bioinformatics assays, WGS workflow components (e.g., library preparation, etc.), different WGS workflows and/or sequencing technologies, and pathogens. Several similar endeavors are currently being undertaken for other pathogens of interest for other Belgian NRCs and NRLs, and will in the future help to narrow the gap between the widely acclaimed success of WGS in research environments and its practical implementation in applied settings.

## Data Availability

The datasets supporting the conclusions of this study have been deposited in the NCBI SRA under accession number SRP137803 (in-house sequenced data), Zenodo (http://doi.org/10.5281/zenodo.1575931) (results of all bioinformatics analysis for both the core and extended validation datasets), and are included within this manuscript and its Supplementary Files (results of the validation for both the core and extended validation datasets of all bioinformatics assays).

## Author Contributions

KV, NR, and SD conceived and designed this study. KV supervised the project. SD supervised the data generation. BB constructed the bioinformatics workflow and performed all bioinformatics analysis. RW, QF, and JV contributed toward the algorithmic implementation of the bioinformatics workflow. P-JC, WM, and SB collected and isolated DNA of *N*. *meningitidis* samples to be used for the validation, and provided specialist feedback on the required functionalities of the bioinformatics workflow. BB and KV conceived the validation strategy, for which SD provided input and feedback. BB and KV analyzed the validation results, and wrote the draft for the manuscript. All authors aided in interpretation of the results and writing of the final manuscript.

## Conflict of Interest Statement

The authors declare that the research was conducted in the absence of any commercial or financial relationships that could be construed as a potential conflict of interest.

## Footnotes

1 http://www.genomicepidemiology.org

2 http://pubmlst.org

3 https://www.ncbi.nlm.nih.gov/pathogens/antimicrobial-resistance/

4 http://pubMLST.org

5 http://doi.org/10.5281/zenodo.1575931

6 https://cge.cbs.dtu.dk/services/ResFinder

7 https://doi.org/10.5281/zenodo.1452401

8 https://galaxy.sciensano.be
