# Supplementary material for: Validation of a Bioinformatics Workflow for Routine Analysis of Whole-Genome Sequencing Data and Related Challenges for Pathogen Typing in a European National Reference Center: Neisseria meningitidis as a Proof-of-Concept
Source: Front Microbiol. 2019 Mar 6;10:362. doi: 10.3389/fmicb.2019.00362 (PMC6414443; doi:10.3389/fmicb.2019.00362)
Supplement: Supplementary file 1 [file Data_Sheet_1.pdf]

Supplementary Material

Validation of a bioinformatics workflow for routine analysis of whole-genome sequencing data and related challenges for pathogen typing in a European National Reference Center: *Neisseria meningitidis* as a proof-of-concept

Bert Bogaerts[1], Raf Winand[1], Qiang Fu[1], Julien Van Braekel[1], Pieter-Jan Ceyssens[2], Wesley Mattheus[2], Sophie Bertrand[2], Sigrid C. J. De Keersmaecker[1], Nancy H. C. Roosens[1], and Kevin Vanneste[1,*]

[1] Transversal activities in Applied Genomics, Sciensano, Brussels, Belgium

[2] Bacterial Diseases, Sciensano, Brussels, Belgium

*** Correspondence:**
Kevin Vanneste

kevin.vanneste@sciensano.be

Table of Contents

1 Supporting information: general ... 4
1.1 Figures ... 4
1.1.1 Figure S1 ... 4
1.1.2 Figure S2 ... 5
1.1.3 Figure S3 ... 6
1.2 Tables ... 7
1.2.1 Table S1 ... 7
1.2.2 Table S2 ... 8
1.2.3 Table S3 ... 9
2 Supporting information: core validation dataset ... 10
2.1 Figures ... 10
2.1.1 Figure S4 ... 10
2.1.2 Figure S5 ... 11
2.1.3 Figure S6 ... 11
2.2 Tables ... 13
2.2.1 Table S4 ... 13
2.2.2 Table S5 ... 15
2.2.3 Table S6 ... 19
2.2.4 Table S7 ... 25
2.2.5 Table S8 ... 29
2.2.6 Table S9 ... 67
2.2.7 Table S10 ... 69
2.2.8 Table S11 ... 74
3.1.1 Table S12 ... 79
3.1.2 Table S13 ... 81
3.1.3 Table S14 ... 86
3.1.4 Table S15 ... 88
3.1.5 Table S16 ... 91
3.1.6 Table S17 ... 93
3.1.7 Table S18 ... 95
3.1.8 Table S19 ... 97

4 Supporting information: extended validation dataset 99
4.1 Figures 99
4.1.1 Figure S7 99
4.1.2 Figure S8 100
4.1.3 Figure S9 101
4.2 Tables 102
4.2.1 Table S20 102
4.2.2 Table S21 104
4.2.3 Table S22 106
4.2.4 Table S23 108
4.2.5 Table S24 110
4.2.6 Table S25 122
4.2.7 Table S26 124
4.2.8 Table S27 126
4.2.9 Table S28 128
4.2.10 Table S29 130
4.2.11 Table S30 131
4.2.12 Table S31 131
5 References 133

1 Supporting information: general

1.1 Figures

1.1.1 Figure S1

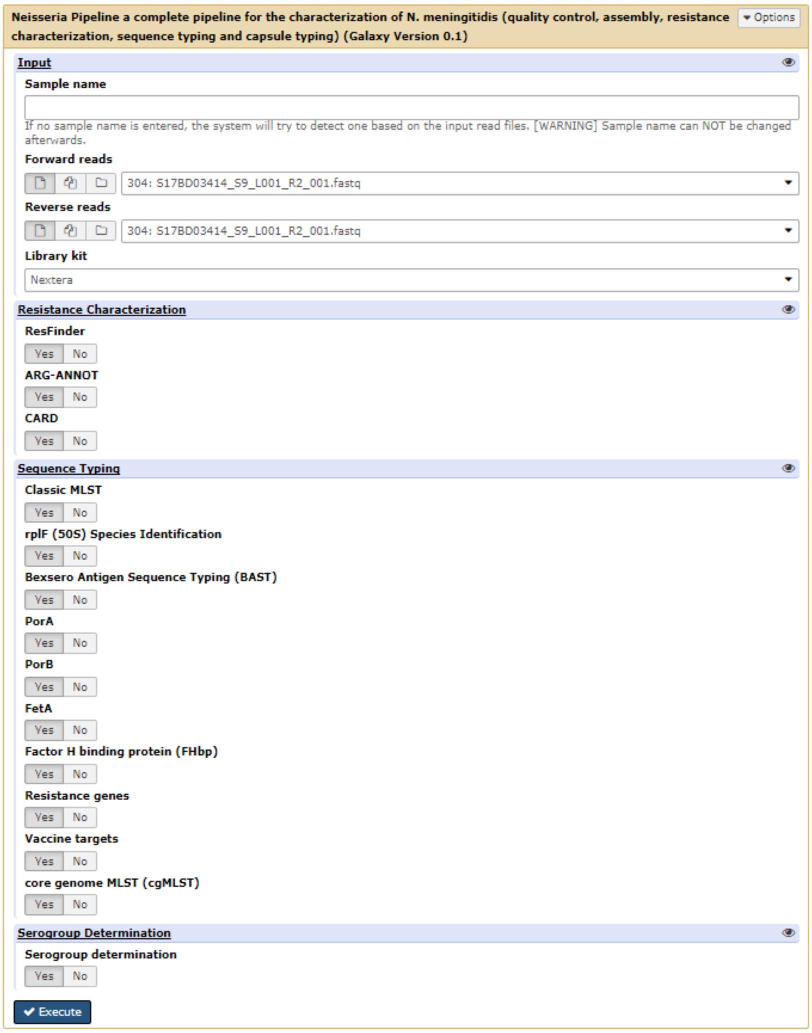

Figure S1: Pipeline interface in Galaxy. The interface is divided in four sections. In the ‘input’ section, the sample name, forward and reverse reads, and library kit used for data generation need to be selected. The three other sections, ‘resistance characterization’, ‘sequence typing’, and ‘serogroup determination’ each contain different analyses that can be either turned on or off (see Material and methods). By default, all analyses are put on, but users can turn of certain analyses.

1.1.2 Figure S2

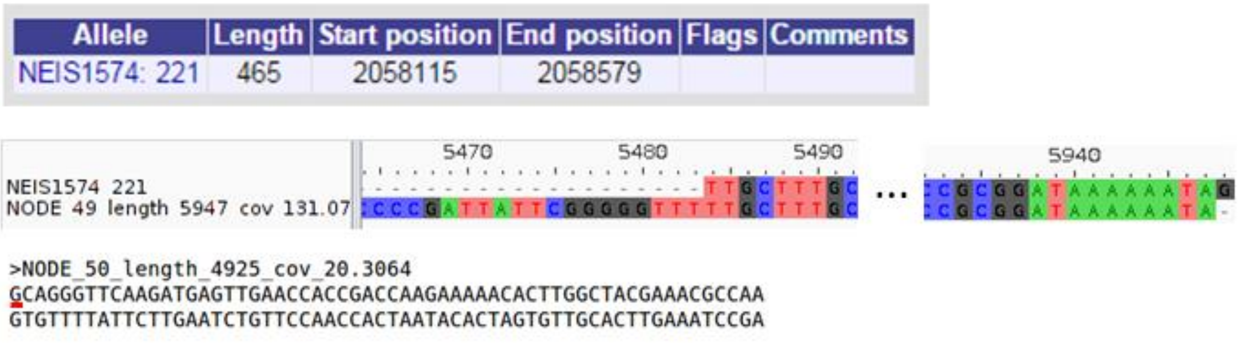

Figure S2: Example of a false positive hit being detected due to contig concatenation by the PubMLST sequence query tool. The sequence of sample ‘Z6429’ from run A was used as input for the reference tool, which finds a perfect hit (full length and 100% identity) for the locus NEIS1574 and reports the allele with identifier 221. A large part of this allele is present in the contig ‘NODE 49’ but the last base is missing so that the pipeline does not detect an allele. In contrast, the reference tool concatenates contigs before the alignment stage. By appending the contig ‘Node_50’ to the end of the contig ‘Node_49’, the complete NEIS1574 sequence is reconstructed, resulting in a perfect hit. This hit is most likely a false positive, as the ordering of contigs in the assembly is based on estimated coverage and does not represent any physical link.

1.1.3 Figure S3

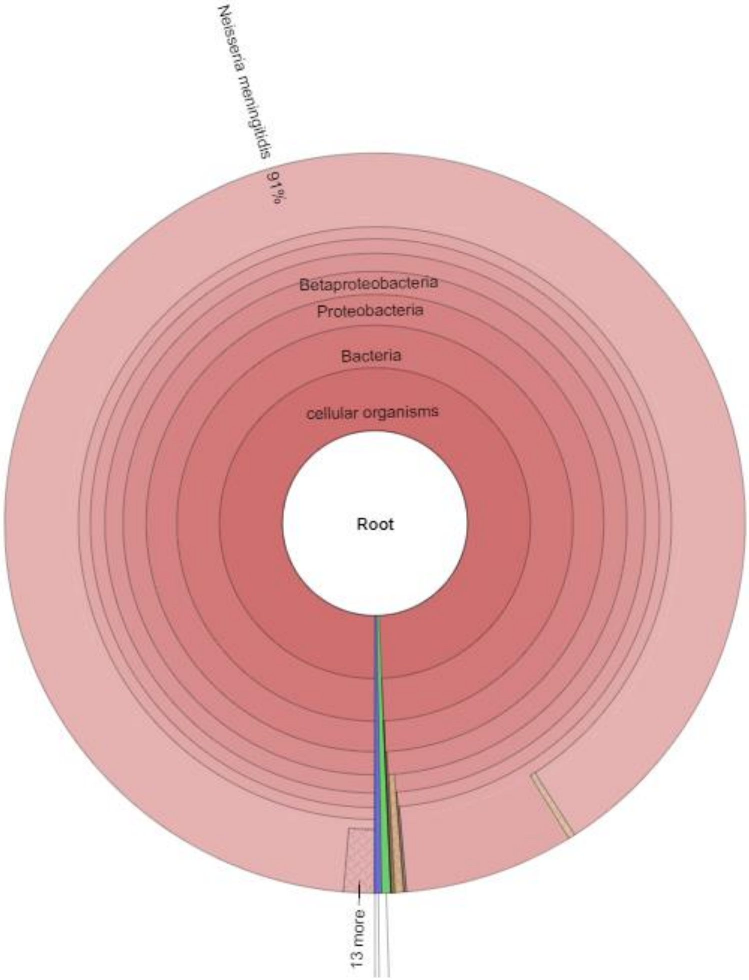

Figure S3: Visualization of the Kraken kmer-based taxonomic classification on the raw reads of sample Z4242 (run A). To investigate whether this sample could contain a contamination explaining its lower N50 and % of identified cgMLST loci, Kraken (Wood and Salzberg, 2014) was used against an in-house dump of the complete genomes present in the NCBI RefSeq Microbial Genomes database (O'Leary et al., 2016) (database retrieved 24/01/2018), and afterwards results were visualized with Krona (Ondov et al., 2011). No indications of large-scale contaminations were found.

1.2 Tables

1.2.1 Table S1

Table S1: Resistance characterization color codes used in the output report. Color codes for the different hit types are reported (see Material and methods).

Hit type	Subject length covered	% identity	Color
Perfect	100%	100%	
Imperfect identity	100%	90%-100%	
Imperfect short	60%-100%	90%-100%	

1.2.2 Table S2

Table S2: Sequence typing color codes used in the output report. Color codes for the different hit types are reported (see Material and methods).

Hit type	Subject length covered	% identity	Color
Perfect	100%	100%	
Imperfect identity	100%	90%-100%	
Imperfect short	60%-100%	90%-100%	
Multi-hit	60%-100%	90%-100%	
No hit	<60%	<90%	

1.2.3 Table S3

Table S3: Overview of the sources of the sequence typing schemas. All schemas are extracted from https://pubmlst.org (Bratcher et al., 2014; Jolley and Maiden, 2010). The first column refers to first column of Table 2 in the main manuscript, while the second and third columns contain the link to the schema information page from PubMLST and the links to one or more loci that are combined into a novel schema, respectively.

**Schema**	**URL**	**Loci**
Classic MLST	http://rest.pubmlst.org/db/pubmlst_neisseria_seqdef/schemes/1	/
rplF	http://rest.pubmlst.org/db/pubmlst_neisseria_seqdef/schemes/6	/
cgMLST	http://rest.pubmlst.org/db/pubmlst_neisseria_seqdef/schemes/47	/
Bexsero Antigen Sequence Typing	http://rest.pubmlst.org/db/pubmlst_neisseria_seqdef/schemes/53	/
porA	/	http://rest.pubmlst.org/db/pubmlst_neisseria_seqdef/loci/PorA_VR1<br>http://rest.pubmlst.org/db/pubmlst_neisseria_seqdef/loci/PorA_VR2
porB	/	http://rest.pubmlst.org/db/pubmlst_neisseria_seqdef/loci/NEIS2020
fetA	/	http://rest.pubmlst.org/db/pubmlst_neisseria_seqdef/loci/FetA_VR
fHbp	/	http://rest.pubmlst.org/db/pubmlst_neisseria_seqdef/loci/'fHbp<br>http://rest.pubmlst.org/db/pubmlst_neisseria_seqdef/loci/fHbp_PEPTIDEfrag_Pasteur<br>http://rest.pubmlst.org/db/pubmlst_neisseria_seqdef/loci/fHbp_DNAfrag_Pasteur<br>http://rest.pubmlst.org/db/pubmlst_neisseria_seqdef/loci/FHbp_segment_E<br>http://rest.pubmlst.org/db/pubmlst_neisseria_seqdef/loci/FHbp_segment_D<br>http://rest.pubmlst.org/db/pubmlst_neisseria_seqdef/loci/FHbp_segment_C<br>http://rest.pubmlst.org/db/pubmlst_neisseria_seqdef/loci/FHbp_segment_B<br>http://rest.pubmlst.org/db/pubmlst_neisseria_seqdef/loci/FHbp_segment_A<br>http://rest.pubmlst.org/db/pubmlst_neisseria_seqdef/loci/fHbp_peptide
Resistance genes	http://rest.pubmlst.org/db/pubmlst_neisseria_seqdef/schemes/5	/
Vaccine targets	/	http://rest.pubmlst.org/db/pubmlst_neisseria_seqdef/loci/NEIS2109<br>http://rest.pubmlst.org/db/pubmlst_neisseria_seqdef/loci/NEIS1969<br>http://rest.pubmlst.org/db/pubmlst_neisseria_seqdef/loci/'fHbp

2 Supporting information: core validation dataset

2.1 Figures

2.1.1 Figure S4

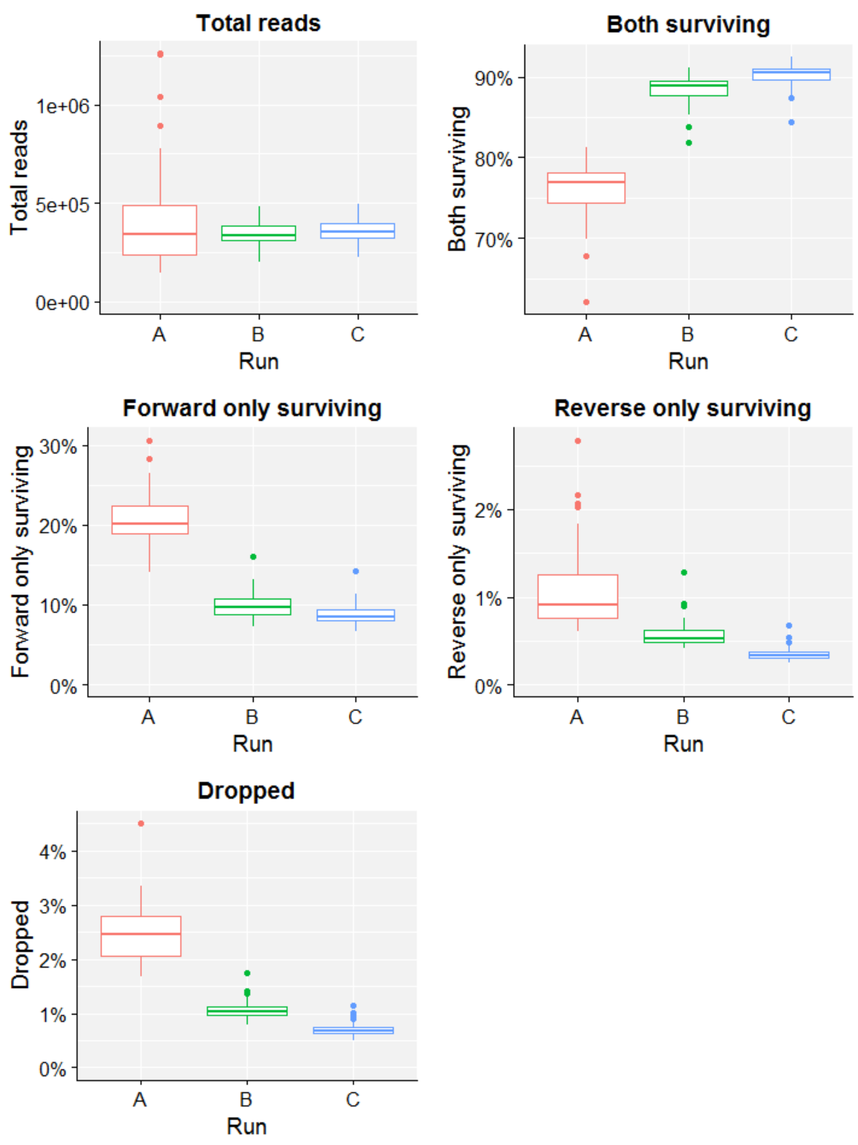

Figure S4: **Overview of read trimming statistics for the core validation dataset.** Simplified overview of the total number of reads and reads surviving trimming, as denoted by the title above each boxplot, for all samples and runs. The abscissa depicts the sequencing run (A, B or C), while the ordinate represents the number of reads (total reads), or the fraction of reads (percentage) that belong to the given category after trimming. Note that the ordinate for the graph depicting 'Both surviving' starts at 60% instead of 0% to enable illustrating the variation between runs more clearly. See also supplementary Table S5 for detailed values for all samples and runs.

2.1.2 Figure S5

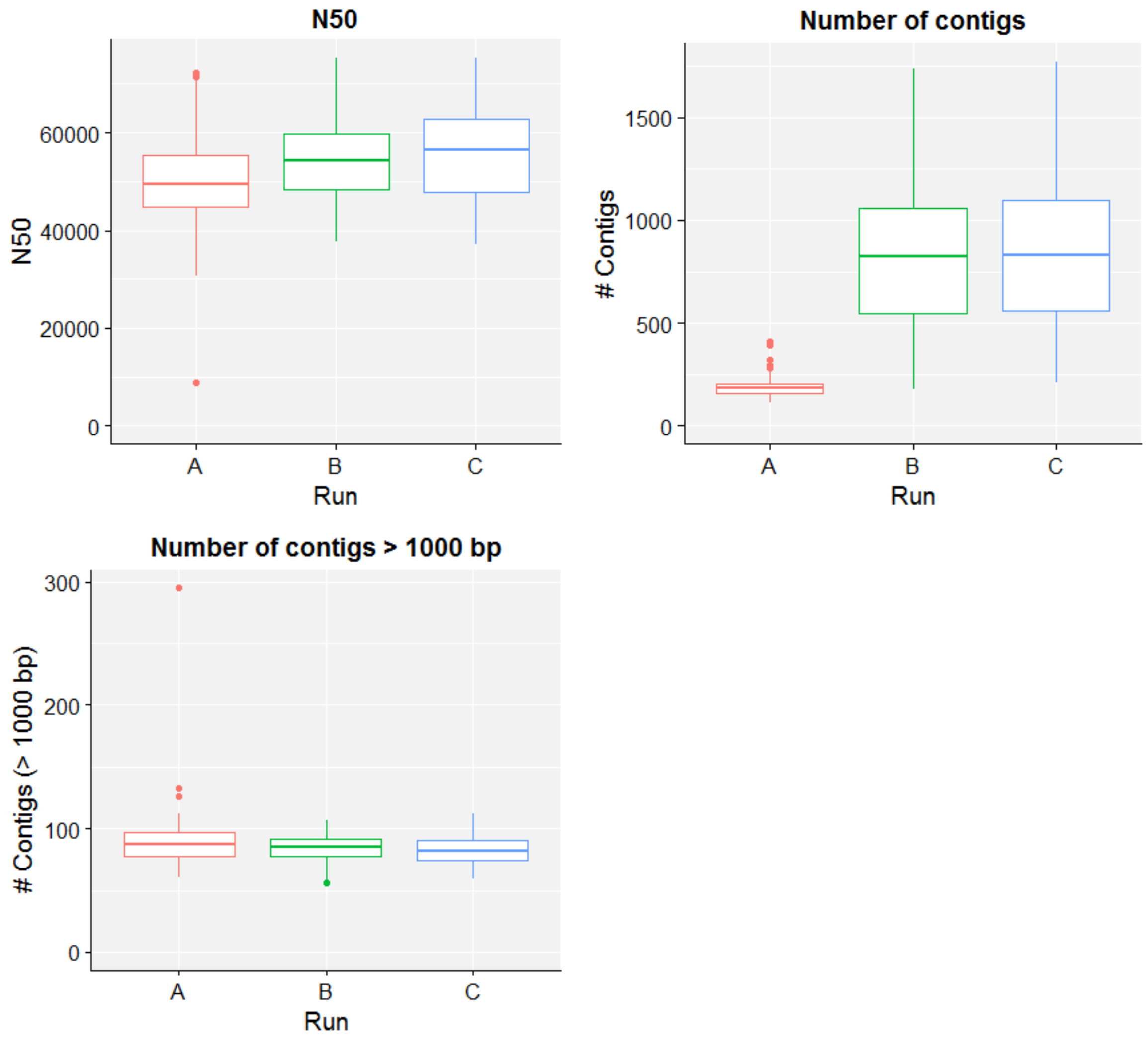

Figure S5: **Overview of assembly statistics for the core validation dataset.** The abscissa depicts the sequencing run (A, B or C), while the ordinate represents the value for the N50, total number of contigs and number of contigs larger than 1000 bases, as indicated by the title above each boxplot. See also supplementary Table S6 for detailed values for all samples and runs.

2.1.3 Figure S6

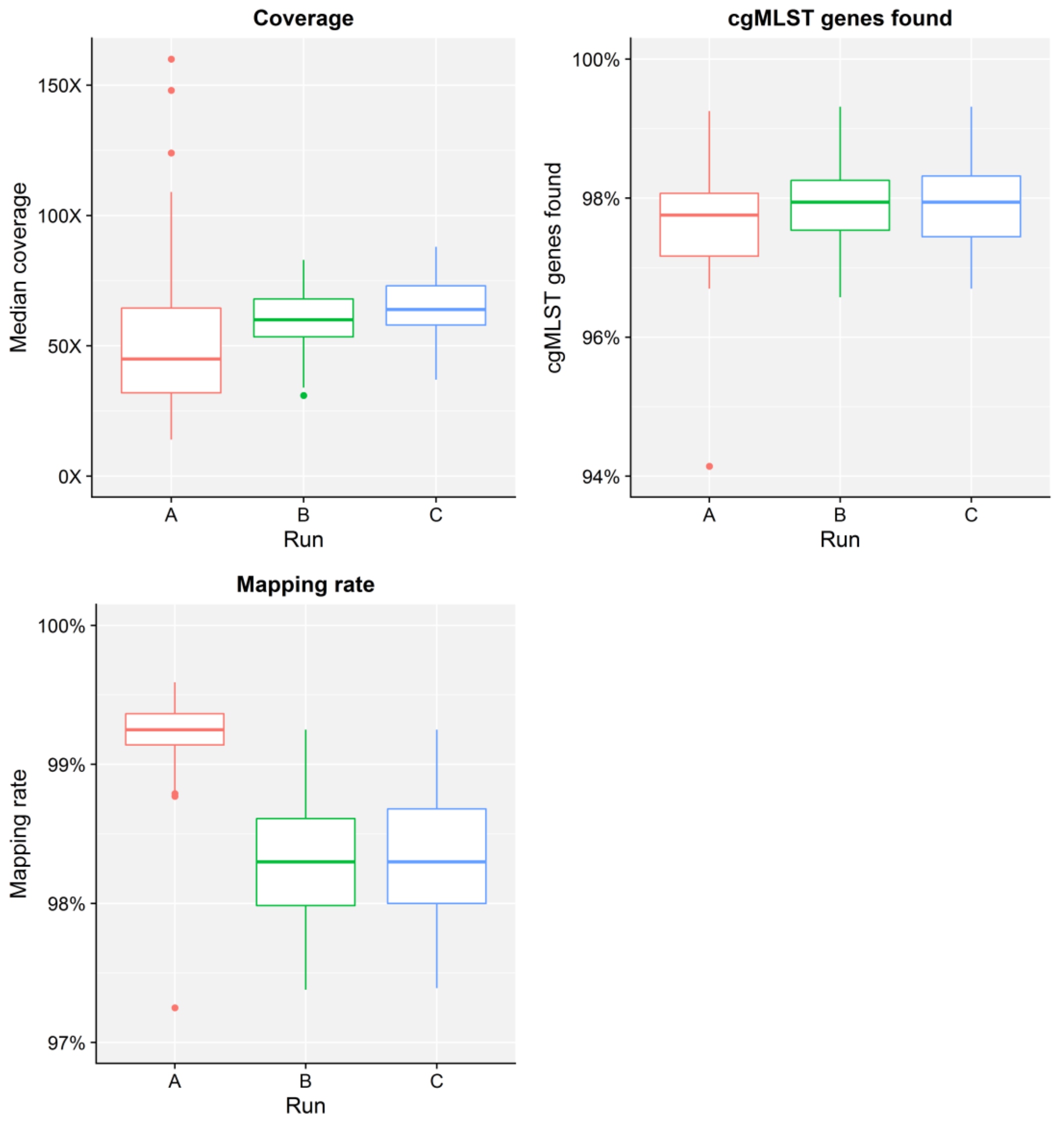

Figure S6: **Overview of advanced quality control statistics for the core validation dataset**. The abscissa depicts the run (A, B or C), while the ordinate represents the median coverage, percentage of cgMLST genes found and percentage of reads mapping back to the assembly, as indicated by the title above each boxplot. Note that the ordinate for the graphs depicting the percentage of cgMLST genes found and mapping rate, start at 94% and 97%, respectively, to enable illustrating the variation between runs more clearly. See also supplementary Table S7 for detailed values for all samples and runs.

2.2 Tables

2.2.1 Table S4

Table S4: List of 67 *N. meningitidis* samples selected from the 'gold standard' database maintained by the University of Oxford (Bratcher et al., 2014) for sequencing. Dashes are used to indicate missing or unknown information. The isolate is the main identifier of the sample, while the z-number is an alias that is used specifically for this collection.

Sample	Isolate	Country	Year	Disease	Epidemiology	Serogroup
Z1001	A4/M1027	USA	1937	invasive	epidemic	A
Z1035	120M	Pakistan	1967	meningitis and septicemia	endemic	A
Z1054	7891	Finland	1975	invasive	epidemic	A
Z1073	6748	Canada	1971	invasive	epidemic	A
Z1092	129E	Germany	1964	invasive	epidemic	A
Z1099	139M	Philippines	1968	-	endemic	A
Z1269	10	Burkina Faso	1963	invasive	endemic	A
Z1275	20	Niger	1963	invasive	-	A
Z1392	393	Greece	1968	carrier	epidemic	A
Z1439	254	Djibouti	1966	invasive	endemic	A
Z1466	S5611	Australia	1977	invasive	endemic	A
Z1534	CN100	UK	1941	invasive	epidemic	A
Z3842	H44/76	Norway	1976	invasive	epidemic	B
Z3906	154	China	1966	invasive	pandemic	A
Z4242	500	Italy	1984	-	endemic	C
Z4662	BZ 10	The Netherlands	1967	invasive	-	B
Z4664	BZ 83	The Netherlands	1984	invasive	-	B
Z4665	BZ 133	The Netherlands	1977	invasive	-	B
Z4667	BZ 147	The Netherlands	1963	invasive	-	B
Z4671	BZ 163	The Netherlands	1979	invasive	-	B
Z4672	BZ 169	The Netherlands	1985	invasive	-	B
Z4673	BZ 198	The Netherlands	1986	invasive	endemic	B
Z4674	BZ 232	The Netherlands	1964	invasive	-	B
Z4675	DK 24	Denmark	1940	invasive	-	B
Z4676	DK 353	Denmark	1962	invasive	-	B
Z4678	EG 327	Germany	1985	invasive	-	B
Z4681	EG 011	Germany	1986	invasive	-	B

Z4682	NG 3/88	Norway	1988	invasive	-	B
Z4683	NG 4/88	Norway	1988	invasive	-	B
Z4684	NG 6/88	Norway	1988	invasive	-	B
Z4685	NG F26	Norway	1988	carrier	-	B
Z4686	NG H15	Norway	1988	carrier	carrier	B
Z4687	NG H41	Norway	1988	carrier	-	B
Z4688	NG H38	Norway	1988	carrier	-	B
Z4689	NG E31	Norway	1988	carrier	-	B
Z4690	NG G40	Norway	1988	carrier	carrier	B
Z4691	NG E28	Norway	1988	carrier	carrier	B
Z4692	NG E30	Norway	1988	carrier	carrier	B
Z4693	NG H36	Norway	1988	carrier	carrier	B
Z4707	297-0	Chile	1987	carrier	carrier	B
Z4708	3906	China	1977	invasive	-	A
Z4709	SWZ107	Switzerland	1986	invasive	-	C
Z4710	528	Russia	1989	invasive	endemic	A
Z4711	1000	Russia	1988	invasive	endemic	A
Z4756	371	India	1980	invasive	-	A
Z4765	BRAZ10	Brazil	1976	-	-	A
Z5005	106	Morocco	1967	invasive	epidemic	A
Z5035	79128	China	1979	invasive	epidemic	C
Z5037	322/85	Germany	1985	invasive	endemic	C
Z5043	79126	China	1979	invasive	epidemic	B
Z5826	92001	China	1992	invasive	epidemic	B
Z6412	B6116/77	Iceland	1977	invasive	-	B
Z6414	94/155	New Zealand	1994	invasive	-	B
Z6415	312 901	UK	1996	invasive	-	B
Z6416	AK22	Greece	1992	invasive	-	Z
Z6418	204/92	Cuba	1992	invasive	-	X
Z6419	400	Austria	1991	invasive	-	X
Z6422	50/94	Norway	1994	invasive	-	W
Z6426	91/40	New Zealand	1991	meningitis	epidemic	Y
Z6427	88/03415	UK	1988	invasive	-	Y
Z6428	E32	Norway	1988	carrier	-	Z
Z6429	E26	Norway	1988	carrier	-	B
Z6430	860060	The Netherlands	1986	invasive	-	B
Z6431	890326	The Netherlands	1989	invasive	-	B
Z6432	A22	Norway	1986	carrier	-	B
Z6433	71/94	Norway	1994	invasive	-	B
Z6434	860800	The Netherlands	1986	invasive	-	B

2.2.2 Table S5

Table S5: Overview of read trimming statistics for the core validation dataset. The first, second and third columns list the sample name, the run name and the total number of reads, respectively. The fourth, fifth, sixth and seventh columns list the fraction of total reads where both members of the read pair passed trimming, the fraction of total reads where only the forward read passed read trimming, the fraction of total reads where only the reverse read passed read trimming, and the fraction of total reads where both read pair members were dropped in the trimming step. See also supplementary Figure S4 for a simplified overview.

Sample	Run	Total reads	Both reads surviving (%)	Forward read only surviving (%)	Reverse read only surviving (%)	Both reads dropped (%)
Z1001	A	699061	80.32	16.66	1.23	1.79
Z1035	A	493025	77.64	19.75	0.93	1.69
Z1054	A	894572	79.18	17.77	1.21	1.84
Z1073	A	478636	77.83	18.86	1.26	2.05
Z1092	A	1254870	77.64	19.05	1.46	1.85
Z1099	A	435692	79.85	17.28	1.16	1.70
Z1269	A	201201	77.60	19.11	0.90	2.39
Z1275	A	301448	78.12	19.05	0.81	2.02
Z1392	A	384778	74.86	21.63	1.43	2.08
Z1439	A	749695	77.10	20.26	0.86	1.77
Z1466	A	566147	77.27	19.92	0.98	1.83
Z1534	A	482939	76.53	20.71	0.89	1.86
Z3842	A	744569	77.52	19.05	1.46	1.97
Z3906	A	316309	71.29	24.21	2.03	2.47
Z4242	A	157170	62.11	30.60	2.79	4.50
Z4662	A	436277	79.30	17.55	1.16	1.98
Z4664	A	392927	78.92	17.19	1.84	2.05
Z4665	A	238240	77.28	19.60	0.76	2.36
Z4667	A	373726	73.12	24.19	0.68	2.01
Z4671	A	664047	76.12	20.13	1.62	2.14
Z4672	A	1042703	74.65	22.48	0.91	1.96
Z4673	A	323000	80.07	15.86	1.81	2.26
Z4674	A	313554	76.58	19.11	1.83	2.48
Z4675	A	461497	73.22	23.66	1.00	2.12
Z4676	A	503094	76.91	20.42	0.89	1.78
Z4678	A	1259430	80.35	15.87	1.59	2.19
Z4681	A	757866	77.81	19.37	0.92	1.90
Z4682	A	685406	78.18	18.82	1.07	1.93
Z4683	A	322553	75.17	21.51	0.80	2.52
Z4684	A	453686	79.33	16.91	1.70	2.05
Z4685	A	346188	78.49	18.18	0.94	2.39
Z4686	A	171087	77.44	17.26	2.06	3.24
Z4687	A	446491	77.49	19.36	0.79	2.37
Z4688	A	155111	73.63	22.78	0.73	2.86
Z4689	A	239594	78.10	18.38	0.96	2.56

Z4690	A	145140	67.81	28.27	0.61	3.31
Z4691	A	254533	75.72	20.96	0.81	2.50
Z4692	A	308538	76.95	19.22	1.14	2.69
Z4693	A	314610	71.62	24.67	0.72	3.00
Z4707	A	173090	81.25	14.10	2.17	2.48
Z4708	A	262257	78.36	18.08	1.01	2.56
Z4709	A	196192	74.98	20.87	0.99	3.15
Z4710	A	246301	76.97	19.33	1.01	2.69
Z4711	A	419474	74.37	22.07	0.75	2.81
Z4756	A	226083	78.64	17.98	0.97	2.41
Z4765	A	222856	71.88	24.74	0.62	2.75
Z5005	A	208429	73.71	22.49	0.87	2.92
Z5035	A	341547	69.83	26.14	0.76	3.27
Z5037	A	175969	76.32	19.26	1.34	3.08
Z5043	A	409462	77.19	19.46	0.88	2.47
Z5826	A	235283	75.71	21.06	0.77	2.46
Z6412	A	528544	75.12	21.36	0.81	2.71
Z6414	A	252328	76.28	20.45	0.75	2.51
Z6415	A	376855	76.21	20.19	0.83	2.77
Z6416	A	216578	71.81	24.61	0.63	2.95
Z6418	A	244544	71.45	24.68	0.75	3.12
Z6419	A	220587	72.53	23.73	0.76	2.98
Z6422	A	250692	80.75	14.96	1.79	2.50
Z6426	A	685451	74.49	21.79	0.80	2.92
Z6427	A	215487	69.90	26.07	0.68	3.34
Z6428	A	216304	76.05	20.57	0.82	2.56
Z6429	A	342917	77.99	19.14	0.69	2.18
Z6430	A	233852	74.20	22.29	0.72	2.79
Z6431	A	775591	79.74	16.36	1.46	2.44
Z6432	A	219316	69.96	26.48	0.61	2.95
Z6433	A	510523	76.96	20.08	0.74	2.22
Z6434	A	458350	74.58	22.51	0.68	2.23
Z1001	B	353703	90.57	7.93	0.56	0.93
Z1035	B	400005	89.22	9.36	0.50	0.92
Z1054	B	235512	85.32	12.78	0.50	1.39
Z1073	B	440658	89.22	9.24	0.55	0.99
Z1092	B	396285	90.36	8.03	0.68	0.93
Z1099	B	403540	90.24	8.37	0.52	0.86
Z1269	B	303275	88.50	9.77	0.59	1.14
Z1275	B	362638	89.33	9.12	0.53	1.02
Z1392	B	301913	89.09	9.05	0.74	1.12
Z1439	B	330409	88.03	10.43	0.46	1.08
Z1466	B	226092	85.60	12.70	0.46	1.23
Z1534	B	334125	87.78	10.65	0.48	1.10
Z3842	B	303504	89.62	8.71	0.68	0.99
Z3906	B	291972	89.28	8.74	0.91	1.08
Z4242	B	200019	83.78	13.19	1.28	1.74
Z4662	B	243314	86.49	11.66	0.52	1.33

Z4664	B	360221	90.37	8.01	0.74	0.88
Z4665	B	338107	88.79	9.67	0.49	1.05
Z4667	B	360702	87.73	10.72	0.45	1.10
Z4671	B	359097	88.94	9.36	0.66	1.04
Z4672	B	386418	87.95	10.67	0.43	0.95
Z4673	B	361414	91.20	7.26	0.76	0.79
Z4674	B	394707	88.48	9.77	0.71	1.05
Z4675	B	305816	88.74	9.59	0.60	1.07
Z4676	B	389711	90.09	8.50	0.53	0.88
Z4678	B	343416	90.37	8.00	0.68	0.95
Z4681	B	471725	88.96	9.60	0.49	0.95
Z4682	B	387812	88.86	9.65	0.50	0.99
Z4683	B	374192	88.65	9.87	0.47	1.01
Z4684	B	311284	89.86	8.36	0.75	1.03
Z4685	B	344288	89.38	9.06	0.56	1.00
Z4686	B	290471	89.90	8.14	0.92	1.04
Z4687	B	212908	81.80	16.00	0.46	1.74
Z4688	B	231149	85.39	12.68	0.52	1.41
Z4689	B	337214	90.04	8.39	0.59	0.98
Z4690	B	379775	88.28	10.20	0.50	1.02
Z4691	B	251170	86.16	11.89	0.54	1.41
Z4692	B	392329	89.09	9.33	0.61	0.97
Z4693	B	358223	87.63	10.85	0.48	1.04
Z4707	B	337014	90.22	7.94	0.89	0.95
Z4708	B	326497	89.68	8.77	0.61	0.95
Z4709	B	252760	86.55	11.52	0.55	1.39
Z4710	B	317333	89.70	8.68	0.67	0.95
Z4711	B	418082	89.47	9.09	0.50	0.93
Z4756	B	314569	88.45	9.90	0.56	1.09
Z4765	B	287304	87.11	11.31	0.42	1.17
Z5005	B	278787	85.30	12.88	0.46	1.36
Z5035	B	322014	86.48	11.91	0.46	1.15
Z5037	B	314534	87.66	10.46	0.64	1.25
Z5043	B	396361	88.83	9.64	0.53	1.01
Z5826	B	311892	88.54	9.83	0.54	1.08
Z6412	B	316378	87.72	10.68	0.48	1.11
Z6414	B	457306	90.28	8.36	0.50	0.86
Z6415	B	250751	86.59	11.52	0.55	1.34
Z6416	B	480354	89.31	9.31	0.47	0.91
Z6418	B	417322	89.45	9.13	0.51	0.90
Z6419	B	313547	88.18	10.26	0.49	1.07
Z6422	B	389073	90.25	8.02	0.76	0.97
Z6426	B	380557	89.57	8.91	0.54	0.98
Z6427	B	355039	88.73	9.80	0.47	1.00
Z6428	B	329041	89.10	9.24	0.59	1.08
Z6429	B	383680	89.44	9.11	0.47	0.98
Z6430	B	352212	88.53	9.87	0.50	1.10
Z6431	B	410489	89.33	8.98	0.66	1.04

Z6432	B	372747	87.89	10.56	0.45	1.10
Z6433	B	321435	87.49	10.90	0.45	1.16
Z6434	B	321862	86.95	11.48	0.42	1.15
Z1001	C	366005	91.92	7.13	0.36	0.60
Z1035	C	412030	90.70	8.35	0.32	0.63
Z1054	C	245143	87.39	11.39	0.31	0.90
Z1073	C	454396	90.63	8.38	0.34	0.65
Z1092	C	413413	91.76	7.23	0.39	0.61
Z1099	C	415053	91.55	7.51	0.35	0.59
Z1269	C	314600	90.34	8.51	0.38	0.77
Z1275	C	371123	90.89	8.11	0.34	0.66
Z1392	C	314644	90.79	8.04	0.43	0.75
Z1439	C	342798	89.75	9.23	0.29	0.73
Z1466	C	234728	87.70	11.14	0.31	0.86
Z1534	C	345834	89.58	9.43	0.28	0.71
Z3842	C	315759	91.09	7.85	0.38	0.69
Z3906	C	309442	91.03	7.78	0.49	0.70
Z4242	C	225545	88.50	9.88	0.68	0.95
Z4662	C	252809	88.70	10.17	0.31	0.82
Z4664	C	376352	91.59	7.43	0.42	0.56
Z4665	C	349737	90.53	8.49	0.32	0.67
Z4667	C	371611	89.59	9.44	0.27	0.69
Z4671	C	374563	90.61	8.35	0.37	0.66
Z4672	C	399743	89.55	9.56	0.28	0.61
Z4673	C	376970	92.45	6.61	0.43	0.51
Z4674	C	415949	90.31	8.63	0.39	0.66
Z4675	C	317577	90.49	8.40	0.40	0.71
Z4676	C	402209	91.33	7.72	0.38	0.57
Z4678	C	356584	91.64	7.33	0.41	0.62
Z4681	C	491184	90.41	8.67	0.30	0.62
Z4682	C	402043	90.38	8.65	0.32	0.65
Z4683	C	388773	90.18	8.85	0.32	0.65
Z4684	C	326324	91.12	7.75	0.43	0.70
Z4685	C	355909	90.86	8.12	0.34	0.67
Z4686	C	306704	91.58	7.20	0.54	0.68
Z4687	C	222817	84.44	14.17	0.26	1.14
Z4688	C	240496	87.64	11.05	0.29	1.01
Z4689	C	349768	91.34	7.67	0.37	0.63
Z4690	C	391986	89.96	9.06	0.32	0.66
Z4691	C	260477	88.46	10.33	0.33	0.88
Z4692	C	410081	90.52	8.48	0.38	0.63
Z4693	C	371631	89.69	9.32	0.31	0.69
Z4707	C	354874	91.44	7.45	0.48	0.63
Z4708	C	339180	91.09	7.91	0.36	0.63
Z4709	C	264370	88.84	9.96	0.32	0.88
Z4710	C	330849	91.15	7.74	0.45	0.66
Z4711	C	431169	91.04	8.00	0.34	0.62
Z4756	C	324215	90.30	8.64	0.34	0.72

Z4765	C	294914	89.08	9.89	0.27	0.75
Z5005	C	291156	87.84	10.96	0.28	0.93
Z5035	C	333431	88.65	10.30	0.29	0.76
Z5037	C	328769	89.64	9.16	0.39	0.82
Z5043	C	408568	90.49	8.49	0.34	0.68
Z5826	C	323254	90.45	8.50	0.36	0.69
Z6412	C	329877	89.69	9.30	0.32	0.69
Z6414	C	470328	91.48	7.61	0.33	0.58
Z6415	C	259361	88.64	10.13	0.33	0.90
Z6416	C	494430	90.51	8.57	0.32	0.60
Z6418	C	428945	90.75	8.32	0.33	0.60
Z6419	C	323506	89.82	9.17	0.30	0.71
Z6422	C	405822	91.65	7.29	0.43	0.62
Z6426	C	391518	90.98	8.02	0.32	0.68
Z6427	C	366796	90.38	8.65	0.32	0.66
Z6428	C	342790	90.70	8.21	0.38	0.71
Z6429	C	393893	91.03	8.01	0.31	0.66
Z6430	C	362409	90.49	8.44	0.33	0.74
Z6431	C	426184	90.86	8.07	0.39	0.68
Z6432	C	384708	89.90	9.09	0.30	0.70
Z6433	C	330912	89.42	9.52	0.30	0.77
Z6434	C	332535	89.06	9.94	0.27	0.73

2.2.3 Table S6

Table S6: Overview of assembly statistics for the core validation dataset. The first and second columns list the sample and run name, respectively. The third, fourth and fifth columns list the N50, number of contigs, and number of contigs > 1,000 bases, respectively. See also supplementary Figure S5 for a simplified overview.

**Sample**	**Run**	**N50**	**Number of contigs**	**Number of contigs > 1000 bases**
Z1001	A	62847	168	66
Z1035	A	50995	174	83
Z1054	A	56559	297	74
Z1073	A	49858	192	80
Z1092	A	46985	411	78
Z1099	A	40309	195	94
Z1269	A	51902	115	75
Z1275	A	56155	127	67
Z1392	A	44863	203	91
Z1439	A	53091	158	81
Z1466	A	42331	186	91
Z1534	A	54829	149	78
Z3842	A	50373	161	86
Z3906	A	44864	139	82

Z4242	A	8743	392	295
Z4662	A	49050	203	94
Z4664	A	51120	183	88
Z4665	A	70990	144	76
Z4667	A	50562	289	95
Z4671	A	61106	160	77
Z4672	A	50118	227	97
Z4673	A	55500	184	87
Z4674	A	41875	204	102
Z4675	A	47325	253	111
Z4676	A	45494	279	86
Z4678	A	63158	249	95
Z4681	A	40363	238	112
Z4682	A	46112	182	104
Z4683	A	39198	190	98
Z4684	A	64829	215	78
Z4685	A	66486	154	75
Z4686	A	46786	152	100
Z4687	A	62202	185	85
Z4688	A	32366	291	126
Z4689	A	46118	260	98
Z4690	A	30508	317	132
Z4691	A	44007	183	99
Z4692	A	46109	139	81
Z4693	A	72315	182	81
Z4707	A	31407	179	101
Z4708	A	56593	161	78
Z4709	A	49389	156	90
Z4710	A	46063	200	108
Z4711	A	52456	201	97
Z4756	A	42750	206	87
Z4765	A	51136	149	81
Z5005	A	55419	154	82
Z5035	A	57251	168	72
Z5037	A	39704	186	105
Z5043	A	71560	153	68
Z5826	A	44765	126	75
Z6412	A	70999	128	60
Z6414	A	45109	149	76
Z6415	A	58278	131	69
Z6416	A	46639	147	88
Z6418	A	50929	162	89
Z6419	A	46382	157	90
Z6422	A	55365	158	93

Z6426	A	60276	163	74
Z6427	A	43798	134	86
Z6428	A	45679	202	95
Z6429	A	50951	175	63
Z6430	A	39785	180	106
Z6431	A	52838	246	83
Z6432	A	35406	197	112
Z6433	A	35514	258	90
Z6434	A	47116	184	90
Z1001	B	66648	381	65
Z1035	B	56636	1080	82
Z1054	B	49964	362	74
Z1073	B	54721	1194	92
Z1092	B	56042	879	81
Z1099	B	51941	890	79
Z1269	B	75270	378	56
Z1275	B	66599	440	68
Z1392	B	55372	689	84
Z1439	B	57879	384	78
Z1466	B	59431	465	79
Z1534	B	63545	564	75
Z3842	B	58646	534	85
Z3906	B	68338	457	64
Z4242	B	52033	175	83
Z4662	B	70504	468	71
Z4664	B	48014	616	87
Z4665	B	55927	729	78
Z4667	B	55077	588	82
Z4671	B	54313	724	70
Z4672	B	52276	513	87
Z4673	B	60493	444	86
Z4674	B	48782	879	95
Z4675	B	47106	566	89
Z4676	B	49703	567	79
Z4678	B	62199	776	85
Z4681	B	40442	867	97
Z4682	B	49638	809	92
Z4683	B	42814	967	97
Z4684	B	44792	1245	85
Z4685	B	64306	846	84
Z4686	B	63614	221	78
Z4687	B	44783	461	88
Z4688	B	40867	530	93
Z4689	B	46735	1060	95

Z4690	B	37830	1736	107
Z4691	B	43680	682	88
Z4692	B	54223	854	77
Z4693	B	65620	828	79
Z4707	B	49694	1041	89
Z4708	B	68942	1055	65
Z4709	B	52341	619	76
Z4710	B	55341	992	97
Z4711	B	49917	926	102
Z4756	B	55950	865	86
Z4765	B	60367	822	72
Z5005	B	53981	821	78
Z5035	B	67910	356	58
Z5037	B	45963	995	94
Z5043	B	48168	1513	88
Z5826	B	56104	1308	80
Z6412	B	53455	1101	69
Z6414	B	48620	1511	86
Z6415	B	58861	298	68
Z6416	B	45117	1514	98
Z6418	B	58153	1171	86
Z6419	B	63846	1036	85
Z6422	B	53649	1407	93
Z6426	B	60276	1179	79
Z6427	B	70157	762	78
Z6428	B	51990	650	92
Z6429	B	59465	1305	71
Z6430	B	49254	1295	98
Z6431	B	40214	1401	91
Z6432	B	39493	766	106
Z6433	B	39535	831	88
Z6434	B	38134	1176	97
Z1001	C	66329	382	64
Z1035	C	72550	1084	77
Z1054	C	59715	364	65
Z1073	C	60482	1299	90
Z1092	C	56042	919	77
Z1099	C	51847	918	82
Z1269	C	70795	349	60
Z1275	C	72538	414	60
Z1392	C	51843	753	83
Z1439	C	62327	364	76
Z1466	C	60918	507	73
Z1534	C	72640	608	70

Z3842	C	48309	583	85
Z3906	C	75331	507	65
Z4242	C	58828	212	74
Z4662	C	75130	506	62
Z4664	C	49517	604	81
Z4665	C	50077	720	78
Z4667	C	56526	563	79
Z4671	C	75207	764	67
Z4672	C	56818	536	84
Z4673	C	60492	462	81
Z4674	C	40467	902	98
Z4675	C	62510	551	76
Z4676	C	49451	589	80
Z4678	C	51439	817	90
Z4681	C	38247	854	102
Z4682	C	47048	799	98
Z4683	C	42808	951	102
Z4684	C	66986	1217	70
Z4685	C	46878	880	77
Z4686	C	64718	232	78
Z4687	C	46231	464	89
Z4688	C	39677	520	93
Z4689	C	53394	1029	91
Z4690	C	46109	1771	112
Z4691	C	47379	688	87
Z4692	C	53978	939	82
Z4693	C	65620	919	82
Z4707	C	54156	1113	83
Z4708	C	62947	1155	75
Z4709	C	61055	590	69
Z4710	C	53578	1035	101
Z4711	C	45359	961	108
Z4756	C	55950	892	83
Z4765	C	63947	809	72
Z5005	C	56949	824	82
Z5035	C	74824	330	59
Z5037	C	45963	1048	98
Z5043	C	49092	1498	96
Z5826	C	75033	1392	84
Z6412	C	75255	1121	63
Z6414	C	44180	1500	76
Z6415	C	60557	333	67
Z6416	C	49272	1575	103
Z6418	C	49739	1204	87

Z6419	C	60979	991	84
Z6422	C	56997	1414	84
Z6426	C	63698	1172	79
Z6427	C	60556	832	74
Z6428	C	58800	656	84
Z6429	C	58519	1394	70
Z6430	C	45148	1335	109
Z6431	C	37279	1485	97
Z6432	C	38501	808	98
Z6433	C	40451	887	82
Z6434	C	38740	1211	100

2.2.4 Table S7

Table S7: Overview of advanced quality control statistics for the core validation dataset. The first and second columns list the sample and run name, respectively. The third, fourth and fifth columns list the percentage of reads mapping back to the assembly, median coverage and number of cgMLST genes detected (on a total of 1605 loci - see Table 2), respectively. See also supplementary Figure S6 for a simplified overview.

Sample	Run	Mapping rate (%)	Median coverage	# of cgMLST loci found
Z1001	A	99.4	91	1565
Z1035	A	99.22	63	1574
Z1054	A	98.96	109	1582
Z1073	A	99.22	58	1567
Z1092	A	99.14	160	1568
Z1099	A	99.14	55	1568
Z1269	A	99.46	29	1574
Z1275	A	99.4	45	1572
Z1392	A	99.14	52	1569
Z1439	A	99.53	95	1567
Z1466	A	99.31	70	1573
Z1534	A	99.46	59	1554
Z3842	A	99.59	93	1585
Z3906	A	99.35	41	1579
Z4242	A	97.25	14	1511
Z4662	A	99.09	53	1569
Z4664	A	99.11	54	1578
Z4665	A	99.41	33	1573
Z4667	A	98.83	51	1574
Z4671	A	99.46	81	1571
Z4672	A	99.38	124	1584
Z4673	A	99.13	45	1592
Z4674	A	99.16	40	1571
Z4675	A	99.17	58	1552
Z4676	A	99.17	73	1566
Z4678	A	99.45	148	1557
Z4681	A	99.39	90	1570
Z4682	A	99.54	83	1571
Z4683	A	99.28	45	1555
Z4684	A	99.04	65	1572
Z4685	A	99.41	50	1567
Z4686	A	98.77	21	1582
Z4687	A	99.38	61	1560
Z4688	A	99.31	20	1559
Z4689	A	98.79	32	1552

Z4690	A	98.98	17	1553
Z4691	A	99.37	34	1556
Z4692	A	99.19	43	1578
Z4693	A	98.81	40	1578
Z4707	A	99.06	25	1556
Z4708	A	99.24	37	1556
Z4709	A	99.13	26	1566
Z4710	A	99.36	33	1554
Z4711	A	99.36	53	1564
Z4756	A	99.06	32	1574
Z4765	A	99.23	30	1580
Z5005	A	99.23	27	1568
Z5035	A	98.96	43	1556
Z5037	A	99.17	22	1556
Z5043	A	99.28	56	1559
Z5826	A	99.21	33	1586
Z6412	A	99.47	74	1571
Z6414	A	99.32	36	1567
Z6415	A	99.47	53	1574
Z6416	A	99.32	29	1567
Z6418	A	99.25	30	1573
Z6419	A	99.29	29	1582
Z6422	A	99.24	35	1588
Z6426	A	99.36	91	1593
Z6427	A	99.25	27	1587
Z6428	A	99.14	29	1552
Z6429	A	99.25	51	1555
Z6430	A	99.35	32	1563
Z6431	A	99.22	109	1571
Z6432	A	99.3	27	1567
Z6433	A	99.15	74	1570
Z6434	A	99.27	64	1575
Z1001	B	99.12	70	1564
Z1035	B	98.14	69	1575
Z1054	B	98.5	40	1577
Z1073	B	98.32	78	1569
Z1092	B	98.4	72	1570
Z1099	B	98.3	74	1572
Z1269	B	98.96	56	1576
Z1275	B	99.04	69	1571
Z1392	B	98.33	54	1566
Z1439	B	99.02	63	1566
Z1466	B	98.19	39	1568
Z1534	B	98.77	60	1556

Z3842	B	98.73	56	1581
Z3906	B	98.54	53	1581
Z4242	B	98.99	31	1585
Z4662	B	98.53	43	1577
Z4664	B	98.59	64	1580
Z4665	B	98.57	61	1573
Z4667	B	98.83	64	1574
Z4671	B	98.6	64	1569
Z4672	B	98.82	69	1582
Z4673	B	99.01	65	1592
Z4674	B	98.35	67	1573
Z4675	B	98.8	53	1550
Z4676	B	98.76	72	1567
Z4678	B	98.45	60	1558
Z4681	B	98.62	82	1567
Z4682	B	98.5	69	1572
Z4683	B	98.32	66	1557
Z4684	B	97.76	54	1574
Z4685	B	98.37	63	1568
Z4686	B	99.25	53	1589
Z4687	B	97.77	34	1560
Z4688	B	98.08	39	1561
Z4689	B	98.05	55	1555
Z4690	B	97.38	58	1569
Z4691	B	98.08	42	1565
Z4692	B	98.26	70	1582
Z4693	B	98.2	61	1581
Z4707	B	97.85	60	1563
Z4708	B	97.89	57	1557
Z4709	B	98.12	44	1573
Z4710	B	97.97	53	1553
Z4711	B	98.34	72	1566
Z4756	B	98.18	55	1574
Z4765	B	98.03	51	1582
Z5005	B	97.76	47	1571
Z5035	B	99.1	59	1561
Z5037	B	97.8	49	1559
Z5043	B	97.8	66	1560
Z5826	B	97.55	53	1588
Z6412	B	97.66	54	1574
Z6414	B	98	81	1572
Z6415	B	98.98	47	1575
Z6416	B	98.05	83	1571
Z6418	B	97.84	70	1579

Z6419	B	97.93	54	1587
Z6422	B	97.85	65	1587
Z6426	B	98.11	66	1594
Z6427	B	98.3	63	1591
Z6428	B	98.67	58	1555
Z6429	B	98.01	70	1558
Z6430	B	97.84	59	1570
Z6431	B	97.93	70	1570
Z6432	B	98.56	65	1576
Z6433	B	98.11	58	1572
Z6434	B	97.71	55	1575
Z1001	C	99.17	74	1564
Z1035	C	98.2	73	1576
Z1054	C	98.43	43	1579
Z1073	C	98.3	82	1571
Z1092	C	98.51	78	1570
Z1099	C	98.39	78	1572
Z1269	C	98.95	60	1579
Z1275	C	99.08	73	1570
Z1392	C	98.44	58	1569
Z1439	C	99.06	67	1564
Z1466	C	98.27	42	1571
Z1534	C	98.84	64	1555
Z3842	C	98.75	60	1584
Z3906	C	98.72	59	1581
Z4242	C	99.04	39	1585
Z4662	C	98.61	47	1573
Z4664	C	98.58	69	1579
Z4665	C	98.6	65	1573
Z4667	C	98.78	69	1575
Z4671	C	98.68	69	1569
Z4672	C	98.8	73	1583
Z4673	C	99.01	70	1594
Z4674	C	98.42	73	1573
Z4675	C	98.79	57	1552
Z4676	C	98.76	76	1565
Z4678	C	98.45	64	1558
Z4681	C	98.6	88	1568
Z4682	C	98.54	74	1567
Z4683	C	98.35	71	1557
Z4684	C	97.8	59	1572
Z4685	C	98.4	67	1567
Z4686	C	99.25	58	1589
Z4687	C	97.74	37	1561

Z4688	C	98.26	42	1564
Z4689	C	98.03	59	1556
Z4690	C	97.39	62	1569
Z4691	C	98.11	46	1564
Z4692	C	98.28	75	1581
Z4693	C	98.23	66	1581
Z4707	C	97.9	65	1564
Z4708	C	97.86	61	1559
Z4709	C	98.25	48	1576
Z4710	C	97.98	57	1552
Z4711	C	98.33	76	1566
Z4756	C	98.22	59	1576
Z4765	C	98.1	54	1582
Z5005	C	97.87	51	1572
Z5035	C	99.12	64	1560
Z5037	C	97.85	54	1563
Z5043	C	97.89	70	1561
Z5826	C	97.55	56	1587
Z6412	C	97.76	59	1574
Z6414	C	98.04	85	1571
Z6415	C	99.04	50	1575
Z6416	C	98.11	87	1572
Z6418	C	97.85	74	1577
Z6419	C	98	58	1588
Z6422	C	97.9	71	1590
Z6426	C	98.14	70	1594
Z6427	C	98.3	67	1590
Z6428	C	98.68	62	1554
Z6429	C	98	73	1558
Z6430	C	97.81	63	1567
Z6431	C	97.94	75	1572
Z6432	C	98.66	70	1576
Z6433	C	98.18	62	1572
Z6434	C	97.73	59	1575

2.2.5 Table S8

Table S8: Detected genes for the resistance gene characterization assay for the core validation dataset. The first, second and third columns list the sample name, database and run, respectively. The fourth column contains the name of the gene, while the fifth and sixth columns contain the corresponding percentage sequence identity and percentage of query sequence covered, respectively.

Sample	Database	Run	Gene	% Identity	% Query covered

Z1001	CARD	A	*farA*	100.00	100.00
Z1001	CARD	A	*farB*	97.77	100.00
Z1001	CARD	A	*macA*	96.78	100.00
Z1001	CARD	A	*macB*	97.26	100.00
Z1001	CARD	A	*mtrC*	97.26	100.00
Z1001	CARD	A	*mtrD*	99.22	100.00
Z1001	CARD	A	*mtrE*	93.74	99.93
Z1001	CARD	A	*mtrR*	97.63	100.00
Z1001	CARD	B	*farA*	100.00	100.00
Z1001	CARD	B	*farB*	97.77	100.00
Z1001	CARD	B	*macA*	96.78	100.00
Z1001	CARD	B	*macB*	97.26	100.00
Z1001	CARD	B	*mtrC*	97.26	100.00
Z1001	CARD	B	*mtrD*	99.22	100.00
Z1001	CARD	B	*mtrE*	93.74	99.93
Z1001	CARD	B	*mtrR*	97.63	100.00
Z1001	CARD	C	*farA*	100.00	100.00
Z1001	CARD	C	*farB*	97.77	100.00
Z1001	CARD	C	*macA*	96.78	100.00
Z1001	CARD	C	*macB*	97.26	100.00
Z1001	CARD	C	*mtrC*	97.26	100.00
Z1001	CARD	C	*mtrD*	99.22	100.00
Z1001	CARD	C	*mtrE*	93.74	99.93
Z1001	CARD	C	*mtrR*	97.63	100.00
Z1035	CARD	A	*farA*	100.00	100.00
Z1035	CARD	A	*farB*	97.77	100.00
Z1035	CARD	A	*macA*	96.10	100.00
Z1035	CARD	A	*macB*	97.36	100.00
Z1035	CARD	A	*mtrC*	97.34	100.00
Z1035	CARD	A	*mtrD*	99.19	100.00
Z1035	CARD	A	*mtrE*	93.67	99.93
Z1035	CARD	A	*mtrR*	97.79	100.00
Z1035	CARD	B	*farA*	100.00	100.00
Z1035	CARD	B	*farB*	97.77	100.00
Z1035	CARD	B	*macA*	96.10	100.00
Z1035	CARD	B	*macB*	97.36	100.00
Z1035	CARD	B	*mtrC*	97.34	100.00
Z1035	CARD	B	*mtrD*	99.19	100.00
Z1035	CARD	B	*mtrE*	93.67	99.93
Z1035	CARD	B	*mtrR*	97.79	100.00
Z1035	CARD	C	*farA*	100.00	100.00
Z1035	CARD	C	*farB*	97.77	100.00
Z1035	CARD	C	*macA*	96.10	100.00
Z1035	CARD	C	*macB*	97.36	100.00

Z1035	CARD	C	*mtrC*	97.34	100.00
Z1035	CARD	C	*mtrD*	99.19	100.00
Z1035	CARD	C	*mtrE*	93.67	99.93
Z1035	CARD	C	*mtrR*	97.79	100.00
Z1054	CARD	A	*farA*	99.91	100.00
Z1054	CARD	A	*farB*	97.77	100.00
Z1054	CARD	A	*macA*	95.93	100.00
Z1054	CARD	A	*macB*	97.05	100.00
Z1054	CARD	A	*mtrC*	97.02	100.00
Z1054	CARD	A	*mtrD*	99.19	100.00
Z1054	CARD	A	*mtrE*	93.74	99.93
Z1054	CARD	A	*mtrR*	97.48	100.00
Z1054	CARD	B	*farA*	99.91	100.00
Z1054	CARD	B	*farB*	97.77	100.00
Z1054	CARD	B	*macA*	95.93	100.00
Z1054	CARD	B	*macB*	97.05	100.00
Z1054	CARD	B	*mtrC*	97.02	100.00
Z1054	CARD	B	*mtrD*	99.19	100.00
Z1054	CARD	B	*mtrE*	93.74	99.93
Z1054	CARD	B	*mtrR*	97.48	100.00
Z1054	CARD	C	*farA*	99.91	100.00
Z1054	CARD	C	*farB*	97.77	100.00
Z1054	CARD	C	*macA*	95.93	100.00
Z1054	CARD	C	*macB*	97.05	100.00
Z1054	CARD	C	*mtrC*	97.02	100.00
Z1054	CARD	C	*mtrD*	99.19	100.00
Z1054	CARD	C	*mtrE*	93.74	99.93
Z1054	CARD	C	*mtrR*	97.48	100.00
Z1073	CARD	A	*farA*	100.00	100.00
Z1073	CARD	A	*farB*	97.77	100.00
Z1073	CARD	A	*macA*	96.10	100.00
Z1073	CARD	A	*macB*	97.36	100.00
Z1073	CARD	A	*mtrC*	97.58	100.00
Z1073	CARD	A	*mtrD*	99.03	100.00
Z1073	CARD	A	*mtrE*	94.31	100.00
Z1073	CARD	A	*mtrR*	97.79	100.00
Z1073	CARD	B	*ermF*	99.65	100.00
Z1073	ResFinder	B	*ermF*	99.83	100.00
Z1073	ARG-ANNOT	B	*ermF*	99.65	100.00
Z1073	NDARO	B	*ermF*	99.65	63.82
Z1073	CARD	B	*farA*	100.00	100.00
Z1073	CARD	B	*farB*	97.77	100.00
Z1073	CARD	B	*macA*	96.10	100.00
Z1073	CARD	B	*macB*	97.36	100.00

Z1073	CARD	B	*mtrC*	97.58	100.00
Z1073	CARD	B	*mtrD*	99.03	100.00
Z1073	CARD	B	*mtrE*	94.31	100.00
Z1073	CARD	B	*mtrR*	97.79	100.00
Z1073	CARD	C	*farA*	100.00	100.00
Z1073	CARD	C	*farB*	97.77	100.00
Z1073	CARD	C	*macA*	96.10	100.00
Z1073	CARD	C	*macB*	97.36	100.00
Z1073	CARD	C	*mtrC*	97.58	100.00
Z1073	CARD	C	*mtrD*	99.03	100.00
Z1073	CARD	C	*mtrE*	94.31	100.00
Z1073	CARD	C	*mtrR*	97.79	100.00
Z1092	CARD	A	*farA*	100.00	100.00
Z1092	CARD	A	*farB*	97.77	100.00
Z1092	CARD	A	*macA*	96.01	100.00
Z1092	CARD	A	*macB*	97.36	100.00
Z1092	CARD	A	*mtrC*	97.34	100.00
Z1092	CARD	A	*mtrD*	99.19	100.00
Z1092	CARD	A	*mtrE*	93.67	99.93
Z1092	CARD	A	*mtrR*	97.79	100.00
Z1092	CARD	B	*farA*	100.00	100.00
Z1092	CARD	B	*farB*	97.77	100.00
Z1092	CARD	B	*macA*	96.01	100.00
Z1092	CARD	B	*macB*	97.36	100.00
Z1092	CARD	B	*mtrC*	97.34	100.00
Z1092	CARD	B	*mtrD*	99.19	100.00
Z1092	CARD	B	*mtrE*	93.67	99.93
Z1092	CARD	B	*mtrR*	97.79	100.00
Z1092	CARD	C	*farA*	100.00	100.00
Z1092	CARD	C	*farB*	97.77	100.00
Z1092	CARD	C	*macA*	96.01	100.00
Z1092	CARD	C	*macB*	97.36	100.00
Z1092	CARD	C	*mtrC*	97.34	100.00
Z1092	CARD	C	*mtrD*	99.19	100.00
Z1092	CARD	C	*mtrE*	93.67	99.93
Z1092	CARD	C	*mtrR*	97.79	100.00
Z1099	CARD	A	*farA*	100.00	100.00
Z1099	CARD	A	*farB*	97.77	100.00
Z1099	CARD	A	*macA*	96.10	100.00
Z1099	CARD	A	*macB*	97.36	100.00
Z1099	CARD	A	*mtrC*	97.34	100.00
Z1099	CARD	A	*mtrD*	99.19	100.00
Z1099	CARD	A	*mtrE*	93.67	99.93
Z1099	CARD	A	*mtrR*	97.79	100.00

Z1099	CARD	B	*farA*	100.00	100.00
Z1099	CARD	B	*farB*	97.77	100.00
Z1099	CARD	B	*macA*	96.10	100.00
Z1099	CARD	B	*macB*	97.36	100.00
Z1099	CARD	B	*mtrC*	97.34	100.00
Z1099	CARD	B	*mtrD*	99.19	100.00
Z1099	CARD	B	*mtrE*	93.67	99.93
Z1099	CARD	B	*mtrR*	97.79	100.00
Z1099	CARD	C	*farA*	100.00	100.00
Z1099	CARD	C	*farB*	97.77	100.00
Z1099	CARD	C	*macA*	96.10	100.00
Z1099	CARD	C	*macB*	97.36	100.00
Z1099	CARD	C	*mtrC*	97.34	100.00
Z1099	CARD	C	*mtrD*	99.19	100.00
Z1099	CARD	C	*mtrE*	93.67	99.93
Z1099	CARD	C	*mtrR*	97.79	100.00
Z1269	CARD	A	*farA*	100.00	100.00
Z1269	CARD	A	*farB*	97.77	100.00
Z1269	CARD	A	*macA*	96.61	100.00
Z1269	CARD	A	*macB*	97.16	100.00
Z1269	CARD	A	*mtrC*	97.50	100.00
Z1269	CARD	A	*mtrD*	99.22	100.00
Z1269	CARD	A	*mtrE*	93.74	99.93
Z1269	CARD	A	*mtrR*	97.32	100.00
Z1269	CARD	B	*farA*	100.00	100.00
Z1269	CARD	B	*farB*	97.77	100.00
Z1269	CARD	B	*macA*	96.61	100.00
Z1269	CARD	B	*macB*	97.16	100.00
Z1269	CARD	B	*mtrC*	97.50	100.00
Z1269	CARD	B	*mtrD*	99.22	100.00
Z1269	CARD	B	*mtrE*	93.74	99.93
Z1269	CARD	B	*mtrR*	97.32	100.00
Z1269	CARD	C	*farA*	100.00	100.00
Z1269	CARD	C	*farB*	97.77	100.00
Z1269	CARD	C	*macA*	96.61	100.00
Z1269	CARD	C	*macB*	97.16	100.00
Z1269	CARD	C	*mtrC*	97.50	100.00
Z1269	CARD	C	*mtrD*	99.22	100.00
Z1269	CARD	C	*mtrE*	93.74	99.93
Z1269	CARD	C	*mtrR*	97.32	100.00
Z1275	CARD	A	*farA*	96.80	100.00
Z1275	CARD	A	*farB*	97.77	100.00
Z1275	CARD	A	*macA*	96.10	100.00
Z1275	CARD	A	*macB*	96.95	100.00

Z1275	CARD	A	*mtrC*	97.34	100.00
Z1275	CARD	A	*mtrD*	99.19	100.00
Z1275	CARD	A	*mtrE*	93.67	99.93
Z1275	CARD	A	*mtrR*	97.79	100.00
Z1275	CARD	B	*farA*	96.80	100.00
Z1275	CARD	B	*farB*	97.77	100.00
Z1275	CARD	B	*macA*	96.10	100.00
Z1275	CARD	B	*macB*	96.95	100.00
Z1275	CARD	B	*mtrC*	97.34	100.00
Z1275	CARD	B	*mtrD*	99.19	100.00
Z1275	CARD	B	*mtrE*	93.67	99.93
Z1275	CARD	B	*mtrR*	97.79	100.00
Z1275	CARD	C	*farA*	96.80	100.00
Z1275	CARD	C	*farB*	97.77	100.00
Z1275	CARD	C	*macA*	96.10	100.00
Z1275	CARD	C	*macB*	96.95	100.00
Z1275	CARD	C	*mtrC*	97.34	100.00
Z1275	CARD	C	*mtrD*	99.19	100.00
Z1275	CARD	C	*mtrE*	93.67	99.93
Z1275	CARD	C	*mtrR*	97.79	100.00
Z1392	CARD	A	*farA*	100.00	100.00
Z1392	CARD	A	*farB*	97.77	100.00
Z1392	CARD	A	*macA*	96.10	100.00
Z1392	CARD	A	*macB*	97.36	100.00
Z1392	CARD	A	*mtrC*	97.34	100.00
Z1392	CARD	A	*mtrD*	99.19	100.00
Z1392	CARD	A	*mtrE*	93.67	99.93
Z1392	CARD	A	*mtrR*	97.79	100.00
Z1392	CARD	B	*farA*	100.00	100.00
Z1392	CARD	B	*farB*	97.77	100.00
Z1392	CARD	B	*macA*	96.10	100.00
Z1392	CARD	B	*macB*	97.36	100.00
Z1392	CARD	B	*mtrC*	97.34	100.00
Z1392	CARD	B	*mtrD*	99.19	100.00
Z1392	CARD	B	*mtrE*	93.67	99.93
Z1392	CARD	B	*mtrR*	97.79	100.00
Z1392	CARD	C	*farA*	100.00	100.00
Z1392	CARD	C	*farB*	97.77	100.00
Z1392	CARD	C	*macA*	96.10	100.00
Z1392	CARD	C	*macB*	97.36	100.00
Z1392	CARD	C	*mtrC*	97.34	100.00
Z1392	CARD	C	*mtrD*	99.19	100.00
Z1392	CARD	C	*mtrE*	93.67	99.93
Z1392	CARD	C	*mtrR*	97.79	100.00

Z1439	CARD	A	*farA*	100.00	100.00
Z1439	CARD	A	*farB*	97.77	100.00
Z1439	CARD	A	*macA*	95.93	100.00
Z1439	CARD	A	*macB*	97.36	100.00
Z1439	CARD	A	*mtrC*	97.34	100.00
Z1439	CARD	A	*mtrD*	99.19	100.00
Z1439	CARD	A	*mtrE*	93.67	99.93
Z1439	CARD	A	*mtrR*	97.79	100.00
Z1439	CARD	B	*farA*	100.00	100.00
Z1439	CARD	B	*farB*	97.77	100.00
Z1439	CARD	B	*macA*	95.93	100.00
Z1439	CARD	B	*macB*	97.36	100.00
Z1439	CARD	B	*mtrC*	97.34	100.00
Z1439	CARD	B	*mtrD*	99.19	100.00
Z1439	CARD	B	*mtrE*	93.67	99.93
Z1439	CARD	B	*mtrR*	97.79	100.00
Z1439	CARD	C	*farA*	100.00	100.00
Z1439	CARD	C	*farB*	97.77	100.00
Z1439	CARD	C	*macA*	95.93	100.00
Z1439	CARD	C	*macB*	97.36	100.00
Z1439	CARD	C	*mtrC*	97.34	100.00
Z1439	CARD	C	*mtrD*	99.19	100.00
Z1439	CARD	C	*mtrE*	93.67	99.93
Z1439	CARD	C	*mtrR*	97.79	100.00
Z1466	CARD	A	*farA*	100.00	100.00
Z1466	CARD	A	*farB*	97.77	100.00
Z1466	CARD	A	*macA*	96.10	100.00
Z1466	CARD	A	*macB*	97.36	100.00
Z1466	CARD	A	*mtrC*	97.34	100.00
Z1466	CARD	A	*mtrD*	99.13	100.00
Z1466	CARD	A	*mtrE*	93.67	99.93
Z1466	CARD	A	*mtrR*	96.69	100.00
Z1466	CARD	B	*farA*	100.00	100.00
Z1466	CARD	B	*farB*	97.77	100.00
Z1466	CARD	B	*macA*	96.10	100.00
Z1466	CARD	B	*macB*	97.36	100.00
Z1466	CARD	B	*mtrC*	97.34	100.00
Z1466	CARD	B	*mtrD*	99.13	100.00
Z1466	CARD	B	*mtrE*	93.67	99.93
Z1466	CARD	B	*mtrR*	96.69	100.00
Z1466	CARD	C	*farA*	100.00	100.00
Z1466	CARD	C	*farB*	97.77	100.00
Z1466	CARD	C	*macA*	96.10	100.00
Z1466	CARD	C	*macB*	97.36	100.00

Z1466	CARD	C	*mtrC*	97.34	100.00
Z1466	CARD	C	*mtrD*	99.16	100.00
Z1466	CARD	C	*mtrE*	93.67	99.93
Z1466	CARD	C	*mtrR*	96.69	100.00
Z1534	CARD	A	*farA*	100.00	100.00
Z1534	CARD	A	*farB*	97.77	100.00
Z1534	CARD	A	*macA*	96.78	100.00
Z1534	CARD	A	*macB*	96.74	100.00
Z1534	CARD	A	*mtrC*	97.10	100.00
Z1534	CARD	A	*mtrD*	99.38	100.00
Z1534	CARD	A	*mtrE*	94.45	99.93
Z1534	CARD	A	*mtrR*	97.79	100.00
Z1534	CARD	B	*farA*	100.00	100.00
Z1534	CARD	B	*farB*	97.77	100.00
Z1534	CARD	B	*macA*	96.78	100.00
Z1534	CARD	B	*macB*	96.74	100.00
Z1534	CARD	B	*mtrC*	97.10	100.00
Z1534	CARD	B	*mtrD*	99.38	100.00
Z1534	CARD	B	*mtrE*	94.45	99.93
Z1534	CARD	B	*mtrR*	97.79	100.00
Z1534	CARD	C	*farA*	100.00	100.00
Z1534	CARD	C	*farB*	97.77	100.00
Z1534	CARD	C	*macA*	96.78	100.00
Z1534	CARD	C	*macB*	96.74	100.00
Z1534	CARD	C	*mtrC*	97.10	100.00
Z1534	CARD	C	*mtrD*	99.38	100.00
Z1534	CARD	C	*mtrE*	94.45	99.93
Z1534	CARD	C	*mtrR*	97.79	100.00
Z3842	CARD	A	*farA*	100.00	100.00
Z3842	CARD	A	*farB*	100.00	100.00
Z3842	CARD	A	*macA*	96.10	100.00
Z3842	CARD	A	*macB*	96.74	100.00
Z3842	CARD	A	*mtrC*	100.00	100.00
Z3842	CARD	A	*mtrD*	100.00	100.00
Z3842	CARD	A	*mtrE*	94.52	99.93
Z3842	CARD	A	*mtrR*	97.48	100.00
Z3842	CARD	B	*farA*	100.00	100.00
Z3842	CARD	B	*farB*	100.00	100.00
Z3842	CARD	B	*macA*	96.10	100.00
Z3842	CARD	B	*macB*	96.74	100.00
Z3842	CARD	B	*mtrC*	100.00	100.00
Z3842	CARD	B	*mtrD*	100.00	100.00
Z3842	CARD	B	*mtrE*	94.52	99.93
Z3842	CARD	B	*mtrR*	97.48	100.00

Z3842	CARD	C	*farA*	100.00	100.00
Z3842	CARD	C	*farB*	100.00	100.00
Z3842	CARD	C	*macA*	96.10	100.00
Z3842	CARD	C	*macB*	96.74	100.00
Z3842	CARD	C	*mtrC*	100.00	100.00
Z3842	CARD	C	*mtrD*	100.00	100.00
Z3842	CARD	C	*mtrE*	94.52	99.93
Z3842	CARD	C	*mtrR*	97.48	100.00
Z3906	CARD	A	*farA*	99.91	100.00
Z3906	CARD	A	*farB*	97.77	100.00
Z3906	CARD	A	*macA*	96.10	100.00
Z3906	CARD	A	*macB*	96.59	100.00
Z3906	CARD	A	*mtrC*	97.02	100.00
Z3906	CARD	A	*mtrD*	99.22	100.00
Z3906	CARD	A	*mtrE*	93.74	99.93
Z3906	CARD	A	*mtrR*	97.48	100.00
Z3906	CARD	B	*farA*	99.91	100.00
Z3906	CARD	B	*farB*	97.77	100.00
Z3906	CARD	B	*macA*	96.10	100.00
Z3906	CARD	B	*macB*	96.59	100.00
Z3906	CARD	B	*mtrC*	97.02	100.00
Z3906	CARD	B	*mtrD*	99.22	100.00
Z3906	CARD	B	*mtrE*	93.74	99.93
Z3906	CARD	B	*mtrR*	97.48	100.00
Z3906	CARD	C	*farA*	99.91	100.00
Z3906	CARD	C	*farB*	97.77	100.00
Z3906	CARD	C	*macA*	96.10	100.00
Z3906	CARD	C	*macB*	96.59	100.00
Z3906	CARD	C	*mtrC*	97.02	100.00
Z3906	CARD	C	*mtrD*	99.22	100.00
Z3906	CARD	C	*mtrE*	93.74	99.93
Z3906	CARD	C	*mtrR*	97.48	100.00
Z4242	CARD	A	*farA*	99.19	100.00
Z4242	CARD	A	*farB*	97.84	100.00
Z4242	CARD	A	*macA*	95.59	100.00
Z4242	CARD	A	*macB*	96.95	100.00
Z4242	CARD	A	*mtrC*	97.18	100.00
Z4242	CARD	A	*mtrD*	99.13	100.00
Z4242	CARD	A	*mtrE*	94.31	99.93
Z4242	CARD	B	*farA*	99.19	100.00
Z4242	CARD	B	*farB*	97.84	100.00
Z4242	CARD	B	*macA*	95.59	100.00
Z4242	CARD	B	*macB*	96.95	100.00
Z4242	CARD	B	*mtrC*	97.18	100.00

Z4242	CARD	B	*mtrD*	99.13	100.00
Z4242	CARD	B	*mtrE*	94.31	99.93
Z4242	CARD	B	*mtrR*	97.32	100.00
Z4242	CARD	C	*farA*	99.19	100.00
Z4242	CARD	C	*farB*	97.84	100.00
Z4242	CARD	C	*macA*	95.59	100.00
Z4242	CARD	C	*macB*	96.95	100.00
Z4242	CARD	C	*mtrC*	97.18	100.00
Z4242	CARD	C	*mtrD*	99.13	100.00
Z4242	CARD	C	*mtrE*	94.31	99.93
Z4242	CARD	C	*mtrR*	97.32	100.00
Z4662	CARD	A	*farA*	99.64	100.00
Z4662	CARD	A	*farB*	95.02	100.00
Z4662	CARD	A	*macA*	96.27	100.00
Z4662	CARD	A	*macB*	97.42	100.00
Z4662	CARD	A	*mtrC*	97.18	100.00
Z4662	CARD	A	*mtrD*	99.03	100.00
Z4662	CARD	A	*mtrE*	94.31	99.93
Z4662	CARD	A	*mtrR*	97.48	100.00
Z4662	CARD	B	*farA*	99.64	100.00
Z4662	CARD	B	*farB*	95.02	100.00
Z4662	CARD	B	*macA*	96.27	100.00
Z4662	CARD	B	*macB*	97.42	100.00
Z4662	CARD	B	*mtrC*	97.18	100.00
Z4662	CARD	B	*mtrD*	99.03	100.00
Z4662	CARD	B	*mtrE*	94.31	99.93
Z4662	CARD	B	*mtrR*	97.48	100.00
Z4662	CARD	C	*farA*	99.64	100.00
Z4662	CARD	C	*farB*	95.02	100.00
Z4662	CARD	C	*macA*	96.27	100.00
Z4662	CARD	C	*macB*	97.42	100.00
Z4662	CARD	C	*mtrC*	97.18	100.00
Z4662	CARD	C	*mtrD*	99.03	100.00
Z4662	CARD	C	*mtrE*	94.31	99.93
Z4662	CARD	C	*mtrR*	97.48	100.00
Z4664	CARD	A	*farA*	100.00	100.00
Z4664	CARD	A	*farB*	100.00	100.00
Z4664	CARD	A	*macA*	96.52	100.00
Z4664	CARD	A	*macB*	97.21	100.00
Z4664	CARD	A	*mtrC*	100.00	100.00
Z4664	CARD	A	*mtrD*	100.00	100.00
Z4664	CARD	A	*mtrE*	94.52	99.93
Z4664	CARD	A	*mtrR*	97.48	100.00
Z4664	CARD	B	*farA*	100.00	100.00

Z4664	CARD	B	*farB*	100.00	100.00
Z4664	CARD	B	*macA*	96.52	100.00
Z4664	CARD	B	*macB*	97.21	100.00
Z4664	CARD	B	*mtrC*	100.00	100.00
Z4664	CARD	B	*mtrD*	100.00	100.00
Z4664	CARD	B	*mtrE*	94.52	99.93
Z4664	CARD	B	*mtrR*	97.48	100.00
Z4664	CARD	C	*farA*	100.00	100.00
Z4664	CARD	C	*farB*	100.00	100.00
Z4664	CARD	C	*macA*	96.52	100.00
Z4664	CARD	C	*macB*	97.21	100.00
Z4664	CARD	C	*mtrC*	100.00	100.00
Z4664	CARD	C	*mtrD*	100.00	100.00
Z4664	CARD	C	*mtrE*	94.52	99.93
Z4664	CARD	C	*mtrR*	97.48	100.00
Z4665	CARD	A	*farA*	100.00	100.00
Z4665	CARD	A	*farB*	97.77	100.00
Z4665	CARD	A	*macA*	96.10	100.00
Z4665	CARD	A	*macB*	97.36	100.00
Z4665	CARD	A	*mtrC*	97.58	100.00
Z4665	CARD	A	*mtrD*	99.03	100.00
Z4665	CARD	A	*mtrE*	94.31	100.00
Z4665	CARD	A	*mtrR*	97.79	100.00
Z4665	CARD	B	*farA*	100.00	100.00
Z4665	CARD	B	*farB*	97.77	100.00
Z4665	CARD	B	*macA*	96.10	100.00
Z4665	CARD	B	*macB*	97.36	100.00
Z4665	CARD	B	*mtrC*	97.58	100.00
Z4665	CARD	B	*mtrD*	99.03	100.00
Z4665	CARD	B	*mtrE*	94.31	100.00
Z4665	CARD	B	*mtrR*	97.79	100.00
Z4665	CARD	C	*farA*	100.00	100.00
Z4665	CARD	C	*farB*	97.77	100.00
Z4665	CARD	C	*macA*	96.10	100.00
Z4665	CARD	C	*macB*	97.36	100.00
Z4665	CARD	C	*mtrC*	97.58	100.00
Z4665	CARD	C	*mtrD*	99.03	100.00
Z4665	CARD	C	*mtrE*	94.31	100.00
Z4665	CARD	C	*mtrR*	97.79	100.00
Z4667	CARD	A	*farA*	99.48	100.00
Z4667	CARD	A	*farB*	97.18	100.00
Z4667	CARD	A	*macA*	96.01	100.00
Z4667	CARD	A	*macB*	96.85	100.00
Z4667	CARD	A	*mtrC*	96.61	100.00

Z4667	CARD	A	*mtrD*	99.25	100.00
Z4667	CARD	A	*mtrE*	94.60	99.93
Z4667	CARD	A	*mtrR*	97.95	100.00
Z4667	CARD	B	*farA*	99.48	100.00
Z4667	CARD	B	*farB*	97.18	100.00
Z4667	CARD	B	*macA*	96.01	100.00
Z4667	CARD	B	*macB*	96.85	100.00
Z4667	CARD	B	*mtrC*	96.61	100.00
Z4667	CARD	B	*mtrD*	99.25	100.00
Z4667	CARD	B	*mtrE*	94.60	99.93
Z4667	CARD	B	*mtrR*	97.95	100.00
Z4667	CARD	C	*farA*	99.48	100.00
Z4667	CARD	C	*farB*	97.18	100.00
Z4667	CARD	C	*macA*	96.01	100.00
Z4667	CARD	C	*macB*	96.85	100.00
Z4667	CARD	C	*mtrC*	96.61	100.00
Z4667	CARD	C	*mtrD*	99.25	100.00
Z4667	CARD	C	*mtrE*	94.60	99.93
Z4667	CARD	C	*mtrR*	97.95	100.00
Z4671	CARD	A	*farA*	99.64	100.00
Z4671	CARD	A	*farB*	95.02	100.00
Z4671	CARD	A	*macA*	96.27	100.00
Z4671	CARD	A	*macB*	97.42	100.00
Z4671	CARD	A	*mtrC*	97.34	100.00
Z4671	CARD	A	*mtrD*	99.25	100.00
Z4671	CARD	A	*mtrE*	94.31	99.93
Z4671	CARD	A	*mtrR*	97.16	100.00
Z4671	CARD	B	*farA*	99.64	100.00
Z4671	CARD	B	*farB*	95.02	100.00
Z4671	CARD	B	*macA*	96.27	100.00
Z4671	CARD	B	*macB*	97.42	100.00
Z4671	CARD	B	*mtrC*	97.34	100.00
Z4671	CARD	B	*mtrD*	99.25	100.00
Z4671	CARD	B	*mtrE*	94.31	99.93
Z4671	CARD	B	*mtrR*	97.16	100.00
Z4671	CARD	C	*farA*	99.64	100.00
Z4671	CARD	C	*farB*	95.02	100.00
Z4671	CARD	C	*macA*	96.27	100.00
Z4671	CARD	C	*macB*	97.42	100.00
Z4671	CARD	C	*mtrC*	97.34	100.00
Z4671	CARD	C	*mtrD*	99.25	100.00
Z4671	CARD	C	*mtrE*	94.31	99.93
Z4671	CARD	C	*mtrR*	97.16	100.00
Z4672	CARD	A	*farA*	100.00	100.00

Z4672	CARD	A	*farB*	100.00	100.00
Z4672	CARD	A	*macA*	96.10	100.00
Z4672	CARD	A	*macB*	97.42	100.00
Z4672	CARD	A	*mtrC*	100.00	100.00
Z4672	CARD	A	*mtrD*	100.00	100.00
Z4672	CARD	A	*mtrE*	94.52	99.93
Z4672	CARD	A	*mtrR*	97.48	100.00
Z4672	CARD	B	*farA*	100.00	100.00
Z4672	CARD	B	*farB*	100.00	100.00
Z4672	CARD	B	*macA*	96.10	100.00
Z4672	CARD	B	*macB*	97.42	100.00
Z4672	CARD	B	*mtrC*	100.00	100.00
Z4672	CARD	B	*mtrD*	100.00	100.00
Z4672	CARD	B	*mtrE*	94.52	99.93
Z4672	CARD	B	*mtrR*	97.48	100.00
Z4672	CARD	C	*farA*	100.00	100.00
Z4672	CARD	C	*farB*	100.00	100.00
Z4672	CARD	C	*macA*	96.10	100.00
Z4672	CARD	C	*macB*	97.42	100.00
Z4672	CARD	C	*mtrC*	100.00	100.00
Z4672	CARD	C	*mtrD*	100.00	100.00
Z4672	CARD	C	*mtrE*	94.52	99.93
Z4672	CARD	C	*mtrR*	97.48	100.00
Z4673	CARD	A	*farA*	98.87	100.00
Z4673	CARD	A	*farB*	96.79	100.00
Z4673	CARD	A	*macA*	95.67	100.00
Z4673	CARD	A	*macB*	96.95	100.00
Z4673	CARD	A	*mtrC*	97.10	100.00
Z4673	CARD	A	*mtrD*	98.81	100.00
Z4673	CARD	A	*mtrE*	94.52	99.93
Z4673	CARD	A	*mtrR*	97.95	100.00
Z4673	CARD	B	*farA*	98.87	100.00
Z4673	CARD	B	*farB*	96.79	100.00
Z4673	CARD	B	*macA*	95.67	100.00
Z4673	CARD	B	*macB*	96.95	100.00
Z4673	CARD	B	*mtrC*	97.10	100.00
Z4673	CARD	B	*mtrD*	98.81	100.00
Z4673	CARD	B	*mtrE*	94.52	99.93
Z4673	CARD	B	*mtrR*	97.95	100.00
Z4673	CARD	C	*farA*	98.87	100.00
Z4673	CARD	C	*farB*	96.79	100.00
Z4673	CARD	C	*macA*	95.67	100.00
Z4673	CARD	C	*macB*	96.95	100.00
Z4673	CARD	C	*mtrC*	97.10	100.00

Z4673	CARD	C	*mtrD*	98.81	100.00
Z4673	CARD	C	*mtrE*	94.52	99.93
Z4673	CARD	C	*mtrR*	97.95	100.00
Z4674	CARD	A	*farA*	98.30	100.00
Z4674	CARD	A	*farB*	96.27	100.00
Z4674	CARD	A	*macA*	96.10	100.00
Z4674	CARD	A	*macB*	97.36	100.00
Z4674	CARD	A	*mtrC*	98.15	100.00
Z4674	CARD	A	*mtrD*	99.06	100.00
Z4674	CARD	A	*mtrE*	94.31	99.93
Z4674	CARD	A	*mtrR*	97.63	100.00
Z4674	CARD	B	*farA*	98.30	100.00
Z4674	CARD	B	*farB*	96.27	100.00
Z4674	CARD	B	*macA*	96.10	100.00
Z4674	CARD	B	*macB*	97.36	100.00
Z4674	CARD	B	*mtrC*	98.15	100.00
Z4674	CARD	B	*mtrD*	99.06	100.00
Z4674	CARD	B	*mtrE*	94.31	99.93
Z4674	CARD	B	*mtrR*	97.63	100.00
Z4674	CARD	C	*farA*	98.30	100.00
Z4674	CARD	C	*farB*	96.27	100.00
Z4674	CARD	C	*macA*	96.10	100.00
Z4674	CARD	C	*macB*	97.36	100.00
Z4674	CARD	C	*mtrC*	98.15	100.00
Z4674	CARD	C	*mtrD*	99.06	100.00
Z4674	CARD	C	*mtrE*	94.31	99.93
Z4674	CARD	C	*mtrR*	97.63	100.00
Z4675	CARD	A	*farA*	98.53	100.00
Z4675	CARD	A	*farB*	97.71	100.00
Z4675	CARD	A	*macA*	96.10	100.00
Z4675	CARD	A	*macB*	97.16	100.00
Z4675	CARD	A	*mtrC*	98.31	100.00
Z4675	CARD	A	*mtrD*	98.50	100.00
Z4675	CARD	A	*mtrE*	94.52	99.93
Z4675	CARD	A	*mtrR*	97.79	100.00
Z4675	CARD	B	*farA*	98.53	100.00
Z4675	CARD	B	*farB*	97.71	100.00
Z4675	CARD	B	*macA*	96.10	100.00
Z4675	CARD	B	*macB*	97.16	100.00
Z4675	CARD	B	*mtrC*	98.31	100.00
Z4675	CARD	B	*mtrD*	98.50	100.00
Z4675	CARD	B	*mtrE*	94.52	99.93
Z4675	CARD	B	*mtrR*	97.79	100.00
Z4675	CARD	C	*farA*	98.53	100.00

Z4675	CARD	C	*farB*	97.71	100.00
Z4675	CARD	C	*macA*	96.10	100.00
Z4675	CARD	C	*macB*	97.16	100.00
Z4675	CARD	C	*mtrC*	98.31	100.00
Z4675	CARD	C	*mtrD*	98.50	100.00
Z4675	CARD	C	*mtrE*	94.52	99.93
Z4675	CARD	C	*mtrR*	97.79	100.00
Z4676	CARD	A	*farA*	98.30	100.00
Z4676	CARD	A	*farB*	96.27	100.00
Z4676	CARD	A	*macA*	96.10	100.00
Z4676	CARD	A	*macB*	97.42	100.00
Z4676	CARD	A	*mtrC*	98.15	100.00
Z4676	CARD	A	*mtrD*	99.06	100.00
Z4676	CARD	A	*mtrE*	94.31	99.93
Z4676	CARD	A	*mtrR*	97.63	100.00
Z4676	CARD	B	*farA*	98.30	100.00
Z4676	CARD	B	*farB*	96.27	100.00
Z4676	CARD	B	*macA*	96.10	100.00
Z4676	CARD	B	*macB*	97.42	100.00
Z4676	CARD	B	*mtrC*	98.15	100.00
Z4676	CARD	B	*mtrD*	99.06	100.00
Z4676	CARD	B	*mtrE*	94.31	99.93
Z4676	CARD	B	*mtrR*	97.63	100.00
Z4676	CARD	C	*farA*	98.30	100.00
Z4676	CARD	C	*farB*	96.27	100.00
Z4676	CARD	C	*macA*	96.10	100.00
Z4676	CARD	C	*macB*	97.42	100.00
Z4676	CARD	C	*mtrC*	98.15	100.00
Z4676	CARD	C	*mtrD*	99.06	100.00
Z4676	CARD	C	*mtrE*	94.31	99.93
Z4676	CARD	C	*mtrR*	97.63	100.00
Z4678	CARD	A	*farA*	98.87	100.00
Z4678	CARD	A	*farB*	96.92	100.00
Z4678	CARD	A	*macA*	95.84	100.00
Z4678	CARD	A	*macB*	97.36	100.00
Z4678	CARD	A	*mtrC*	98.15	100.00
Z4678	CARD	A	*mtrD*	98.78	100.00
Z4678	CARD	A	*mtrE*	94.38	99.93
Z4678	CARD	A	*mtrR*	97.63	100.00
Z4678	CARD	B	*farA*	98.87	100.00
Z4678	CARD	B	*farB*	96.92	100.00
Z4678	CARD	B	*macA*	95.84	100.00
Z4678	CARD	B	*macB*	97.36	100.00
Z4678	CARD	B	*mtrC*	98.15	100.00

Z4678	CARD	B	*mtrD*	98.78	100.00
Z4678	CARD	B	*mtrE*	94.38	99.93
Z4678	CARD	B	*mtrR*	97.63	100.00
Z4678	CARD	C	*farA*	98.87	100.00
Z4678	CARD	C	*farB*	96.92	100.00
Z4678	CARD	C	*macA*	95.84	100.00
Z4678	CARD	C	*macB*	97.36	100.00
Z4678	CARD	C	*mtrC*	98.15	100.00
Z4678	CARD	C	*mtrD*	98.78	100.00
Z4678	CARD	C	*mtrE*	94.38	99.93
Z4678	CARD	C	*mtrR*	97.63	100.00
Z4681	CARD	A	*farA*	98.30	100.00
Z4681	CARD	A	*farB*	96.27	100.00
Z4681	CARD	A	*macA*	96.18	100.00
Z4681	CARD	A	*macB*	96.95	100.00
Z4681	CARD	A	*mtrC*	98.15	100.00
Z4681	CARD	A	*mtrD*	99.06	100.00
Z4681	CARD	A	*mtrE*	94.31	99.93
Z4681	CARD	A	*mtrR*	97.63	100.00
Z4681	CARD	B	*farA*	98.30	100.00
Z4681	CARD	B	*farB*	96.27	100.00
Z4681	CARD	B	*macA*	96.18	100.00
Z4681	CARD	B	*macB*	96.95	100.00
Z4681	CARD	B	*mtrC*	98.15	100.00
Z4681	CARD	B	*mtrD*	99.06	100.00
Z4681	CARD	B	*mtrE*	94.31	99.93
Z4681	CARD	B	*mtrR*	97.63	100.00
Z4681	CARD	C	*farA*	98.30	100.00
Z4681	CARD	C	*farB*	96.27	100.00
Z4681	CARD	C	*macA*	96.18	100.00
Z4681	CARD	C	*macB*	96.95	100.00
Z4681	CARD	C	*mtrC*	98.15	100.00
Z4681	CARD	C	*mtrD*	99.06	100.00
Z4681	CARD	C	*mtrE*	94.31	99.93
Z4681	CARD	C	*mtrR*	97.63	100.00
Z4682	CARD	A	*farA*	99.39	100.00
Z4682	CARD	A	*farB*	96.27	100.00
Z4682	CARD	A	*macA*	96.61	100.00
Z4682	CARD	A	*macB*	97.16	100.00
Z4682	CARD	A	*mtrC*	97.66	100.00
Z4682	CARD	A	*mtrD*	98.97	100.00
Z4682	CARD	A	*mtrE*	94.38	99.93
Z4682	CARD	A	*mtrR*	97.48	100.00
Z4682	CARD	B	*farA*	99.39	100.00

Z4682	CARD	B	*farB*	96.27	100.00
Z4682	CARD	B	*macA*	96.61	100.00
Z4682	CARD	B	*macB*	97.16	100.00
Z4682	CARD	B	*mtrC*	97.66	100.00
Z4682	CARD	B	*mtrD*	98.97	100.00
Z4682	CARD	B	*mtrE*	94.38	99.93
Z4682	CARD	B	*mtrR*	97.48	100.00
Z4682	CARD	C	*farA*	99.39	100.00
Z4682	CARD	C	*farB*	96.27	100.00
Z4682	CARD	C	*macA*	96.61	100.00
Z4682	CARD	C	*macB*	97.16	100.00
Z4682	CARD	C	*mtrC*	97.66	100.00
Z4682	CARD	C	*mtrD*	98.97	100.00
Z4682	CARD	C	*mtrE*	94.38	99.93
Z4682	CARD	C	*mtrR*	97.48	100.00
Z4683	CARD	A	*farA*	100.00	100.00
Z4683	CARD	A	*farB*	97.77	100.00
Z4683	CARD	A	*macA*	96.52	100.00
Z4683	CARD	A	*macB*	96.95	100.00
Z4683	CARD	A	*mtrC*	96.61	100.00
Z4683	CARD	A	*mtrD*	98.57	100.00
Z4683	CARD	A	*mtrE*	94.52	99.93
Z4683	CARD	A	*mtrR*	97.95	100.00
Z4683	CARD	B	*farA*	100.00	100.00
Z4683	CARD	B	*farB*	97.77	100.00
Z4683	CARD	B	*macA*	96.52	100.00
Z4683	CARD	B	*macB*	96.95	100.00
Z4683	CARD	B	*mtrC*	96.61	100.00
Z4683	CARD	B	*mtrD*	98.57	100.00
Z4683	CARD	B	*mtrE*	94.52	99.93
Z4683	CARD	B	*mtrR*	97.95	100.00
Z4683	CARD	C	*farA*	100.00	100.00
Z4683	CARD	C	*farB*	97.77	100.00
Z4683	CARD	C	*macA*	96.52	100.00
Z4683	CARD	C	*macB*	96.95	100.00
Z4683	CARD	C	*mtrC*	96.61	100.00
Z4683	CARD	C	*mtrD*	98.57	100.00
Z4683	CARD	C	*mtrE*	94.52	99.93
Z4683	CARD	C	*mtrR*	97.95	100.00
Z4684	CARD	A	*farA*	99.39	100.00
Z4684	CARD	A	*farB*	96.27	100.00
Z4684	CARD	A	*macA*	95.84	100.00
Z4684	CARD	A	*macB*	97.16	100.00
Z4684	CARD	A	*mtrC*	97.58	100.00

Z4684	CARD	A	*mtrD*	99.16	100.00
Z4684	CARD	A	*mtrE*	94.38	99.93
Z4684	CARD	A	*mtrR*	97.32	100.00
Z4684	CARD	B	*farA*	99.39	100.00
Z4684	CARD	B	*farB*	96.27	100.00
Z4684	CARD	B	*macA*	95.84	100.00
Z4684	CARD	B	*macB*	97.16	100.00
Z4684	CARD	B	*mtrC*	97.58	100.00
Z4684	CARD	B	*mtrD*	99.16	100.00
Z4684	CARD	B	*mtrE*	94.38	99.93
Z4684	CARD	B	*mtrR*	97.32	100.00
Z4684	CARD	C	*farA*	99.39	100.00
Z4684	CARD	C	*farB*	96.27	100.00
Z4684	CARD	C	*macA*	95.84	100.00
Z4684	CARD	C	*macB*	97.16	100.00
Z4684	CARD	C	*mtrC*	97.58	100.00
Z4684	CARD	C	*mtrD*	99.16	100.00
Z4684	CARD	C	*mtrE*	94.38	99.93
Z4684	CARD	C	*mtrR*	97.32	100.00
Z4685	CARD	A	*farA*	99.39	100.00
Z4685	CARD	A	*farB*	96.27	100.00
Z4685	CARD	A	*macA*	95.84	100.00
Z4685	CARD	A	*macB*	97.16	100.00
Z4685	CARD	A	*mtrC*	97.58	100.00
Z4685	CARD	A	*mtrD*	99.16	100.00
Z4685	CARD	A	*mtrE*	94.38	99.93
Z4685	CARD	A	*mtrR*	97.32	100.00
Z4685	CARD	B	*farA*	99.39	100.00
Z4685	CARD	B	*farB*	96.27	100.00
Z4685	CARD	B	*macA*	95.84	100.00
Z4685	CARD	B	*macB*	97.16	100.00
Z4685	CARD	B	*mtrC*	97.58	100.00
Z4685	CARD	B	*mtrD*	99.16	100.00
Z4685	CARD	B	*mtrE*	94.38	99.93
Z4685	CARD	B	*mtrR*	97.32	100.00
Z4685	CARD	C	*farA*	99.39	100.00
Z4685	CARD	C	*farB*	96.27	100.00
Z4685	CARD	C	*macA*	95.84	100.00
Z4685	CARD	C	*macB*	97.16	100.00
Z4685	CARD	C	*mtrC*	97.58	100.00
Z4685	CARD	C	*mtrD*	99.16	100.00
Z4685	CARD	C	*mtrE*	94.38	99.93
Z4685	CARD	C	*mtrR*	97.32	100.00
Z4686	CARD	A	*farA*	99.05	100.00

Z4686	CARD	A	*farB*	97.90	100.00
Z4686	CARD	A	*macA*	95.93	100.00
Z4686	CARD	A	*macB*	97.36	100.00
Z4686	CARD	A	*mtrC*	97.26	100.00
Z4686	CARD	A	*mtrD*	98.78	100.00
Z4686	CARD	A	*mtrE*	94.52	99.93
Z4686	CARD	A	*mtrR*	97.95	100.00
Z4686	CARD	B	*farA*	99.05	100.00
Z4686	CARD	B	*farB*	97.90	100.00
Z4686	CARD	B	*macA*	95.93	100.00
Z4686	CARD	B	*macB*	97.36	100.00
Z4686	CARD	B	*mtrC*	97.26	100.00
Z4686	CARD	B	*mtrD*	98.78	100.00
Z4686	CARD	B	*mtrE*	94.52	99.93
Z4686	CARD	B	*mtrR*	97.95	100.00
Z4686	CARD	C	*farA*	99.05	100.00
Z4686	CARD	C	*farB*	97.90	100.00
Z4686	CARD	C	*macA*	95.93	100.00
Z4686	CARD	C	*macB*	97.36	100.00
Z4686	CARD	C	*mtrC*	97.26	100.00
Z4686	CARD	C	*mtrD*	98.78	100.00
Z4686	CARD	C	*mtrE*	94.52	99.93
Z4686	CARD	C	*mtrR*	97.95	100.00
Z4687	CARD	A	*farA*	99.10	100.00
Z4687	CARD	A	*farB*	97.18	100.00
Z4687	CARD	A	*macA*	95.93	100.00
Z4687	CARD	A	*macB*	97.31	100.00
Z4687	CARD	A	*mtrC*	97.50	100.00
Z4687	CARD	A	*mtrD*	98.47	100.00
Z4687	CARD	A	*mtrE*	94.38	99.93
Z4687	CARD	A	*mtrR*	97.63	100.00
Z4687	CARD	B	*farA*	99.10	100.00
Z4687	CARD	B	*farB*	97.18	100.00
Z4687	CARD	B	*macA*	95.93	100.00
Z4687	CARD	B	*macB*	97.31	100.00
Z4687	CARD	B	*mtrC*	97.50	100.00
Z4687	CARD	B	*mtrD*	98.47	100.00
Z4687	CARD	B	*mtrE*	94.38	99.93
Z4687	CARD	B	*mtrR*	97.63	100.00
Z4687	CARD	C	*farA*	99.10	100.00
Z4687	CARD	C	*farB*	97.18	100.00
Z4687	CARD	C	*macA*	95.93	100.00
Z4687	CARD	C	*macB*	97.31	100.00
Z4687	CARD	C	*mtrC*	97.50	100.00

Z4687	CARD	C	*mtrD*	98.47	100.00
Z4687	CARD	C	*mtrE*	94.38	99.93
Z4687	CARD	C	*mtrR*	97.63	100.00
Z4688	CARD	A	*farA*	98.30	100.00
Z4688	CARD	A	*farB*	96.27	100.00
Z4688	CARD	A	*macA*	95.93	100.00
Z4688	CARD	A	*macB*	97.05	100.00
Z4688	CARD	A	*mtrC*	98.07	100.00
Z4688	CARD	A	*mtrD*	99.06	100.00
Z4688	CARD	A	*mtrE*	94.24	99.93
Z4688	CARD	A	*mtrR*	97.63	100.00
Z4688	CARD	B	*farA*	98.30	100.00
Z4688	CARD	B	*farB*	96.27	100.00
Z4688	CARD	B	*macA*	95.93	100.00
Z4688	CARD	B	*macB*	97.05	100.00
Z4688	CARD	B	*mtrC*	98.07	100.00
Z4688	CARD	B	*mtrD*	99.06	100.00
Z4688	CARD	B	*mtrE*	94.24	99.93
Z4688	CARD	B	*mtrR*	97.63	100.00
Z4688	CARD	C	*farA*	98.30	100.00
Z4688	CARD	C	*farB*	96.27	100.00
Z4688	CARD	C	*macA*	95.93	100.00
Z4688	CARD	C	*macB*	97.05	100.00
Z4688	CARD	C	*mtrC*	98.07	100.00
Z4688	CARD	C	*mtrD*	99.06	100.00
Z4688	CARD	C	*mtrE*	94.24	99.93
Z4688	CARD	C	*mtrR*	97.63	100.00
Z4689	CARD	A	*farA*	98.62	100.00
Z4689	CARD	A	*farB*	97.77	100.00
Z4689	CARD	A	*macA*	96.27	100.00
Z4689	CARD	A	*macB*	96.49	100.00
Z4689	CARD	A	*mtrC*	97.10	100.00
Z4689	CARD	A	*mtrD*	98.91	100.00
Z4689	CARD	A	*mtrE*	94.52	99.93
Z4689	CARD	A	*mtrR*	97.48	100.00
Z4689	CARD	B	*farA*	98.62	100.00
Z4689	CARD	B	*farB*	97.77	100.00
Z4689	CARD	B	*macA*	96.27	100.00
Z4689	CARD	B	*macB*	96.49	100.00
Z4689	CARD	B	*mtrC*	97.10	100.00
Z4689	CARD	B	*mtrD*	98.91	100.00
Z4689	CARD	B	*mtrE*	94.52	99.93
Z4689	CARD	B	*mtrR*	97.48	100.00
Z4689	CARD	C	*farA*	98.62	100.00

Z4689	CARD	C	*farB*	97.77	100.00
Z4689	CARD	C	*macA*	96.27	100.00
Z4689	CARD	C	*macB*	96.49	100.00
Z4689	CARD	C	*mtrC*	97.10	100.00
Z4689	CARD	C	*mtrD*	98.91	100.00
Z4689	CARD	C	*mtrE*	94.52	99.93
Z4689	CARD	C	*mtrR*	97.48	100.00
Z4690	CARD	A	*farA*	99.10	100.00
Z4690	CARD	A	*farB*	95.48	100.00
Z4690	CARD	A	*macA*	96.10	100.00
Z4690	CARD	A	*macB*	97.42	100.00
Z4690	CARD	A	*mtrC*	97.26	100.00
Z4690	CARD	A	*mtrD*	98.78	100.00
Z4690	CARD	A	*mtrE*	94.52	99.93
Z4690	CARD	A	*mtrR*	97.63	100.00
Z4690	CARD	B	*farA*	99.10	100.00
Z4690	CARD	B	*farB*	95.48	100.00
Z4690	CARD	B	*macA*	96.10	100.00
Z4690	CARD	B	*macB*	97.42	100.00
Z4690	CARD	B	*mtrC*	97.26	100.00
Z4690	CARD	B	*mtrD*	98.78	100.00
Z4690	CARD	B	*mtrE*	94.52	99.93
Z4690	CARD	B	*mtrR*	97.63	100.00
Z4690	CARD	C	*farA*	99.10	100.00
Z4690	CARD	C	*farB*	95.48	100.00
Z4690	CARD	C	*macA*	96.10	100.00
Z4690	CARD	C	*macB*	97.42	100.00
Z4690	CARD	C	*mtrC*	97.26	100.00
Z4690	CARD	C	*mtrD*	98.78	100.00
Z4690	CARD	C	*mtrE*	94.52	99.93
Z4690	CARD	C	*mtrR*	97.63	100.00
Z4690	NDARO	C	*blaOXA*	99.86	85.00
Z4691	CARD	A	*farA*	99.10	100.00
Z4691	CARD	A	*farB*	95.48	100.00
Z4691	CARD	A	*macA*	96.10	100.00
Z4691	CARD	A	*macB*	97.42	100.00
Z4691	CARD	A	*mtrC*	97.58	100.00
Z4691	CARD	A	*mtrD*	98.91	100.00
Z4691	CARD	A	*mtrE*	94.52	99.93
Z4691	CARD	A	*mtrR*	97.31	100.00
Z4691	CARD	B	*farA*	99.10	100.00
Z4691	CARD	B	*farB*	95.48	100.00
Z4691	CARD	B	*macA*	96.10	100.00
Z4691	CARD	B	*macB*	97.42	100.00

Z4691	CARD	B	*mtrC*	97.58	100.00
Z4691	CARD	B	*mtrD*	98.91	100.00
Z4691	CARD	B	*mtrE*	94.52	99.93
Z4691	CARD	B	*mtrR*	97.31	100.00
Z4691	CARD	C	*farA*	99.10	100.00
Z4691	CARD	C	*farB*	95.48	100.00
Z4691	CARD	C	*macA*	96.10	100.00
Z4691	CARD	C	*macB*	97.42	100.00
Z4691	CARD	C	*mtrC*	97.58	100.00
Z4691	CARD	C	*mtrD*	98.91	100.00
Z4691	CARD	C	*mtrE*	94.52	99.93
Z4691	CARD	C	*mtrR*	97.31	100.00
Z4692	CARD	A	*farA*	99.13	100.00
Z4692	CARD	A	*farB*	97.32	100.00
Z4692	CARD	A	*macA*	95.93	100.00
Z4692	CARD	A	*macB*	96.64	100.00
Z4692	CARD	A	*mtrC*	96.61	100.00
Z4692	CARD	A	*mtrD*	98.81	100.00
Z4692	CARD	A	*mtrE*	94.52	99.93
Z4692	CARD	A	*mtrR*	97.95	100.00
Z4692	CARD	B	*farA*	99.13	100.00
Z4692	CARD	B	*farB*	97.32	100.00
Z4692	CARD	B	*macA*	95.93	100.00
Z4692	CARD	B	*macB*	96.64	100.00
Z4692	CARD	B	*mtrC*	96.61	100.00
Z4692	CARD	B	*mtrD*	98.81	100.00
Z4692	CARD	B	*mtrE*	94.52	99.93
Z4692	CARD	B	*mtrR*	97.95	100.00
Z4692	CARD	C	*farA*	99.13	100.00
Z4692	CARD	C	*farB*	97.32	100.00
Z4692	CARD	C	*macA*	95.93	100.00
Z4692	CARD	C	*macB*	96.64	100.00
Z4692	CARD	C	*mtrC*	96.61	100.00
Z4692	CARD	C	*mtrD*	98.81	100.00
Z4692	CARD	C	*mtrE*	94.52	99.93
Z4692	CARD	C	*mtrR*	97.95	100.00
Z4693	CARD	A	*farA*	99.13	100.00
Z4693	CARD	A	*farB*	97.32	100.00
Z4693	CARD	A	*macA*	96.10	100.00
Z4693	CARD	A	*macB*	97.31	100.00
Z4693	CARD	A	*mtrC*	96.61	100.00
Z4693	CARD	A	*mtrD*	98.81	100.00
Z4693	CARD	A	*mtrE*	94.52	99.93
Z4693	CARD	A	*mtrR*	97.95	100.00

Z4693	CARD	B	*farA*	99.13	100.00
Z4693	CARD	B	*farB*	97.32	100.00
Z4693	CARD	B	*macA*	96.10	100.00
Z4693	CARD	B	*macB*	97.31	100.00
Z4693	CARD	B	*mtrC*	96.61	100.00
Z4693	CARD	B	*mtrD*	98.81	100.00
Z4693	CARD	B	*mtrE*	94.52	99.93
Z4693	CARD	B	*mtrR*	97.95	100.00
Z4693	CARD	C	*farA*	99.13	100.00
Z4693	CARD	C	*farB*	97.32	100.00
Z4693	CARD	C	*macA*	96.10	100.00
Z4693	CARD	C	*macB*	97.31	100.00
Z4693	CARD	C	*mtrC*	96.61	100.00
Z4693	CARD	C	*mtrD*	98.81	100.00
Z4693	CARD	C	*mtrE*	94.52	99.93
Z4693	CARD	C	*mtrR*	97.95	100.00
Z4707	CARD	A	*farA*	99.01	100.00
Z4707	CARD	A	*farB*	96.73	100.00
Z4707	CARD	A	*macA*	95.93	100.00
Z4707	CARD	A	*macB*	97.47	100.00
Z4707	CARD	A	*mtrC*	98.07	100.00
Z4707	CARD	A	*mtrD*	99.06	100.00
Z4707	CARD	A	*mtrE*	94.81	99.93
Z4707	CARD	A	*mtrR*	97.63	100.00
Z4707	CARD	B	*farA*	99.01	100.00
Z4707	CARD	B	*farB*	96.73	100.00
Z4707	CARD	B	*macA*	95.93	100.00
Z4707	CARD	B	*macB*	97.47	100.00
Z4707	CARD	B	*mtrC*	98.07	100.00
Z4707	CARD	B	*mtrD*	99.06	100.00
Z4707	CARD	B	*mtrE*	94.81	99.93
Z4707	CARD	B	*mtrR*	97.63	100.00
Z4707	CARD	C	*farA*	99.01	100.00
Z4707	CARD	C	*farB*	96.73	100.00
Z4707	CARD	C	*macA*	95.93	100.00
Z4707	CARD	C	*macB*	97.47	100.00
Z4707	CARD	C	*mtrC*	98.07	100.00
Z4707	CARD	C	*mtrD*	99.06	100.00
Z4707	CARD	C	*mtrE*	94.81	99.93
Z4707	CARD	C	*mtrR*	97.63	100.00
Z4707	NDARO	B	*blaOXA*	100	62.38
Z4708	CARD	A	*farA*	99.48	100.00
Z4708	CARD	A	*farB*	97.64	100.00
Z4708	CARD	A	*macA*	95.93	100.00

Z4708	CARD	A	*macB*	96.85	100.00
Z4708	CARD	A	*mtrC*	97.34	100.00
Z4708	CARD	A	*mtrD*	98.94	100.00
Z4708	CARD	A	*mtrE*	94.45	99.93
Z4708	CARD	A	*mtrR*	97.95	100.00
Z4708	CARD	B	*farA*	99.48	100.00
Z4708	CARD	B	*farB*	97.64	100.00
Z4708	CARD	B	*macA*	95.93	100.00
Z4708	CARD	B	*macB*	96.85	100.00
Z4708	CARD	B	*mtrC*	97.34	100.00
Z4708	CARD	B	*mtrD*	98.94	100.00
Z4708	CARD	B	*mtrE*	94.45	99.93
Z4708	CARD	B	*mtrR*	97.95	100.00
Z4708	CARD	C	*farA*	99.48	100.00
Z4708	CARD	C	*farB*	97.64	100.00
Z4708	CARD	C	*macA*	95.93	100.00
Z4708	CARD	C	*macB*	96.85	100.00
Z4708	CARD	C	*mtrC*	97.34	100.00
Z4708	CARD	C	*mtrD*	98.94	100.00
Z4708	CARD	C	*mtrE*	94.45	99.93
Z4708	CARD	C	*mtrR*	97.95	100.00
Z4709	CARD	A	*farA*	99.19	100.00
Z4709	CARD	A	*farB*	95.42	100.00
Z4709	CARD	A	*macA*	96.18	100.00
Z4709	CARD	A	*macB*	97.42	100.00
Z4709	CARD	A	*mtrC*	98.07	100.00
Z4709	CARD	A	*mtrD*	99.16	100.00
Z4709	CARD	A	*mtrE*	94.38	99.93
Z4709	CARD	A	*mtrR*	97.79	100.00
Z4709	CARD	B	*farA*	99.19	100.00
Z4709	CARD	B	*farB*	95.42	100.00
Z4709	CARD	B	*macA*	96.18	100.00
Z4709	CARD	B	*macB*	97.42	100.00
Z4709	CARD	B	*mtrC*	98.07	100.00
Z4709	CARD	B	*mtrD*	99.16	100.00
Z4709	CARD	B	*mtrE*	94.38	99.93
Z4709	CARD	B	*mtrR*	97.79	100.00
Z4709	CARD	C	*farA*	99.19	100.00
Z4709	CARD	C	*farB*	95.42	100.00
Z4709	CARD	C	*macA*	96.18	100.00
Z4709	CARD	C	*macB*	97.42	100.00
Z4709	CARD	C	*mtrC*	98.07	100.00
Z4709	CARD	C	*mtrD*	99.16	100.00
Z4709	CARD	C	*mtrE*	94.38	99.93

Z4709	CARD	C	*mtrR*	97.79	100.00
Z4710	CARD	A	*farA*	98.92	100.00
Z4710	CARD	A	*farB*	98.04	100.00
Z4710	CARD	A	*macA*	96.52	100.00
Z4710	CARD	A	*macB*	97.52	100.00
Z4710	CARD	A	*mtrC*	98.07	100.00
Z4710	CARD	A	*mtrD*	98.50	100.00
Z4710	CARD	A	*mtrE*	94.38	99.93
Z4710	CARD	A	*mtrR*	97.48	100.00
Z4710	CARD	B	*farA*	98.92	100.00
Z4710	CARD	B	*farB*	98.04	100.00
Z4710	CARD	B	*macA*	96.52	100.00
Z4710	CARD	B	*macB*	97.52	100.00
Z4710	CARD	B	*mtrC*	98.07	100.00
Z4710	CARD	B	*mtrD*	98.50	100.00
Z4710	CARD	B	*mtrE*	94.38	99.93
Z4710	CARD	B	*mtrR*	97.48	100.00
Z4710	CARD	C	*farA*	98.92	100.00
Z4710	CARD	C	*farB*	98.04	100.00
Z4710	CARD	C	*macA*	96.52	100.00
Z4710	CARD	C	*macB*	97.52	100.00
Z4710	CARD	C	*mtrC*	98.07	100.00
Z4710	CARD	C	*mtrD*	98.50	100.00
Z4710	CARD	C	*mtrE*	94.38	99.93
Z4710	CARD	C	*mtrR*	97.48	100.00
Z4711	CARD	A	*farA*	98.92	100.00
Z4711	CARD	A	*farB*	98.04	100.00
Z4711	CARD	A	*macA*	96.10	100.00
Z4711	CARD	A	*macB*	97.47	100.00
Z4711	CARD	A	*mtrC*	97.58	100.00
Z4711	CARD	A	*mtrD*	98.97	100.00
Z4711	CARD	A	*mtrE*	94.52	99.93
Z4711	CARD	A	*mtrR*	97.48	100.00
Z4711	CARD	B	*farA*	98.92	100.00
Z4711	CARD	B	*farB*	98.04	100.00
Z4711	CARD	B	*macA*	96.10	100.00
Z4711	CARD	B	*macB*	97.47	100.00
Z4711	CARD	B	*mtrC*	97.58	100.00
Z4711	CARD	B	*mtrD*	98.97	100.00
Z4711	CARD	B	*mtrE*	94.52	99.93
Z4711	CARD	B	*mtrR*	97.48	100.00
Z4711	CARD	C	*farA*	98.92	100.00
Z4711	CARD	C	*farB*	98.04	100.00
Z4711	CARD	C	*macA*	96.10	100.00

Z4711	CARD	C	*macB*	97.47	100.00
Z4711	CARD	C	*mtrC*	97.58	100.00
Z4711	CARD	C	*mtrD*	98.97	100.00
Z4711	CARD	C	*mtrE*	94.52	99.93
Z4711	CARD	C	*mtrR*	97.48	100.00
Z4756	CARD	A	*farA*	100.00	100.00
Z4756	CARD	A	*farB*	97.77	100.00
Z4756	CARD	A	*macA*	96.10	100.00
Z4756	CARD	A	*macB*	97.36	100.00
Z4756	CARD	A	*mtrC*	97.34	100.00
Z4756	CARD	A	*mtrD*	99.19	100.00
Z4756	CARD	A	*mtrE*	93.67	99.93
Z4756	CARD	A	*mtrR*	97.79	100.00
Z4756	CARD	B	*farA*	100.00	100.00
Z4756	CARD	B	*farB*	97.77	100.00
Z4756	CARD	B	*macA*	96.10	100.00
Z4756	CARD	B	*macB*	97.36	100.00
Z4756	CARD	B	*mtrC*	97.34	100.00
Z4756	CARD	B	*mtrD*	99.19	100.00
Z4756	CARD	B	*mtrE*	93.67	99.93
Z4756	CARD	B	*mtrR*	97.79	100.00
Z4756	CARD	C	*farA*	100.00	100.00
Z4756	CARD	C	*farB*	97.77	100.00
Z4756	CARD	C	*macA*	96.10	100.00
Z4756	CARD	C	*macB*	97.36	100.00
Z4756	CARD	C	*mtrC*	97.34	100.00
Z4756	CARD	C	*mtrD*	99.19	100.00
Z4756	CARD	C	*mtrE*	93.67	99.93
Z4756	CARD	C	*mtrR*	97.79	100.00
Z4765	CARD	A	*farA*	99.19	100.00
Z4765	CARD	A	*farB*	97.84	100.00
Z4765	CARD	A	*macA*	95.59	100.00
Z4765	CARD	A	*macB*	96.95	100.00
Z4765	CARD	A	*mtrC*	97.50	100.00
Z4765	CARD	A	*mtrD*	99.10	100.00
Z4765	CARD	A	*mtrE*	94.31	99.93
Z4765	CARD	A	*mtrR*	97.63	100.00
Z4765	CARD	B	*farA*	99.19	100.00
Z4765	CARD	B	*farB*	97.84	100.00
Z4765	CARD	B	*macA*	95.59	100.00
Z4765	CARD	B	*macB*	96.95	100.00
Z4765	CARD	B	*mtrC*	97.50	100.00
Z4765	CARD	B	*mtrD*	99.10	100.00
Z4765	CARD	B	*mtrE*	94.31	99.93

Z4765	CARD	B	*mtrR*	97.63	100.00
Z4765	CARD	C	*farA*	99.19	100.00
Z4765	CARD	C	*farB*	97.84	100.00
Z4765	CARD	C	*macA*	95.59	100.00
Z4765	CARD	C	*macB*	96.95	100.00
Z4765	CARD	C	*mtrC*	97.50	100.00
Z4765	CARD	C	*mtrD*	99.10	100.00
Z4765	CARD	C	*mtrE*	94.31	99.93
Z4765	CARD	C	*mtrR*	97.63	100.00
Z5005	CARD	A	*farA*	100.00	100.00
Z5005	CARD	A	*farB*	97.77	100.00
Z5005	CARD	A	*macA*	96.10	100.00
Z5005	CARD	A	*macB*	97.36	100.00
Z5005	CARD	A	*mtrC*	97.34	100.00
Z5005	CARD	A	*mtrD*	99.19	100.00
Z5005	CARD	A	*mtrE*	93.67	99.93
Z5005	CARD	A	*mtrR*	97.79	100.00
Z5005	CARD	B	*farA*	100.00	100.00
Z5005	CARD	B	*farB*	97.77	100.00
Z5005	CARD	B	*macA*	96.10	100.00
Z5005	CARD	B	*macB*	97.36	100.00
Z5005	CARD	B	*mtrC*	97.34	100.00
Z5005	CARD	B	*mtrD*	99.19	100.00
Z5005	CARD	B	*mtrE*	93.67	99.93
Z5005	CARD	B	*mtrR*	97.79	100.00
Z5005	CARD	C	*farA*	100.00	100.00
Z5005	CARD	C	*farB*	97.77	100.00
Z5005	CARD	C	*macA*	96.10	100.00
Z5005	CARD	C	*macB*	97.36	100.00
Z5005	CARD	C	*mtrC*	97.34	100.00
Z5005	CARD	C	*mtrD*	99.19	100.00
Z5005	CARD	C	*mtrE*	93.67	99.93
Z5005	CARD	C	*mtrR*	97.79	100.00
Z5035	CARD	A	*farA*	100.00	100.00
Z5035	CARD	A	*farB*	97.77	100.00
Z5035	CARD	A	*macA*	96.78	100.00
Z5035	CARD	A	*macB*	97.36	100.00
Z5035	CARD	A	*mtrC*	97.50	100.00
Z5035	CARD	A	*mtrD*	99.13	100.00
Z5035	CARD	A	*mtrE*	94.60	99.93
Z5035	CARD	A	*mtrR*	97.63	100.00
Z5035	CARD	B	*farA*	100.00	100.00
Z5035	CARD	B	*farB*	97.77	100.00
Z5035	CARD	B	*macA*	96.78	100.00

Z5035	CARD	B	*macB*	97.36	100.00
Z5035	CARD	B	*mtrC*	97.50	100.00
Z5035	CARD	B	*mtrD*	99.13	100.00
Z5035	CARD	B	*mtrE*	94.60	99.93
Z5035	CARD	B	*mtrR*	97.63	100.00
Z5035	CARD	C	*farA*	100.00	100.00
Z5035	CARD	C	*farB*	97.77	100.00
Z5035	CARD	C	*macA*	96.78	100.00
Z5035	CARD	C	*macB*	97.36	100.00
Z5035	CARD	C	*mtrC*	97.50	100.00
Z5035	CARD	C	*mtrD*	99.13	100.00
Z5035	CARD	C	*mtrE*	94.60	99.93
Z5035	CARD	C	*mtrR*	97.63	100.00
Z5037	CARD	A	*farA*	99.57	100.00
Z5037	CARD	A	*farB*	98.89	100.00
Z5037	CARD	A	*macA*	95.84	100.00
Z5037	CARD	A	*macB*	97.36	100.00
Z5037	CARD	A	*mtrC*	97.34	100.00
Z5037	CARD	A	*mtrD*	99.03	100.00
Z5037	CARD	A	*mtrE*	94.24	99.93
Z5037	CARD	A	*mtrR*	97.79	100.00
Z5037	CARD	B	*farA*	99.57	100.00
Z5037	CARD	B	*farB*	98.89	100.00
Z5037	CARD	B	*macA*	95.84	100.00
Z5037	CARD	B	*macB*	97.36	100.00
Z5037	CARD	B	*mtrC*	97.34	100.00
Z5037	CARD	B	*mtrD*	99.03	100.00
Z5037	CARD	B	*mtrE*	94.24	99.93
Z5037	CARD	B	*mtrR*	97.79	100.00
Z5037	CARD	C	*farA*	99.57	100.00
Z5037	CARD	C	*farB*	98.89	100.00
Z5037	CARD	C	*macA*	95.84	100.00
Z5037	CARD	C	*macB*	97.36	100.00
Z5037	CARD	C	*mtrC*	97.34	100.00
Z5037	CARD	C	*mtrD*	99.03	100.00
Z5037	CARD	C	*mtrE*	94.24	99.93
Z5037	CARD	C	*mtrR*	97.79	100.00
Z5043	CARD	A	*farA*	100.00	100.00
Z5043	CARD	A	*farB*	97.77	100.00
Z5043	CARD	A	*macA*	96.27	100.00
Z5043	CARD	A	*macB*	97.05	100.00
Z5043	CARD	A	*mtrC*	97.50	100.00
Z5043	CARD	A	*mtrD*	99.13	100.00
Z5043	CARD	A	*mtrE*	94.60	99.93

Z5043	CARD	A	*mtrR*	97.63	100.00
Z5043	CARD	B	*farA*	100.00	100.00
Z5043	CARD	B	*farB*	97.77	100.00
Z5043	CARD	B	*macA*	96.27	100.00
Z5043	CARD	B	*macB*	97.05	100.00
Z5043	CARD	B	*mtrC*	97.50	100.00
Z5043	CARD	B	*mtrD*	99.13	100.00
Z5043	CARD	B	*mtrE*	94.60	99.93
Z5043	CARD	B	*mtrR*	97.63	100.00
Z5043	CARD	C	*farA*	100.00	100.00
Z5043	CARD	C	*farB*	97.77	100.00
Z5043	CARD	C	*macA*	96.27	100.00
Z5043	CARD	C	*macB*	97.05	100.00
Z5043	CARD	C	*mtrC*	97.50	100.00
Z5043	CARD	C	*mtrD*	99.13	100.00
Z5043	CARD	C	*mtrE*	94.60	99.93
Z5043	CARD	C	*mtrR*	97.63	100.00
Z5043	NDARO	C	*blaOXA*	99.82	64.76
Z5826	CARD	A	*farA*	99.91	100.00
Z5826	CARD	A	*farB*	97.77	100.00
Z5826	CARD	A	*macA*	96.10	100.00
Z5826	CARD	A	*macB*	96.59	100.00
Z5826	CARD	A	*mtrC*	97.02	100.00
Z5826	CARD	A	*mtrD*	99.19	100.00
Z5826	CARD	A	*mtrE*	93.74	99.93
Z5826	CARD	A	*mtrR*	97.48	100.00
Z5826	CARD	A	*tetA*	99.92	100.00
Z5826	ResFinder	A	*tetA*	99.92	100.00
Z5826	ARG-ANNOT	A	*tetA*	99.67	100.00
Z5826	NDARO	A	*tet(B)*	99.93	100.00
Z5826	CARD	B	*farA*	99.91	100.00
Z5826	CARD	B	*farB*	97.77	100.00
Z5826	CARD	B	*macA*	96.10	100.00
Z5826	CARD	B	*macB*	96.59	100.00
Z5826	CARD	B	*mtrC*	97.02	100.00
Z5826	CARD	B	*mtrD*	99.19	100.00
Z5826	CARD	B	*mtrE*	93.74	99.93
Z5826	CARD	B	*mtrR*	97.48	100.00
Z5826	CARD	B	*tetA*	99.92	100.00
Z5826	ResFinder	B	*tetA*	99.92	100.00
Z5826	ARG-ANNOT	B	*tetA*	99.67	100.00
Z5826	NDARO	B	*tet(B)*	99.93	100.00
Z5826	CARD	C	*farA*	99.91	100.00
Z5826	CARD	C	*farB*	97.77	100.00

Z5826	CARD	C	*macA*	96.10	100.00
Z5826	CARD	C	*macB*	96.59	100.00
Z5826	CARD	C	*mtrC*	97.02	100.00
Z5826	CARD	C	*mtrD*	99.19	100.00
Z5826	CARD	C	*mtrE*	93.74	99.93
Z5826	CARD	C	*mtrR*	97.48	100.00
Z5826	CARD	C	*tetA*	99.92	100.00
Z5826	ResFinder	C	*tetA*	99.92	100.00
Z5826	ARG-ANNOT	C	*tetA*	99.67	100.00
Z5826	NDARO	C	*tet(B)*	99.93	100.00
Z6412	CARD	A	*farA*	99.64	100.00
Z6412	CARD	A	*farB*	95.02	100.00
Z6412	CARD	A	*macA*	96.27	100.00
Z6412	CARD	A	*macB*	96.85	100.00
Z6412	CARD	A	*mtrC*	97.18	100.00
Z6412	CARD	A	*mtrD*	99.03	100.00
Z6412	CARD	A	*mtrE*	94.31	99.93
Z6412	CARD	A	*mtrR*	97.63	100.00
Z6412	CARD	B	*farA*	99.64	100.00
Z6412	CARD	B	*farB*	95.02	100.00
Z6412	CARD	B	*macA*	96.27	100.00
Z6412	CARD	B	*macB*	96.85	100.00
Z6412	CARD	B	*mtrC*	97.18	100.00
Z6412	CARD	B	*mtrD*	99.03	100.00
Z6412	CARD	B	*mtrE*	94.31	99.93
Z6412	CARD	B	*mtrR*	97.63	100.00
Z6412	CARD	C	*farA*	99.64	100.00
Z6412	CARD	C	*farB*	95.02	100.00
Z6412	CARD	C	*macA*	96.27	100.00
Z6412	CARD	C	*macB*	96.85	100.00
Z6412	CARD	C	*mtrC*	97.18	100.00
Z6412	CARD	C	*mtrD*	99.03	100.00
Z6412	CARD	C	*mtrE*	94.31	99.93
Z6412	CARD	C	*mtrR*	97.63	100.00
Z6414	CARD	A	*farA*	99.64	100.00
Z6414	CARD	A	*farB*	95.02	100.00
Z6414	CARD	A	*macA*	96.18	100.00
Z6414	CARD	A	*macB*	97.42	100.00
Z6414	CARD	A	*mtrC*	97.34	100.00
Z6414	CARD	A	*mtrD*	99.16	100.00
Z6414	CARD	A	*mtrE*	94.52	99.93
Z6414	CARD	A	*mtrR*	97.63	100.00
Z6414	CARD	B	*farA*	99.64	100.00
Z6414	CARD	B	*farB*	95.02	100.00

Z6414	CARD	B	*macA*	96.18	100.00
Z6414	CARD	B	*macB*	97.42	100.00
Z6414	CARD	B	*mtrC*	97.34	100.00
Z6414	CARD	B	*mtrD*	99.16	100.00
Z6414	CARD	B	*mtrE*	94.52	99.93
Z6414	CARD	B	*mtrR*	97.63	100.00
Z6414	NDARO	B	*blaOXA*	100	65.95
Z6414	CARD	C	*farA*	99.64	100.00
Z6414	CARD	C	*farB*	95.02	100.00
Z6414	CARD	C	*macA*	96.18	100.00
Z6414	CARD	C	*macB*	97.42	100.00
Z6414	CARD	C	*mtrC*	97.34	100.00
Z6414	CARD	C	*mtrD*	99.16	100.00
Z6414	CARD	C	*mtrE*	94.52	99.93
Z6414	CARD	C	*mtrR*	97.63	100.00
Z6415	CARD	A	*farA*	99.64	100.00
Z6415	CARD	A	*farB*	95.02	100.00
Z6415	CARD	A	*macA*	96.18	100.00
Z6415	CARD	A	*macB*	97.42	100.00
Z6415	CARD	A	*mtrC*	97.34	100.00
Z6415	CARD	A	*mtrD*	99.25	100.00
Z6415	CARD	A	*mtrE*	94.31	99.93
Z6415	CARD	A	*mtrR*	97.48	100.00
Z6415	CARD	B	*farA*	99.64	100.00
Z6415	CARD	B	*farB*	95.02	100.00
Z6415	CARD	B	*macA*	96.18	100.00
Z6415	CARD	B	*macB*	97.42	100.00
Z6415	CARD	B	*mtrC*	97.34	100.00
Z6415	CARD	B	*mtrD*	99.25	100.00
Z6415	CARD	B	*mtrE*	94.31	99.93
Z6415	CARD	B	*mtrR*	97.48	100.00
Z6415	CARD	C	*farA*	99.64	100.00
Z6415	CARD	C	*farB*	95.02	100.00
Z6415	CARD	C	*macA*	96.18	100.00
Z6415	CARD	C	*macB*	97.42	100.00
Z6415	CARD	C	*mtrC*	97.34	100.00
Z6415	CARD	C	*mtrD*	99.25	100.00
Z6415	CARD	C	*mtrE*	94.31	99.93
Z6415	CARD	C	*mtrR*	97.48	100.00
Z6416	CARD	A	*farA*	99.64	100.00
Z6416	CARD	A	*farB*	95.02	100.00
Z6416	CARD	A	*macA*	96.27	100.00
Z6416	CARD	A	*macB*	97.42	100.00
Z6416	CARD	A	*mtrC*	97.34	100.00

Z6416	CARD	A	*mtrD*	99.25	100.00
Z6416	CARD	A	*mtrE*	94.31	99.93
Z6416	CARD	A	*mtrR*	97.32	100.00
Z6416	CARD	B	*farA*	99.64	100.00
Z6416	CARD	B	*farB*	95.02	100.00
Z6416	CARD	B	*macA*	96.27	100.00
Z6416	CARD	B	*macB*	97.42	100.00
Z6416	CARD	B	*mtrC*	97.34	100.00
Z6416	CARD	B	*mtrD*	99.25	100.00
Z6416	CARD	B	*mtrE*	94.31	99.93
Z6416	CARD	B	*mtrR*	97.32	100.00
Z6416	CARD	C	*farA*	99.64	100.00
Z6416	CARD	C	*farB*	95.02	100.00
Z6416	CARD	C	*macA*	96.27	100.00
Z6416	CARD	C	*macB*	97.42	100.00
Z6416	CARD	C	*mtrC*	97.34	100.00
Z6416	CARD	C	*mtrD*	99.25	100.00
Z6416	CARD	C	*mtrE*	94.31	99.93
Z6416	CARD	C	*mtrR*	97.32	100.00
Z6418	CARD	A	*farA*	100.00	100.00
Z6418	CARD	A	*farB*	100.00	100.00
Z6418	CARD	A	*macA*	96.10	100.00
Z6418	CARD	A	*macB*	97.36	100.00
Z6418	CARD	A	*mtrC*	100.00	100.00
Z6418	CARD	A	*mtrD*	100.00	100.00
Z6418	CARD	A	*mtrE*	94.52	99.93
Z6418	CARD	A	*mtrR*	97.48	100.00
Z6418	CARD	B	*farA*	100.00	100.00
Z6418	CARD	B	*farB*	100.00	100.00
Z6418	CARD	B	*macA*	96.10	100.00
Z6418	CARD	B	*macB*	97.36	100.00
Z6418	CARD	B	*mtrC*	100.00	100.00
Z6418	CARD	B	*mtrD*	100.00	100.00
Z6418	CARD	B	*mtrE*	94.52	99.93
Z6418	CARD	B	*mtrR*	97.48	100.00
Z6418	CARD	C	*farA*	100.00	100.00
Z6418	CARD	C	*farB*	100.00	100.00
Z6418	CARD	C	*macA*	96.10	100.00
Z6418	CARD	C	*macB*	97.36	100.00
Z6418	CARD	C	*mtrC*	100.00	100.00
Z6418	CARD	C	*mtrD*	100.00	100.00
Z6418	CARD	C	*mtrE*	94.52	99.93
Z6418	CARD	C	*mtrR*	97.48	100.00
Z6419	CARD	A	*farA*	98.87	100.00

Z6419	CARD	A	*farB*	96.79	100.00
Z6419	CARD	A	*macA*	95.67	100.00
Z6419	CARD	A	*macB*	96.95	100.00
Z6419	CARD	A	*mtrC*	97.26	100.00
Z6419	CARD	A	*mtrD*	98.78	100.00
Z6419	CARD	A	*mtrE*	94.74	99.93
Z6419	CARD	A	*mtrR*	97.95	100.00
Z6419	CARD	B	*farA*	98.87	100.00
Z6419	CARD	B	*farB*	96.79	100.00
Z6419	CARD	B	*macA*	95.67	100.00
Z6419	CARD	B	*macB*	96.95	100.00
Z6419	CARD	B	*mtrC*	97.26	100.00
Z6419	CARD	B	*mtrD*	98.78	100.00
Z6419	CARD	B	*mtrE*	94.74	99.93
Z6419	CARD	B	*mtrR*	97.95	100.00
Z6419	CARD	C	*farA*	98.87	100.00
Z6419	CARD	C	*farB*	96.79	100.00
Z6419	CARD	C	*macA*	95.67	100.00
Z6419	CARD	C	*macB*	96.95	100.00
Z6419	CARD	C	*mtrC*	97.26	100.00
Z6419	CARD	C	*mtrD*	98.78	100.00
Z6419	CARD	C	*mtrE*	94.74	99.93
Z6419	CARD	C	*mtrR*	97.95	100.00
Z6422	CARD	A	*farA*	98.87	100.00
Z6422	CARD	A	*farB*	96.79	100.00
Z6422	CARD	A	*macA*	95.84	100.00
Z6422	CARD	A	*macB*	97.11	100.00
Z6422	CARD	A	*mtrC*	97.26	100.00
Z6422	CARD	A	*mtrD*	98.78	100.00
Z6422	CARD	A	*mtrE*	94.52	99.93
Z6422	CARD	A	*mtrR*	97.95	100.00
Z6422	CARD	B	*farA*	98.87	100.00
Z6422	CARD	B	*farB*	96.79	100.00
Z6422	CARD	B	*macA*	95.84	100.00
Z6422	CARD	B	*macB*	97.11	100.00
Z6422	CARD	B	*mtrC*	97.26	100.00
Z6422	CARD	B	*mtrD*	98.78	100.00
Z6422	CARD	B	*mtrE*	94.52	99.93
Z6422	CARD	B	*mtrR*	97.95	100.00
Z6422	CARD	C	*farA*	98.87	100.00
Z6422	CARD	C	*farB*	96.79	100.00
Z6422	CARD	C	*macA*	95.84	100.00
Z6422	CARD	C	*macB*	97.11	100.00
Z6422	CARD	C	*mtrC*	97.26	100.00

Z6422	CARD	C	*mtrD*	98.78	100.00
Z6422	CARD	C	*mtrE*	94.52	99.93
Z6422	CARD	C	*mtrR*	97.95	100.00
Z6426	CARD	A	*farA*	98.87	100.00
Z6426	CARD	A	*farB*	96.79	100.00
Z6426	CARD	A	*macA*	95.67	100.00
Z6426	CARD	A	*macB*	96.95	100.00
Z6426	CARD	A	*mtrC*	97.26	100.00
Z6426	CARD	A	*mtrD*	98.78	100.00
Z6426	CARD	A	*mtrE*	94.52	99.93
Z6426	CARD	A	*mtrR*	97.95	100.00
Z6426	CARD	B	*farA*	98.87	100.00
Z6426	CARD	B	*farB*	96.79	100.00
Z6426	CARD	B	*macA*	95.67	100.00
Z6426	CARD	B	*macB*	96.95	100.00
Z6426	CARD	B	*mtrC*	97.26	100.00
Z6426	CARD	B	*mtrD*	98.78	100.00
Z6426	CARD	B	*mtrE*	94.52	99.93
Z6426	CARD	B	*mtrR*	97.95	100.00
Z6426	CARD	C	*farA*	98.87	100.00
Z6426	CARD	C	*farB*	96.79	100.00
Z6426	CARD	C	*macA*	95.67	100.00
Z6426	CARD	C	*macB*	96.95	100.00
Z6426	CARD	C	*mtrC*	97.26	100.00
Z6426	CARD	C	*mtrD*	98.78	100.00
Z6426	CARD	C	*mtrE*	94.52	99.93
Z6426	CARD	C	*mtrR*	97.95	100.00
Z6427	CARD	A	*farA*	98.87	100.00
Z6427	CARD	A	*farB*	96.79	100.00
Z6427	CARD	A	*macA*	95.67	100.00
Z6427	CARD	A	*macB*	96.95	100.00
Z6427	CARD	A	*mtrC*	97.26	100.00
Z6427	CARD	A	*mtrD*	98.78	100.00
Z6427	CARD	A	*mtrE*	94.52	99.93
Z6427	CARD	A	*mtrR*	97.95	100.00
Z6427	CARD	B	*farA*	98.87	100.00
Z6427	CARD	B	*farB*	96.79	100.00
Z6427	CARD	B	*macA*	95.67	100.00
Z6427	CARD	B	*macB*	96.95	100.00
Z6427	CARD	B	*mtrC*	97.26	100.00
Z6427	CARD	B	*mtrD*	98.78	100.00
Z6427	CARD	B	*mtrE*	94.52	99.93
Z6427	CARD	B	*mtrR*	97.95	100.00
Z6427	CARD	C	*farA*	98.87	100.00

Z6427	CARD	C	*farB*	96.79	100.00
Z6427	CARD	C	*macA*	95.67	100.00
Z6427	CARD	C	*macB*	96.95	100.00
Z6427	CARD	C	*mtrC*	97.26	100.00
Z6427	CARD	C	*mtrD*	98.78	100.00
Z6427	CARD	C	*mtrE*	94.52	99.93
Z6427	CARD	C	*mtrR*	97.95	100.00
Z6428	CARD	A	*farA*	95.07	100.00
Z6428	CARD	A	*farB*	96.66	100.00
Z6428	CARD	A	*macA*	96.10	100.00
Z6428	CARD	A	*macB*	97.88	100.00
Z6428	CARD	A	*mtrC*	96.61	100.00
Z6428	CARD	A	*mtrD*	99.25	100.00
Z6428	CARD	A	*mtrE*	94.52	99.93
Z6428	CARD	A	*mtrR*	97.95	100.00
Z6428	CARD	B	*farA*	95.07	100.00
Z6428	CARD	B	*farB*	96.66	100.00
Z6428	CARD	B	*macA*	96.10	100.00
Z6428	CARD	B	*macB*	97.88	100.00
Z6428	CARD	B	*mtrC*	96.61	100.00
Z6428	CARD	B	*mtrD*	99.25	100.00
Z6428	CARD	B	*mtrE*	94.52	99.93
Z6428	CARD	B	*mtrR*	97.95	100.00
Z6428	CARD	C	*farA*	95.07	100.00
Z6428	CARD	C	*farB*	96.66	100.00
Z6428	CARD	C	*macA*	96.10	100.00
Z6428	CARD	C	*macB*	97.88	100.00
Z6428	CARD	C	*mtrC*	96.61	100.00
Z6428	CARD	C	*mtrD*	99.25	100.00
Z6428	CARD	C	*mtrE*	94.52	99.93
Z6428	CARD	C	*mtrR*	97.95	100.00
Z6429	CARD	A	*farA*	99.05	100.00
Z6429	CARD	A	*farB*	96.60	100.00
Z6429	CARD	A	*macA*	96.18	100.00
Z6429	CARD	A	*macB*	97.05	100.00
Z6429	CARD	A	*mtrC*	97.10	100.00
Z6429	CARD	A	*mtrD*	99.10	100.00
Z6429	CARD	A	*mtrE*	94.74	99.93
Z6429	CARD	A	*mtrR*	97.48	100.00
Z6429	CARD	B	*farA*	99.05	100.00
Z6429	CARD	B	*farB*	96.60	100.00
Z6429	CARD	B	*macA*	96.18	100.00
Z6429	CARD	B	*macB*	97.05	100.00
Z6429	CARD	B	*mtrC*	97.10	100.00

Z6429	CARD	B	*mtrD*	99.10	100.00
Z6429	CARD	B	*mtrE*	94.74	99.93
Z6429	CARD	B	*mtrR*	97.48	100.00
Z6429	CARD	C	*farA*	99.05	100.00
Z6429	CARD	C	*farB*	96.60	100.00
Z6429	CARD	C	*macA*	96.18	100.00
Z6429	CARD	C	*macB*	97.05	100.00
Z6429	CARD	C	*mtrC*	97.10	100.00
Z6429	CARD	C	*mtrD*	99.10	100.00
Z6429	CARD	C	*mtrE*	94.74	99.93
Z6429	CARD	C	*mtrR*	97.48	100.00
Z6430	CARD	A	*farA*	97.40	100.00
Z6430	CARD	A	*farB*	95.87	100.00
Z6430	CARD	A	*macA*	96.52	100.00
Z6430	CARD	A	*macB*	97.05	100.00
Z6430	CARD	A	*mtrC*	97.90	100.00
Z6430	CARD	A	*mtrD*	99.03	100.00
Z6430	CARD	A	*mtrE*	94.52	99.93
Z6430	CARD	A	*mtrR*	97.79	100.00
Z6430	CARD	B	*farA*	97.40	100.00
Z6430	CARD	B	*farB*	95.87	100.00
Z6430	CARD	B	*macA*	96.52	100.00
Z6430	CARD	B	*macB*	97.05	100.00
Z6430	CARD	B	*mtrC*	97.90	100.00
Z6430	CARD	B	*mtrD*	99.03	100.00
Z6430	CARD	B	*mtrE*	94.52	99.93
Z6430	CARD	B	*mtrR*	97.79	100.00
Z6430	NDARO	B	*blaOXA*	99.8	61.03
Z6430	CARD	C	*farA*	97.40	100.00
Z6430	CARD	C	*farB*	95.87	100.00
Z6430	CARD	C	*macA*	96.52	100.00
Z6430	CARD	C	*macB*	97.05	100.00
Z6430	CARD	C	*mtrC*	97.90	100.00
Z6430	CARD	C	*mtrD*	99.03	100.00
Z6430	CARD	C	*mtrE*	94.52	99.93
Z6430	CARD	C	*mtrR*	97.79	100.00
Z6431	CARD	A	*farA*	100.00	100.00
Z6431	CARD	A	*farB*	99.08	100.00
Z6431	CARD	A	*macA*	96.61	100.00
Z6431	CARD	A	*macB*	97.42	100.00
Z6431	CARD	A	*mtrC*	97.50	100.00
Z6431	CARD	A	*mtrD*	98.63	100.00
Z6431	CARD	A	*mtrE*	94.38	99.93
Z6431	CARD	A	*mtrR*	97.63	100.00

Z6431	CARD	B	*farA*	100.00	100.00
Z6431	CARD	B	*farB*	99.08	100.00
Z6431	CARD	B	*macA*	96.61	100.00
Z6431	CARD	B	*macB*	97.42	100.00
Z6431	CARD	B	*mtrC*	97.50	100.00
Z6431	CARD	B	*mtrD*	98.63	100.00
Z6431	CARD	B	*mtrE*	94.38	99.93
Z6431	CARD	B	*mtrR*	97.63	100.00
Z6431	CARD	C	*farA*	100.00	100.00
Z6431	CARD	C	*farB*	99.08	100.00
Z6431	CARD	C	*macA*	96.61	100.00
Z6431	CARD	C	*macB*	97.42	100.00
Z6431	CARD	C	*mtrC*	97.50	100.00
Z6431	CARD	C	*mtrD*	98.63	100.00
Z6431	CARD	C	*mtrE*	94.38	99.93
Z6431	CARD	C	*mtrR*	97.63	100.00
Z6432	CARD	A	*farA*	98.96	100.00
Z6432	CARD	A	*farB*	96.27	100.00
Z6432	CARD	A	*macA*	96.61	100.00
Z6432	CARD	A	*macB*	97.05	100.00
Z6432	CARD	A	*mtrC*	98.07	100.00
Z6432	CARD	A	*mtrD*	99.19	100.00
Z6432	CARD	A	*mtrE*	94.24	99.93
Z6432	CARD	A	*mtrR*	97.48	100.00
Z6432	CARD	B	*farA*	98.96	100.00
Z6432	CARD	B	*farB*	96.27	100.00
Z6432	CARD	B	*macA*	96.61	100.00
Z6432	CARD	B	*macB*	97.05	100.00
Z6432	CARD	B	*mtrC*	98.07	100.00
Z6432	CARD	B	*mtrD*	99.19	100.00
Z6432	CARD	B	*mtrE*	94.24	99.93
Z6432	CARD	B	*mtrR*	97.48	100.00
Z6432	CARD	C	*farA*	98.96	100.00
Z6432	CARD	C	*farB*	96.27	100.00
Z6432	CARD	C	*macA*	96.61	100.00
Z6432	CARD	C	*macB*	97.05	100.00
Z6432	CARD	C	*mtrC*	98.07	100.00
Z6432	CARD	C	*mtrD*	99.19	100.00
Z6432	CARD	C	*mtrE*	94.24	99.93
Z6432	CARD	C	*mtrR*	97.48	100.00
Z6433	CARD	A	*farA*	99.39	100.00
Z6433	CARD	A	*farB*	95.48	100.00
Z6433	CARD	A	*macA*	96.52	100.00
Z6433	CARD	A	*macB*	97.00	100.00

Z6433	CARD	A	*mtrC*	98.15	100.00
Z6433	CARD	A	*mtrD*	99.16	100.00
Z6433	CARD	A	*mtrE*	93.88	99.93
Z6433	CARD	A	*mtrR*	97.63	100.00
Z6433	CARD	B	*farA*	99.39	100.00
Z6433	CARD	B	*farB*	95.48	100.00
Z6433	CARD	B	*macA*	96.52	100.00
Z6433	CARD	B	*macB*	97.00	100.00
Z6433	CARD	B	*mtrC*	98.15	100.00
Z6433	CARD	B	*mtrD*	99.16	100.00
Z6433	CARD	B	*mtrE*	93.88	99.93
Z6433	CARD	B	*mtrR*	97.63	100.00
Z6433	CARD	C	*farA*	99.39	100.00
Z6433	CARD	C	*farB*	95.48	100.00
Z6433	CARD	C	*macA*	96.52	100.00
Z6433	CARD	C	*macB*	97.00	100.00
Z6433	CARD	C	*mtrC*	98.15	100.00
Z6433	CARD	C	*mtrD*	99.16	100.00
Z6433	CARD	C	*mtrE*	93.88	99.93
Z6433	CARD	C	*mtrR*	97.63	100.00
Z6434	CARD	A	*farA*	99.13	100.00
Z6434	CARD	A	*farB*	98.69	100.00
Z6434	CARD	A	*macA*	96.10	100.00
Z6434	CARD	A	*macB*	96.95	100.00
Z6434	CARD	A	*mtrC*	98.15	100.00
Z6434	CARD	A	*mtrD*	99.16	100.00
Z6434	CARD	A	*mtrE*	94.10	99.93
Z6434	CARD	A	*mtrR*	97.63	100.00
Z6434	CARD	B	*farA*	99.13	100.00
Z6434	CARD	B	*farB*	98.69	100.00
Z6434	CARD	B	*macA*	96.10	100.00
Z6434	CARD	B	*macB*	96.95	100.00
Z6434	CARD	B	*mtrC*	98.15	100.00
Z6434	CARD	B	*mtrD*	99.16	100.00
Z6434	CARD	B	*mtrE*	94.10	99.93
Z6434	CARD	B	*mtrR*	97.63	100.00
Z6434	CARD	C	*farA*	99.13	100.00
Z6434	CARD	C	*farB*	98.69	100.00
Z6434	CARD	C	*macA*	96.10	100.00
Z6434	CARD	C	*macB*	96.95	100.00
Z6434	CARD	C	*mtrC*	98.15	100.00
Z6434	CARD	C	*mtrD*	99.16	100.00
Z6434	CARD	C	*mtrE*	94.10	99.93
Z6434	CARD	C	*mtrR*	97.63	100.00

2.2.6 Table S9

Table S9: Overview of reproducibility of the sequence typing assay for the core validation dataset. The first column lists the sample name, while the second, third and fourth columns list the fraction of concordant cgMLST loci for run A versus B, A versus C, and B versus C, respectively. See also Figure 2 for a simplified overview.

Sample	Concordant loci run A versus B (%)	Concordant loci run A versus C (%)	Concordant loci run B versus C (%)
Z1001	99.81	99.81	100.00
Z1035	99.69	99.75	99.94
Z1054	99.44	99.56	99.81
Z1073	99.75	99.75	99.81
Z1092	99.69	99.75	99.94
Z1099	99.50	99.44	99.94
Z1269	99.44	99.25	99.50
Z1275	99.75	99.75	99.69
Z1392	99.56	99.50	99.81
Z1439	99.81	99.81	99.63
Z1466	99.56	99.88	99.69
Z1534	99.88	99.94	99.94
Z3842	99.56	99.50	99.38
Z3906	99.56	99.75	99.56
Z4242	94.64	94.52	99.63
Z4662	99.50	99.63	99.69
Z4664	99.81	99.81	99.81
Z4665	99.69	99.69	99.56
Z4667	99.69	99.88	99.81
Z4671	99.75	99.88	99.63
Z4672	99.81	99.88	99.75
Z4673	99.50	99.75	99.75
Z4674	99.75	99.56	99.75
Z4675	99.31	99.50	99.69
Z4676	99.88	99.75	99.75
Z4678	99.94	99.81	99.88
Z4681	99.50	99.50	99.69
Z4682	99.88	99.56	99.69
Z4683	99.81	99.75	99.81
Z4684	99.81	99.75	99.81
Z4685	99.63	99.88	99.63
Z4686	98.88	98.94	99.56
Z4687	99.31	99.50	99.38
Z4688	98.94	99.38	99.31
Z4689	99.56	99.63	99.75
Z4690	98.69	98.82	99.75

Z4691	99.13	99.31	99.69
Z4692	99.56	99.56	99.81
Z4693	99.56	99.44	99.88
Z4707	99.31	99.00	99.56
Z4708	99.44	99.56	99.75
Z4709	99.25	99.13	99.69
Z4710	99.31	99.25	99.69
Z4711	99.75	99.69	99.94
Z4756	99.38	99.75	99.63
Z4765	99.56	99.81	99.69
Z5005	99.63	99.50	99.81
Z5035	99.56	99.69	99.75
Z5037	99.25	99.19	99.44
Z5043	99.69	99.63	99.94
Z5826	99.56	99.56	99.88
Z6412	99.75	99.75	99.81
Z6414	99.69	99.56	99.81
Z6415	99.75	99.75	99.75
Z6416	99.63	99.56	99.94
Z6418	99.50	99.44	99.81
Z6419	99.25	99.25	99.63
Z6422	99.75	99.75	99.81
Z6426	99.63	99.56	99.81
Z6427	98.69	98.63	99.63
Z6428	99.00	99.19	99.81
Z6429	99.69	99.75	99.94
Z6430	99.38	99.44	99.69
Z6431	99.81	99.63	99.56
Z6432	99.13	99.13	99.75
Z6433	99.88	99.81	99.94
Z6434	99.88	99.88	100.00
**Average**	99.47	99.49	99.74

2.2.7 Table S10

Table S10: Overview of database standard results of the sequence typing assay for the core validation dataset. The first and second columns list the sample and run name, respectively. The third, fourth, fifth and sixth columns list the number of loci for which the pipeline results were concordant with the reference database information, the number of loci for which the pipeline did not detect any allele but there was one stored in the reference database, the number of loci for which the pipeline detected an allele and for which no allele was stored in the reference database, and the number of loci for which the pipeline detected an allele that was different from the allele stored in the reference database, respectively. See also Figure 3 for a simplified overview.

Sample	Run	True positives	False negatives		
			No allele pipeline	No allele PubMLST	Different allele
Z1001	A	1536	2	51	16
Z1035	A	1528	1	52	24
Z1054	A	1559	7	24	15
Z1073	A	1599	2	3	1
Z1092	A	1546	5	34	20
Z1099	A	1580	3	16	6
Z1269	A	1541	5	33	26
Z1275	A	1572	1	21	11
Z1392	A	1550	3	36	16
Z1439	A	1544	3	47	11
Z1466	A	1565	3	24	13
Z1534	A	1552	8	21	24
Z3842	A	1583	15	2	5
Z3906	A	1576	2	17	10
Z4242	A	1494	77	16	18
Z4662	A	1537	9	38	21
Z4664	A	1553	3	31	18
Z4665	A	1589	2	11	3
Z4667	A	1534	3	50	18
Z4671	A	1568	5	20	12
Z4672	A	1590	2	10	3
Z4673	A	1552	3	34	16
Z4674	A	1558	2	33	12
Z4675	A	1566	5	16	18
Z4676	A	1559	1	30	15
Z4678	A	1559	5	28	13
Z4681	A	1537	3	41	24
Z4682	A	1565	3	23	14
Z4683	A	1566	5	23	11
Z4684	A	1547	3	39	16

Z4685	A	1555	5	30	15
Z4686	A	1533	7	47	18
Z4687	A	1588	8	7	2
Z4688	A	1538	7	38	22
Z4689	A	1552	6	30	17
Z4690	A	1567	17	9	12
Z4691	A	1582	9	9	5
Z4692	A	1540	4	41	20
Z4693	A	1545	2	37	21
Z4707	A	1576	15	7	7
Z4708	A	1530	10	48	17
Z4709	A	1580	15	3	7
Z4710	A	1575	5	13	12
Z4711	A	1498	4	80	23
Z4756	A	1569	3	24	9
Z4765	A	1561	5	21	18
Z5005	A	1558	5	27	15
Z5035	A	1533	10	49	13
Z5037	A	1523	9	52	21
Z5043	A	1552	5	33	15
Z5826	A	1561	5	28	11
Z6412	A	1588	14	1	2
Z6414	A	1538	8	42	17
Z6415	A	1562	3	26	14
Z6416	A	1558	11	23	13
Z6418	A	1548	5	38	14
Z6419	A	1560	7	24	14
Z6422	A	1551	1	30	23
Z6426	A	1561	1	28	15
Z6427	A	1565	5	20	15
Z6428	A	1563	8	20	14
Z6429	A	1582	6	4	13
Z6430	A	1559	7	27	12
Z6431	A	1593	4	5	3
Z6432	A	1549	13	25	18
Z6433	A	1555	2	27	21
Z6434	A	1554	2	31	18
Z1001	B	1537	2	50	16
Z1035	B	1527	2	54	22
Z1054	B	1559	9	21	16
Z1073	B	1596	2	5	2
Z1092	B	1546	3	34	22
Z1099	B	1578	2	19	6
Z1269	B	1546	2	32	25

Z1275	B	1576	1	20	8
Z1392	B	1548	6	36	15
Z1439	B	1541	4	47	13
Z1466	B	1563	7	23	12
Z1534	B	1554	6	21	24
Z3842	B	1580	19	2	4
Z3906	B	1574	3	20	8
Z4242	B	1556	7	20	22
Z4662	B	1541	3	40	21
Z4664	B	1553	2	32	18
Z4665	B	1587	2	11	5
Z4667	B	1535	2	49	19
Z4671	B	1565	7	20	13
Z4672	B	1589	4	10	2
Z4673	B	1551	3	34	17
Z4674	B	1559	0	33	13
Z4675	B	1566	8	17	14
Z4676	B	1560	0	30	15
Z4678	B	1560	4	28	13
Z4681	B	1536	5	40	24
Z4682	B	1567	2	23	13
Z4683	B	1567	3	23	12
Z4684	B	1549	1	39	16
Z4685	B	1553	4	30	18
Z4686	B	1538	1	48	18
Z4687	B	1587	8	7	3
Z4688	B	1540	7	40	18
Z4689	B	1553	4	31	17
Z4690	B	1580	2	10	13
Z4691	B	1586	1	10	8
Z4692	B	1543	1	42	19
Z4693	B	1545	1	39	20
Z4707	B	1580	9	8	8
Z4708	B	1535	7	46	17
Z4709	B	1590	7	2	6
Z4710	B	1579	2	9	15
Z4711	B	1501	2	80	22
Z4756	B	1567	3	24	11
Z4765	B	1557	6	24	18
Z5005	B	1560	3	28	14
Z5035	B	1537	5	49	14
Z5037	B	1528	5	51	21
Z5043	B	1551	4	33	17
Z5826	B	1563	3	28	11

Z6412	B	1587	12	2	4
Z6414	B	1543	3	42	17
Z6415	B	1563	2	26	14
Z6416	B	1564	7	23	11
Z6418	B	1552	0	39	14
Z6419	B	1569	0	22	14
Z6422	B	1552	2	30	21
Z6426	B	1561	0	28	16
Z6427	B	1573	0	19	13
Z6428	B	1571	1	16	17
Z6429	B	1583	4	5	13
Z6430	B	1564	1	28	12
Z6431	B	1592	5	5	3
Z6432	B	1554	5	26	20
Z6433	B	1557	0	27	21
Z6434	B	1556	1	30	18
Z1001	C	1537	2	50	16
Z1035	C	1528	1	54	22
Z1054	C	1561	7	21	16
Z1073	C	1597	0	5	3
Z1092	C	1546	3	34	22
Z1099	C	1578	2	19	6
Z1269	C	1548	1	34	22
Z1275	C	1573	2	20	10
Z1392	C	1545	6	39	15
Z1439	C	1541	6	47	11
Z1466	C	1565	4	23	13
Z1534	C	1553	7	21	24
Z3842	C	1582	16	2	5
Z3906	C	1573	3	20	9
Z4242	C	1558	7	20	20
Z4662	C	1540	5	38	22
Z4664	C	1555	2	31	17
Z4665	C	1591	2	11	1
Z4667	C	1534	2	50	19
Z4671	C	1567	7	20	11
Z4672	C	1590	3	10	2
Z4673	C	1555	1	34	15
Z4674	C	1557	1	34	13
Z4675	C	1567	6	17	15
Z4676	C	1562	0	28	15
Z4678	C	1560	4	28	13
Z4681	C	1538	5	41	21
Z4682	C	1568	4	20	13

Z4683	C	1566	3	23	13
Z4684	C	1549	3	39	14
Z4685	C	1555	5	30	15
Z4686	C	1539	0	47	19
Z4687	C	1589	7	7	2
Z4688	C	1544	2	38	21
Z4689	C	1554	3	31	17
Z4690	C	1582	2	10	11
Z4691	C	1586	2	10	7
Z4692	C	1542	2	42	19
Z4693	C	1547	1	39	18
Z4707	C	1578	9	9	9
Z4708	C	1533	7	48	17
Z4709	C	1590	5	3	7
Z4710	C	1579	3	9	14
Z4711	C	1502	2	80	21
Z4756	C	1569	1	24	11
Z4765	C	1561	4	22	18
Z5005	C	1560	2	28	15
Z5035	C	1538	6	49	12
Z5037	C	1532	2	52	19
Z5043	C	1550	4	34	17
Z5826	C	1563	4	28	10
Z6412	C	1588	12	2	3
Z6414	C	1544	3	41	17
Z6415	C	1565	1	25	14
Z6416	C	1564	6	23	12
Z6418	C	1551	2	39	13
Z6419	C	1569	0	23	13
Z6422	C	1552	0	31	22
Z6426	C	1560	0	28	17
Z6427	C	1570	1	19	15
Z6428	C	1571	2	16	16
Z6429	C	1583	4	5	13
Z6430	C	1568	1	25	11
Z6431	C	1591	3	5	6
Z6432	C	1557	5	26	17
Z6433	C	1557	0	27	21
Z6434	C	1556	1	30	18

2.2.8 Table S11

Table S11: Overview of tool standard results of the sequence typing assay for the core validation dataset. The first and second columns list the sample and run name, respectively. The third, fourth, and fifth columns list the number of concordant loci, the number of multiple perfect hits and the number of other mismatches, respectively. See also Figure 4 for a simplified overview.

Sample	Run	True positives	False negatives	
			Multiple perfect hits	Other mismatches
Z1001	A	1584	20	1
Z1035	A	1589	16	0
Z1054	A	1583	22	0
Z1073	A	1584	21	0
Z1092	A	1588	17	0
Z1099	A	1587	18	0
Z1269	A	1584	21	0
Z1275	A	1587	18	0
Z1392	A	1587	18	0
Z1439	A	1588	17	0
Z1466	A	1588	17	0
Z1534	A	1594	11	0
Z3842	A	1583	22	0
Z3906	A	1584	21	0
Z4242	A	1584	21	0
Z4662	A	1584	21	0
Z4664	A	1582	23	0
Z4665	A	1585	20	0
Z4667	A	1579	26	0
Z4671	A	1584	21	0
Z4672	A	1583	22	0
Z4673	A	1578	27	0
Z4674	A	1580	25	0
Z4675	A	1597	8	0
Z4676	A	1581	24	0
Z4678	A	1584	21	0
Z4681	A	1585	20	0
Z4682	A	1592	13	0
Z4683	A	1579	26	0
Z4684	A	1581	24	0
Z4685	A	1585	20	0
Z4686	A	1578	27	0
Z4687	A	1586	19	0
Z4688	A	1580	25	0

Z4689	A	1588	17	0
Z4690	A	1587	18	0
Z4691	A	1584	21	0
Z4692	A	1580	25	0
Z4693	A	1578	27	0
Z4707	A	1585	20	0
Z4708	A	1587	18	0
Z4709	A	1583	22	0
Z4710	A	1588	17	0
Z4711	A	1584	21	0
Z4756	A	1586	19	0
Z4765	A	1579	26	0
Z5005	A	1588	17	0
Z5035	A	1587	17	1
Z5037	A	1590	15	0
Z5043	A	1585	19	1
Z5826	A	1579	26	0
Z6412	A	1585	20	0
Z6414	A	1587	18	0
Z6415	A	1585	20	0
Z6416	A	1583	22	0
Z6418	A	1582	23	0
Z6419	A	1578	27	0
Z6422	A	1574	31	0
Z6426	A	1575	30	0
Z6427	A	1575	30	0
Z6428	A	1589	16	0
Z6429	A	1588	17	0
Z6430	A	1591	14	0
Z6431	A	1584	21	0
Z6432	A	1579	26	0
Z6433	A	1576	28	1
Z6434	A	1582	23	0
Z1001	B	1587	18	0
Z1035	B	1588	17	0
Z1054	B	1584	21	0
Z1073	B	1583	22	0
Z1092	B	1588	17	0
Z1099	B	1588	17	0
Z1269	B	1585	20	0
Z1275	B	1587	18	0
Z1392	B	1588	16	1
Z1439	B	1588	17	0
Z1466	B	1589	16	0

Z1534	B	1594	11	0
Z3842	B	1582	22	1
Z3906	B	1584	21	0
Z4242	B	1578	24	3
Z4662	B	1584	21	0
Z4664	B	1582	23	0
Z4665	B	1586	19	0
Z4667	B	1578	27	0
Z4671	B	1584	21	0
Z4672	B	1583	22	0
Z4673	B	1578	27	0
Z4674	B	1578	27	0
Z4675	B	1594	11	0
Z4676	B	1579	26	0
Z4678	B	1584	21	0
Z4681	B	1586	19	0
Z4682	B	1590	15	0
Z4683	B	1579	26	0
Z4684	B	1581	24	0
Z4685	B	1585	20	0
Z4686	B	1576	28	1
Z4687	B	1585	20	0
Z4688	B	1579	25	1
Z4689	B	1587	18	0
Z4690	B	1586	19	0
Z4691	B	1582	22	1
Z4692	B	1580	25	0
Z4693	B	1578	27	0
Z4707	B	1583	20	2
Z4708	B	1587	18	0
Z4709	B	1579	26	0
Z4710	B	1587	18	0
Z4711	B	1584	21	0
Z4756	B	1586	19	0
Z4765	B	1579	26	0
Z5005	B	1588	16	1
Z5035	B	1589	16	0
Z5037	B	1590	14	1
Z5043	B	1589	16	0
Z5826	B	1581	24	0
Z6412	B	1585	20	0
Z6414	B	1586	19	0
Z6415	B	1585	20	0
Z6416	B	1585	20	0

Z6418	B	1582	23	0
Z6419	B	1577	28	0
Z6422	B	1576	29	0
Z6426	B	1577	28	0
Z6427	B	1576	29	0
Z6428	B	1588	17	0
Z6429	B	1588	17	0
Z6430	B	1591	14	0
Z6431	B	1584	21	0
Z6432	B	1577	27	1
Z6433	B	1577	27	1
Z6434	B	1584	20	1
Z1001	C	1588	17	0
Z1035	C	1586	18	1
Z1054	C	1585	20	0
Z1073	C	1583	22	0
Z1092	C	1588	17	0
Z1099	C	1587	18	0
Z1269	C	1585	20	0
Z1275	C	1588	17	0
Z1392	C	1587	18	0
Z1439	C	1589	16	0
Z1466	C	1586	19	0
Z1534	C	1595	10	0
Z3842	C	1582	23	0
Z3906	C	1585	20	0
Z4242	C	1577	28	0
Z4662	C	1585	20	0
Z4664	C	1582	23	0
Z4665	C	1585	20	0
Z4667	C	1577	28	0
Z4671	C	1584	21	0
Z4672	C	1583	22	0
Z4673	C	1579	26	0
Z4674	C	1576	29	0
Z4675	C	1594	11	0
Z4676	C	1577	28	0
Z4678	C	1586	19	0
Z4681	C	1584	21	0
Z4682	C	1588	17	0
Z4683	C	1581	24	0
Z4684	C	1582	23	0
Z4685	C	1584	21	0
Z4686	C	1577	28	0

Z4687	C	1585	20	0
Z4688	C	1578	27	0
Z4689	C	1587	18	0
Z4690	C	1587	18	0
Z4691	C	1582	23	0
Z4692	C	1580	25	0
Z4693	C	1578	27	0
Z4707	C	1583	22	0
Z4708	C	1587	18	0
Z4709	C	1580	25	0
Z4710	C	1586	19	0
Z4711	C	1584	21	0
Z4756	C	1583	22	0
Z4765	C	1579	26	0
Z5005	C	1584	21	0
Z5035	C	1590	15	0
Z5037	C	1590	15	0
Z5043	C	1589	16	0
Z5826	C	1581	24	0
Z6412	C	1585	20	0
Z6414	C	1586	19	0
Z6415	C	1586	19	0
Z6416	C	1585	20	0
Z6418	C	1581	24	0
Z6419	C	1577	28	0
Z6422	C	1576	29	0
Z6426	C	1578	27	0
Z6427	C	1577	28	0
Z6428	C	1587	18	0
Z6429	C	1588	17	0
Z6430	C	1592	13	0
Z6431	C	1584	21	0
Z6432	C	1576	29	0
Z6433	C	1575	29	1
Z6434	C	1585	20	0

3.1.1 Table S12

Table S12: Overview of database standard results of the serogroup determination assay for the core validation dataset. The first column lists the sample name, while the second and third columns display the serogroup detected by the pipeline and the serogroup in the reference database (PubMLST), respectively.

Sample	Detected serogroup	PubMLST serogroup
Z1001	A	A
Z1035	A	A
Z1054	A	A
Z1073	A	A
Z1092	A	A
Z1099	A	A
Z1269	A	A
Z1275	A	A
Z1392	A	A
Z1439	A	A
Z1466	A	A
Z1534	A	A
Z3842	B	B
Z3906	A	A
Z4242	B/C	C
Z4662	B	B
Z4664	B	B
Z4665	C	B
Z4667	B	B
Z4671	B	B
Z4672	B	B
Z4673	B	B
Z4674	B	B
Z4675	B	B
Z4676	C	B
Z4678	B	B
Z4681	B	B
Z4682	B	B
Z4683	B	B
Z4684	B	B
Z4685	B	B
Z4686	B	B
Z4687	B	B
Z4688	B	B
Z4689	B	B
Z4690	B	B

Z4691	B	B
Z4692	B	B
Z4693	B	B
Z4707	E	B
Z4708	B	B
Z4709	B	B
Z4710	-	B
Z4711	B	B
Z4756	A	A
Z4765	C	C
Z5005	A	A
Z5035	A	A
Z5037	A	A
Z5043	A	A
Z5826	A	A
Z6412	B	B
Z6414	C	C
Z6415	C	C
Z6416	B	B
Z6418	B	B
Z6419	B	B
Z6422	B	B
Z6426	B	B
Z6427	B	B
Z6428	C	Z
Z6429	-	X
Z6430	X	X
Z6431	Z	Z
Z6432	W	W
Z6433	Y	Y
Z6434	Y	Y

3.1.2 Table S13

Table S13: Overview of tool standard results of the serogroup determination assay for the core validation dataset. The first and second columns list the sample name and the sequencing run, respectively. The next columns list the percentage of loci found for the corresponding serogroup as ndicated in the column title. The penultimate column contains the serogroup as detected by the reference tool when 100% of capsule loci had been detected and '-' otherwise. The last column contains the serogroup detected by the pipeline.

Sample	Run	A	B	C	E	H	L	W	X	Y	Z	Serogroup (PubMLST)	Serogroup (pipeline)
Z1001	A	100%	0%	0%	14%	0%	0%	0%	0%	0%	0%	A	A
Z1035	A	75%	0%	0%	14%	0%	0%	0%	0%	0%	0%	-	A
Z1054	A	100%	0%	0%	14%	0%	0%	0%	0%	0%	0%	A	A
Z1073	A	75%	0%	0%	14%	0%	0%	0%	0%	0%	0%	-	A
Z1092	A	100%	0%	0%	14%	0%	0%	0%	0%	0%	0%	A	A
Z1099	A	75%	0%	0%	14%	0%	0%	0%	0%	0%	0%	-	A
Z1269	A	100%	0%	0%	14%	0%	0%	0%	0%	0%	0%	A	A
Z1275	A	100%	0%	0%	14%	0%	0%	0%	0%	0%	0%	A	A
Z1392	A	100%	0%	0%	14%	0%	0%	0%	0%	0%	0%	A	A
Z1439	A	100%	0%	0%	14%	0%	0%	0%	0%	0%	0%	A	A
Z1466	A	100%	0%	0%	14%	0%	0%	0%	0%	0%	0%	A	A
Z1534	A	100%	0%	0%	14%	0%	0%	0%	0%	0%	0%	A	A
Z3842	A	0%	100%	67%	14%	0%	0%	67%	0%	67%	0%	B	B
Z3906	A	75%	0%	0%	14%	0%	0%	0%	0%	0%	0%	-	A
Z4242	A	0%	40%	33%	14%	0%	0%	33%	0%	33%	0%	-	B
Z4662	A	0%	100%	67%	14%	0%	0%	67%	0%	67%	0%	B	B
Z4664	A	0%	100%	67%	14%	0%	0%	67%	0%	67%	0%	B	B
Z4665	A	0%	80%	100%	14%	0%	0%	67%	0%	67%	0%	C	C
Z4667	A	0%	100%	67%	14%	0%	0%	67%	0%	67%	0%	B	B
Z4671	A	0%	100%	67%	14%	0%	0%	67%	0%	67%	0%	B	B
Z4672	A	0%	100%	67%	14%	0%	0%	67%	0%	67%	0%	B	B
Z4673	A	0%	100%	67%	14%	0%	0%	67%	0%	67%	0%	B	B
Z4674	A	0%	100%	67%	14%	0%	0%	67%	0%	67%	0%	B	B
Z4675	A	0%	100%	67%	14%	0%	0%	67%	0%	67%	0%	B	B
Z4676	A	0%	60%	83%	14%	0%	0%	50%	0%	50%	0%	-	C
Z4678	A	0%	100%	67%	14%	0%	0%	67%	0%	67%	0%	B	B
Z4681	A	0%	100%	67%	14%	0%	0%	67%	0%	67%	0%	B	B
Z4682	A	0%	100%	67%	14%	0%	0%	67%	0%	67%	0%	B	B
Z4683	A	0%	100%	67%	14%	0%	0%	67%	0%	67%	0%	B	B
Z4684	A	0%	100%	67%	14%	0%	0%	67%	0%	67%	0%	B	B
Z4685	A	0%	100%	67%	14%	0%	0%	67%	0%	67%	0%	B	B
Z4686	A	0%	100%	67%	14%	0%	0%	67%	0%	67%	0%	B	B
Z4687	A	0%	100%	67%	14%	0%	0%	67%	0%	67%	0%	B	B
Z4688	A	0%	100%	67%	14%	0%	0%	67%	0%	67%	0%	B	B

Z4689	A	0%	100%	67%	14%	0%	0%	67%	0%	67%	0%	B	B
Z4690	A	0%	80%	67%	14%	0%	0%	67%	0%	67%	0%	-	B
Z4691	A	0%	100%	67%	14%	0%	0%	67%	0%	67%	0%	B	B
Z4692	A	0%	100%	67%	14%	0%	0%	67%	0%	67%	0%	B	B
Z4693	A	0%	100%	67%	14%	0%	0%	67%	0%	67%	0%	B	B
Z4707	A	0%	0%	0%	57%	0%	0%	0%	0%	0%	0%	-	E
Z4708	A	0%	100%	67%	14%	0%	0%	67%	0%	67%	0%	B	B
Z4709	A	0%	100%	67%	14%	0%	0%	67%	0%	67%	0%	B	B
Z4710	A	0%	0%	0%	14%	0%	0%	0%	0%	0%	0%	-	-
Z4711	A	0%	100%	67%	14%	0%	0%	67%	0%	67%	0%	B	B
Z4756	A	100%	0%	0%	14%	0%	0%	0%	0%	0%	0%	A	A
Z4765	A	0%	80%	100%	14%	0%	0%	67%	0%	67%	0%	C	C
Z5005	A	100%	0%	0%	14%	0%	0%	0%	0%	0%	0%	A	A
Z5035	A	100%	0%	0%	14%	0%	0%	0%	0%	0%	0%	A	A
Z5037	A	50%	0%	0%	14%	0%	0%	0%	0%	0%	0%	-	A
Z5043	A	100%	0%	0%	14%	0%	0%	0%	0%	0%	0%	A	A
Z5826	A	100%	0%	0%	14%	0%	0%	0%	0%	0%	0%	A	A
Z6412	A	0%	100%	67%	14%	0%	0%	67%	0%	67%	0%	B	B
Z6414	A	0%	80%	100%	14%	0%	0%	67%	0%	67%	0%	C	C
Z6415	A	0%	80%	100%	14%	0%	0%	67%	0%	67%	0%	C	C
Z6416	A	0%	100%	67%	14%	0%	0%	67%	0%	67%	0%	B	B
Z6418	A	0%	80%	67%	14%	0%	0%	67%	0%	67%	0%	-	B
Z6419	A	0%	100%	67%	14%	0%	0%	67%	0%	67%	0%	B	B
Z6422	A	0%	100%	67%	14%	0%	0%	67%	0%	67%	0%	B	B
Z6426	A	0%	100%	67%	14%	0%	0%	67%	0%	67%	0%	B	B
Z6427	A	0%	100%	67%	14%	0%	0%	67%	0%	67%	0%	B	B
Z6428	A	0%	80%	100%	14%	0%	0%	67%	0%	67%	0%	C	C
Z6429	A	0%	0%	0%	14%	0%	0%	0%	0%	0%	0%	-	-
Z6430	A	0%	0%	0%	14%	0%	0%	0%	67%	0%	0%	-	X
Z6431	A	0%	0%	0%	14%	50%	0%	0%	0%	0%	75%	-	Z
Z6432	A	0%	80%	67%	14%	0%	0%	83%	0%	67%	0%	-	W
Z6433	A	0%	80%	67%	14%	0%	0%	83%	0%	100%	0%	Y	Y
Z6434	A	0%	80%	67%	14%	0%	0%	67%	0%	83%	0%	-	Y
Z1001	B	75%	0%	0%	14%	0%	0%	0%	0%	0%	0%	-	A
Z1035	B	100%	0%	0%	14%	0%	0%	0%	0%	0%	0%	A	A
Z1054	B	50%	0%	0%	14%	0%	0%	0%	0%	0%	0%	-	A
Z1073	B	75%	0%	0%	14%	0%	0%	0%	0%	0%	0%	-	A
Z1092	B	75%	0%	0%	14%	0%	0%	0%	0%	0%	0%	-	A
Z1099	B	100%	0%	0%	14%	0%	0%	0%	0%	0%	0%	A	A
Z1269	B	100%	0%	0%	14%	0%	0%	0%	0%	0%	0%	A	A
Z1275	B	75%	0%	0%	14%	0%	0%	0%	0%	0%	0%	-	A
Z1392	B	75%	0%	0%	14%	0%	0%	0%	0%	0%	0%	-	A

Z1439	B	50%	0%	0%	14%	0%	0%	0%	0%	0%	0%	-	A
Z1466	B	75%	0%	0%	14%	0%	0%	0%	0%	0%	0%	-	A
Z1534	B	75%	0%	0%	14%	0%	0%	0%	0%	0%	0%	-	A
Z3842	B	0%	100%	67%	14%	0%	0%	67%	0%	67%	0%	B	B
Z3906	B	75%	0%	0%	14%	0%	0%	0%	0%	0%	0%	-	A
Z4242	B	0%	80%	100 %	14%	0%	0%	67%	0%	67%	0%	C	C
Z4662	B	0%	100%	67%	14%	0%	0%	67%	0%	67%	0%	B	B
Z4664	B	0%	100%	67%	14%	0%	0%	67%	0%	67%	0%	B	B
Z4665	B	0%	80%	100 %	14%	0%	0%	67%	0%	67%	0%	C	C
Z4667	B	0%	100%	67%	14%	0%	0%	67%	0%	67%	0%	B	B
Z4671	B	0%	100%	67%	14%	0%	0%	67%	0%	67%	0%	B	B
Z4672	B	0%	100%	67%	14%	0%	0%	67%	0%	67%	0%	B	B
Z4673	B	0%	100%	67%	14%	0%	0%	67%	0%	67%	0%	B	B
Z4674	B	0%	100%	67%	14%	0%	0%	67%	0%	67%	0%	B	B
Z4675	B	0%	100%	67%	14%	0%	0%	67%	0%	67%	0%	B	B
Z4676	B	0%	60%	83%	14%	0%	0%	50%	0%	50%	0%	-	C
Z4678	B	0%	100%	67%	14%	0%	0%	67%	0%	67%	0%	B	B
Z4681	B	0%	100%	67%	14%	0%	0%	67%	0%	67%	0%	B	B
Z4682	B	0%	100%	67%	14%	0%	0%	67%	0%	67%	0%	B	B
Z4683	B	0%	100%	67%	14%	0%	0%	67%	0%	67%	0%	B	B
Z4684	B	0%	100%	67%	14%	0%	0%	67%	0%	67%	0%	B	B
Z4685	B	0%	100%	67%	14%	0%	0%	67%	0%	67%	0%	B	B
Z4686	B	0%	100%	67%	14%	0%	0%	67%	0%	67%	0%	B	B
Z4687	B	0%	100%	67%	14%	0%	0%	67%	0%	67%	0%	B	B
Z4688	B	0%	100%	67%	14%	0%	0%	67%	0%	67%	0%	B	B
Z4689	B	0%	100%	67%	14%	0%	0%	67%	0%	67%	0%	B	B
Z4690	B	0%	100%	67%	14%	0%	0%	67%	0%	67%	0%	B	B
Z4691	B	0%	100%	67%	14%	0%	0%	67%	0%	67%	0%	B	B
Z4692	B	0%	100%	67%	14%	0%	0%	67%	0%	67%	0%	B	B
Z4693	B	0%	100%	67%	14%	0%	0%	67%	0%	67%	0%	B	B
Z4707	B	0%	0%	0%	86%	0%	0%	0%	0%	0%	0%	-	E
Z4708	B	0%	100%	67%	14%	0%	0%	67%	0%	67%	0%	B	B
Z4709	B	0%	100%	67%	14%	0%	0%	67%	0%	67%	0%	B	B
Z4710	B	0%	0%	0%	14%	0%	0%	0%	0%	0%	0%	-	-
Z4711	B	0%	100%	67%	14%	0%	0%	67%	0%	67%	0%	B	B
Z4756	B	75%	0%	0%	14%	0%	0%	0%	0%	0%	0%	-	A
Z4765	B	0%	80%	83%	14%	0%	0%	67%	0%	67%	0%	-	C
Z5005	B	50%	0%	0%	14%	0%	0%	0%	0%	0%	0%	-	A
Z5035	B	100%	0%	0%	14%	0%	0%	0%	0%	0%	0%	A	A
Z5037	B	50%	0%	0%	14%	0%	0%	0%	0%	0%	0%	-	A
Z5043	B	100%	0%	0%	14%	0%	0%	0%	0%	0%	0%	A	A
Z5826	B	100%	0%	0%	14%	0%	0%	0%	0%	0%	0%	A	A
Z6412	B	0%	100%	67%	14%	0%	0%	67%	0%	67%	0%	B	B

Z6414	B	0%	80%	100%	14%	0%	0%	67%	0%	67%	0%	C	C
Z6415	B	0%	80%	100%	14%	0%	0%	67%	0%	67%	0%	C	C
Z6416	B	0%	100%	67%	14%	0%	0%	67%	0%	67%	0%	B	B
Z6418	B	0%	80%	67%	14%	0%	0%	67%	0%	67%	0%	-	B
Z6419	B	0%	100%	67%	14%	0%	0%	67%	0%	67%	0%	B	B
Z6422	B	0%	100%	67%	14%	0%	0%	67%	0%	67%	0%	B	B
Z6426	B	0%	100%	67%	14%	0%	0%	67%	0%	67%	0%	B	B
Z6427	B	0%	100%	67%	14%	0%	0%	67%	0%	67%	0%	B	B
Z6428	B	0%	80%	100%	14%	0%	0%	67%	0%	67%	0%	C	C
Z6429	B	0%	0%	0%	14%	0%	0%	0%	0%	0%	0%	-	-
Z6430	B	0%	0%	0%	14%	0%	0%	0%	100%	0%	0%	X	X
Z6431	B	0%	0%	0%	14%	50%	0%	0%	0%	0%	75%	-	Z
Z6432	B	0%	80%	67%	14%	0%	0%	83%	0%	67%	0%	-	W
Z6433	B	0%	80%	67%	14%	0%	0%	83%	0%	100%	0%	Y	Y
Z6434	B	0%	80%	67%	14%	0%	0%	67%	0%	83%	0%	-	Y
Z1001	C	75%	0%	0%	14%	0%	0%	0%	0%	0%	0%	-	A
Z1035	C	100%	0%	0%	14%	0%	0%	0%	0%	0%	0%	A	A
Z1054	C	75%	0%	0%	14%	0%	0%	0%	0%	0%	0%	-	A
Z1073	C	50%	0%	0%	14%	0%	0%	0%	0%	0%	0%	-	A
Z1092	C	75%	0%	0%	14%	0%	0%	0%	0%	0%	0%	-	A
Z1099	C	100%	0%	0%	14%	0%	0%	0%	0%	0%	0%	A	A
Z1269	C	100%	0%	0%	14%	0%	0%	0%	0%	0%	0%	A	A
Z1275	C	100%	0%	0%	14%	0%	0%	0%	0%	0%	0%	A	A
Z1392	C	75%	0%	0%	14%	0%	0%	0%	0%	0%	0%	-	A
Z1439	C	50%	0%	0%	14%	0%	0%	0%	0%	0%	0%	-	A
Z1466	C	75%	0%	0%	14%	0%	0%	0%	0%	0%	0%	-	A
Z1534	C	75%	0%	0%	14%	0%	0%	0%	0%	0%	0%	-	A
Z3842	C	0%	100%	67%	14%	0%	0%	67%	0%	67%	0%	B	B
Z3906	C	75%	0%	0%	14%	0%	0%	0%	0%	0%	0%	-	A
Z4242	C	0%	80%	100%	14%	0%	0%	67%	0%	67%	0%	C	C
Z4662	C	0%	100%	67%	14%	0%	0%	67%	0%	67%	0%	B	B
Z4664	C	0%	100%	67%	14%	0%	0%	67%	0%	67%	0%	B	B
Z4665	C	0%	80%	100%	14%	0%	0%	67%	0%	67%	0%	C	C
Z4667	C	0%	100%	67%	14%	0%	0%	67%	0%	67%	0%	B	B
Z4671	C	0%	100%	67%	14%	0%	0%	67%	0%	67%	0%	B	B
Z4672	C	0%	100%	67%	14%	0%	0%	67%	0%	67%	0%	B	B
Z4673	C	0%	100%	67%	14%	0%	0%	67%	0%	67%	0%	B	B
Z4674	C	0%	100%	67%	14%	0%	0%	67%	0%	67%	0%	B	B
Z4675	C	0%	100%	67%	14%	0%	0%	67%	0%	67%	0%	B	B
Z4676	C	0%	60%	83%	14%	0%	0%	50%	0%	50%	0%	-	C
Z4678	C	0%	100%	67%	14%	0%	0%	67%	0%	67%	0%	B	B

Z4681	C	0%	100%	67%	14%	0%	0%	67%	0%	67%	0%	B	B
Z4682	C	0%	100%	67%	14%	0%	0%	67%	0%	67%	0%	B	B
Z4683	C	0%	100%	67%	14%	0%	0%	67%	0%	67%	0%	B	B
Z4684	C	0%	100%	67%	14%	0%	0%	67%	0%	67%	0%	B	B
Z4685	C	0%	100%	67%	14%	0%	0%	67%	0%	67%	0%	B	B
Z4686	C	0%	100%	67%	14%	0%	0%	67%	0%	67%	0%	B	B
Z4687	C	0%	100%	67%	14%	0%	0%	67%	0%	67%	0%	B	B
Z4688	C	0%	100%	67%	14%	0%	0%	67%	0%	67%	0%	B	B
Z4689	C	0%	100%	67%	14%	0%	0%	67%	0%	67%	0%	B	B
Z4690	C	0%	100%	67%	14%	0%	0%	67%	0%	67%	0%	B	B
Z4691	C	0%	100%	67%	14%	0%	0%	67%	0%	67%	0%	B	B
Z4692	C	0%	100%	67%	14%	0%	0%	67%	0%	67%	0%	B	B
Z4693	C	0%	100%	67%	14%	0%	0%	67%	0%	67%	0%	B	B
Z4707	C	0%	0%	0%	86%	0%	0%	0%	0%	0%	0%	-	E
Z4708	C	0%	100%	67%	14%	0%	0%	67%	0%	67%	0%	B	B
Z4709	C	0%	100%	67%	14%	0%	0%	67%	0%	67%	0%	B	B
Z4710	C	0%	0%	0%	14%	0%	0%	0%	0%	0%	0%	-	-
Z4711	C	0%	100%	67%	14%	0%	0%	67%	0%	67%	0%	B	B
Z4756	C	75%	0%	0%	14%	0%	0%	0%	0%	0%	0%	-	A
Z4765	C	0%	80%	100 %	14%	0%	0%	67%	0%	67%	0%	C	C
Z5005	C	50%	0%	0%	14%	0%	0%	0%	0%	0%	0%	-	A
Z5035	C	75%	0%	0%	14%	0%	0%	0%	0%	0%	0%	-	A
Z5037	C	50%	0%	0%	14%	0%	0%	0%	0%	0%	0%	-	A
Z5043	C	75%	0%	0%	14%	0%	0%	0%	0%	0%	0%	-	A
Z5826	C	100%	0%	0%	14%	0%	0%	0%	0%	0%	0%	A	A
Z6412	C	0%	100%	67%	14%	0%	0%	67%	0%	67%	0%	B	B
Z6414	C	0%	80%	100 %	14%	0%	0%	67%	0%	67%	0%	C	C
Z6415	C	0%	80%	100 %	14%	0%	0%	67%	0%	67%	0%	C	C
Z6416	C	0%	100%	67%	14%	0%	0%	67%	0%	67%	0%	B	B
Z6418	C	0%	80%	67%	14%	0%	0%	67%	0%	67%	0%	-	B
Z6419	C	0%	100%	67%	14%	0%	0%	67%	0%	67%	0%	B	B
Z6422	C	0%	100%	67%	14%	0%	0%	67%	0%	67%	0%	B	B
Z6426	C	0%	100%	67%	14%	0%	0%	67%	0%	67%	0%	B	B
Z6427	C	0%	100%	67%	14%	0%	0%	67%	0%	67%	0%	B	B
Z6428	C	0%	80%	100 %	14%	0%	0%	67%	0%	67%	0%	C	C
Z6429	C	0%	0%	0%	14%	0%	0%	0%	0%	0%	0%	-	-
Z6430	C	0%	0%	0%	14%	0%	0%	0%	100 %	0%	0%	X	X
Z6431	C	0%	0%	0%	14%	50 %	0%	0%	0%	0%	75 %	-	Z
Z6432	C	0%	80%	67%	14%	0%	0%	83%	0%	67%	0%	-	W
Z6433	C	0%	80%	67%	14%	0%	0%	83%	0%	100 %	0%	Y	Y
Z6434	C	0%	80%	67%	14%	0%	0%	67%	0%	83%	0%	-	Y

3.1.3 Table S14

Table S14: Sequence read archive (SRA) accession numbers for all sequenced samples of the core validation dataset. The first column provides the sample identifier (see supplementary Table S4), while the second, third and fourth columns list the accession numbers for runs A, B and C in the NCBI SRA archive, respectively.

Sample	Accession number run A	Accession number run B	Accession number run C
Z1001	SAMN08874818	SAMN08874885	SAMN08874952
Z1035	SAMN08874819	SAMN08874886	SAMN08874953
Z1054	SAMN08874820	SAMN08874887	SAMN08874954
Z1073	SAMN08874821	SAMN08874888	SAMN08874955
Z1092	SAMN08874822	SAMN08874889	SAMN08874956
Z1099	SAMN08874823	SAMN08874890	SAMN08874957
Z1269	SAMN08874824	SAMN08874891	SAMN08874958
Z1275	SAMN08874825	SAMN08874892	SAMN08874959
Z1392	SAMN08874826	SAMN08874893	SAMN08874960
Z1439	SAMN08874827	SAMN08874894	SAMN08874961
Z1466	SAMN08874828	SAMN08874895	SAMN08874962
Z1534	SAMN08874829	SAMN08874896	SAMN08874963
Z3842	SAMN08874830	SAMN08874897	SAMN08874964
Z3906	SAMN08874831	SAMN08874898	SAMN08874965
Z4242	SAMN08874832	SAMN08874899	SAMN08874966
Z4662	SAMN08874833	SAMN08874900	SAMN08874967
Z4664	SAMN08874834	SAMN08874901	SAMN08874968
Z4667	SAMN08874835	SAMN08874902	SAMN08874969
Z4671	SAMN08874836	SAMN08874903	SAMN08874970
Z4672	SAMN08874837	SAMN08874904	SAMN08874971
Z4673	SAMN08874838	SAMN08874905	SAMN08874972
Z4674	SAMN08874839	SAMN08874906	SAMN08874973
Z4675	SAMN08874840	SAMN08874907	SAMN08874974
Z4676	SAMN08874841	SAMN08874908	SAMN08874975
Z4678	SAMN08874842	SAMN08874909	SAMN08874976
Z4681	SAMN08874843	SAMN08874910	SAMN08874977
Z4682	SAMN08874844	SAMN08874911	SAMN08874978
Z4683	SAMN08874845	SAMN08874912	SAMN08874979
Z4684	SAMN08874846	SAMN08874913	SAMN08874980
Z4685	SAMN08874847	SAMN08874914	SAMN08874981
Z4686	SAMN08874848	SAMN08874915	SAMN08874982
Z4688	SAMN08874849	SAMN08874916	SAMN08874983
Z4689	SAMN08874850	SAMN08874917	SAMN08874984
Z4690	SAMN08874851	SAMN08874918	SAMN08874985
Z4692	SAMN08874852	SAMN08874919	SAMN08874986
Z4693	SAMN08874853	SAMN08874920	SAMN08874987
Z4707	SAMN08874854	SAMN08874921	SAMN08874988

Z4708	SAMN08874855	SAMN08874922	SAMN08874989
Z4710	SAMN08874856	SAMN08874923	SAMN08874990
Z4711	SAMN08874857	SAMN08874924	SAMN08874991
Z4756	SAMN08874858	SAMN08874925	SAMN08874992
Z4765	SAMN08874859	SAMN08874926	SAMN08874993
Z5005	SAMN08874860	SAMN08874927	SAMN08874994
Z5035	SAMN08874861	SAMN08874928	SAMN08874995
Z5037	SAMN08874862	SAMN08874929	SAMN08874996
Z5043	SAMN08874863	SAMN08874930	SAMN08874997
Z5826	SAMN08874864	SAMN08874931	SAMN08874998
Z6414	SAMN08874865	SAMN08874932	SAMN08874999
Z6415	SAMN08874866	SAMN08874933	SAMN08875000
Z6416	SAMN08874867	SAMN08874934	SAMN08875001
Z6418	SAMN08874868	SAMN08874935	SAMN08875002
Z6419	SAMN08874869	SAMN08874936	SAMN08875003
Z6422	SAMN08874870	SAMN08874937	SAMN08875004
Z6426	SAMN08874871	SAMN08874938	SAMN08875005
Z6428	SAMN08874872	SAMN08874939	SAMN08875006
Z6429	SAMN08874873	SAMN08874940	SAMN08875007
Z6430	SAMN08874874	SAMN08874941	SAMN08875008
Z6432	SAMN08874875	SAMN08874942	SAMN08875009
Z6433	SAMN08874876	SAMN08874943	SAMN08875010
Z6434	SAMN08874877	SAMN08874944	SAMN08875011
Z6431	SAMN08874878	SAMN08874945	SAMN08875012
Z4665	SAMN08874879	SAMN08874946	SAMN08875013
Z4691	SAMN08874880	SAMN08874947	SAMN08875014
Z4687	SAMN08874881	SAMN08874948	SAMN08875015
Z4709	SAMN08874882	SAMN08874949	SAMN08875016
Z6427	SAMN08874883	SAMN08874950	SAMN08875017
Z6412	SAMN08874884	SAMN08874951	SAMN08875018

3.1.4 Table S15

Table S15: Confusion matrices for the tool standard results of the resistance gene characterization assays for the core validation dataset. The first table contains the definitions of true positives (TP), false positives (FP), false negatives (FN) and true negatives (TN). '+' and '-' refer to the presence and absence of a result. The following tables contain the results for run A, run B, run C, and all runs combined, respectively, for the CARD and ResFinder databases. The reference tools are the RGI online interface for the CARD database, the online ResFinder interface for the ResFinder database and a local installation of NCBI AMRfinder for the NDARO database.

Legend

Actual

		+	-
**Predicted**	+	**TP:** Gene detected by pipeline and reference tool	**FP:** Gene detected by pipeline and not by reference tool
	-	**FN:** Gene detected by reference tool and not by pipeline	**TN:** Gene not detected by pipeline and reference tool

Validation - Run A (CARD)

Actual

		+	-
**Predicted**	+	**TP**<br>469	**FP**<br>67
	-	**FN**<br>0	**TN**<br>52193

Validation - Run B (CARD)

Actual

		+	-
**Predicted**	+	**TP**<br>471	**FP**<br>67
	-	**FN**<br>1	**TN**<br>52190

Validation - Run C (CARD)

Actual

		+	-
**Predi cted**	+	**TP**<br>470	**FP**<br>67

-	**FN** 0	**TN** 52192

Validation - All runs (CARD)

		**Actual** +	-
**Predicted**	+	**TP** 1410	**FP** 201
	-	**FN** 1	**TN** 156575

Validation - Run A (ResFinder)

		**Actual** +	-
**Predicted**	+	**TP** 1	**FP** 0
	-	**FN** 0	**TN** 35643

Validation - Run B (ResFinder)

		**Actual** +	-
**Predicted**	+	**TP** 2	**FP** 0
	-	**FN** 0	**TN** 35642

Validation - Run C (ResFinder)

		**Actual** +	-
**Predicted**	+	**TP** 1	**FP** 0
	-	**FN** 0	**TN** 35643

Validation - All runs (ResFinder)

		**Actual** +	-
**Predi cted**	+	**TP** 4	**FP** 0

-	**FN** 0	**TN** 106928

Validation - Run A (NDARO)

Actual

		+	-
**Predicted**	+	**TP** 1	**FP** 0
	-	**FN** 0	**TN** 60232

Validation - Run B (NDARO)

Actual

		+	-
**Predicted**	+	**TP** 5	**FP** 0
	-	**FN** 0	**TN** 60228

Validation - Run C (NDARO)

Actual

		+	-
**Predicted**	+	**TP** 3	**FP** 0
	-	**FN** 0	**TN** 60230

Validation – All runs (NDARO)

Actual

		+	-
**Predicted**	+	**TP** 9	**FP** 0
	-	**FN** 0	**TN** 180690

3.1.5 Table S16

Table S16: Confusion matrices for the database standard results of the sequence typing assay for the core validation dataset. The first table contains the definitions of true positives (TP), false positives (FP), false negatives (FN) and true negatives (TN). '+' and '-' refer to the presence and absence of a result. The second, third and fourth tables contain the results for run A, run B and run C, respectively. The last table contains the total for all runs combined.

Legend

Predicted \ Actual	+	-
+	**TP:** Detected allele matches reference	**FP:** Allele detected when challenged with *Listeria*
-	**FN:** Detected allele does not match the reference	**TN:** No allele detected when challenged with *Listeria*

Validation - Run A

Predicted \ Actual	+	-
+	**TP** 104307	**FP** 0
-	**FN** 3228	**TN** 117116

Validation - Run B

Predicted \ Actual	+	-
+	**TP** 104480	**FP** 0
-	**FN** 3055	**TN** 117116

Validation - Run C

Predicted \ Actual	+	-
+	**TP** 104523	**FP** 0
-	**FN** 3012	**TN** 117116

Validation - All runs

		Actual +	Actual -
Predicted	+	**TP** 313310	**FP** 0
Predicted	-	**FN** 9295	**TN** 351348

3.1.6 Table S17

Table S17: Confusion matrices for the tool standard results of the sequence typing assay for the core validation dataset. The first table contains the definitions of true positives (TP), false positives (FP), false negatives (FN) and true negatives (TN). '+' and '-' refer to the presence and absence of a result. The second, third and fourth tables contain the results for run A, run B and run C, respectively. The last table contains the total for all runs combined.

Legend

Predicted \ Actual	+	-
+	**TP:** Detected allele matches reference	**FP:** Allele detected when challenged with *Listeria*
-	**FN:** Detected allele does not match the reference	**TN:** No allele detected when challenged with *Listeria*

Validation - Run A

Predicted \ Actual	+	-
+	**TP** 106128	**FP** 0
-	**FN** 1407	**TN** 117116

Validation - Run B

Predicted \ Actual	+	-
+	**TP** 106117	**FP** 0
-	**FN** 1418	**TN** 117116

Validation - Run C

Predicted \ Actual	+	-
+	**TP** 106106	**FP** 0
-	**FN** 1429	**TN** 117116

Validation - All runs

Predicted		Actual: +	Actual: -
	+	**TP** 318351	**FP** 0
	-	**FN** 4254	**TN** 351348

3.1.7 Table S18

Table S18: Confusion matrices for the database standard results of the serogroup determination assay for the core validation dataset. The first table contains the definitions of true positives (TP), false positives (FP), false negatives (FN) and true negatives (TN). '+' and '-' refer to the presence and absence of a result. The second, third and fourth tables contain the results for run A, run B and run C, respectively. The last table contains the total for all runs combined.

Legend

Predicted	Actual	+	-
	+	**TP:** Detected serogroup matches reference	**FP:** Serogroup detected when challenged with *Listeria*
	-	**FN:** Detected serogroup does not match the reference	**TN:** No serogroup detected when challenged with *Listeria*

Validation - Run A

Predicted	Actual	+	-
	+	**TP** 60	**FP** 0
	-	**FN** 7	**TN** 67

Validation - Run B

Predicted	Actual	+	-
	+	**TP** 61	**FP** 0
	-	**FN** 6	**TN** 67

Validation - Run C

Predicted	Actual	+	-
	+	**TP** 61	**FP** 0

-	**FN** 6	**TN** 67

Validation - All runs

Actual

**Predicted**	+	-
+	**TP** 182	**FP** 0
-	**FN** 19	**TN** 201

3.1.8 Table S19

Table S19: Confusion matrices for the tool standard results of the serogroup determination assay for the core validation dataset. The first table contains the definitions of true positives (TP), false positives (FP), false negatives (FN) and true negatives (TN). '+' and '-' refer to the presence and absence of a result. The second, third and fourth tables contain the results for run A, run B and run C, respectively. The last table contains the total for all runs combined.

Legend

Predicted		Actual +	Actual -
	+	**TP:** Detected serogroup matches reference	**FP:** Serogroup detected when challenged with *Listeria*
	-	**FN:** Detected serogroup does not match the reference	**TN:** No serogroup detected when challenged with *Listeria*

Validation - Run A

Predicted		Actual +	Actual -
	+	**TP** 53	**FP** 0
	-	**FN** 0	**TN** 67

Validation - Run B

Predicted		Actual +	Actual -
	+	**TP** 47	**FP** 0
	-	**FN** 0	**TN** 67

Validation - Run C

Predicted		Actual +	Actual -
	+	**TP** 47	**FP** 0

-	**FN**<br>0	**TN**<br>67

Validation - All runs

Actual

		+	-
**Predicted**	+	**TP**<br>147	**FP**<br>0
	-	**FN**<br>0	**TN**<br>201

4 Supporting information: extended validation dataset

4.1 Figures

4.1.1 Figure S7

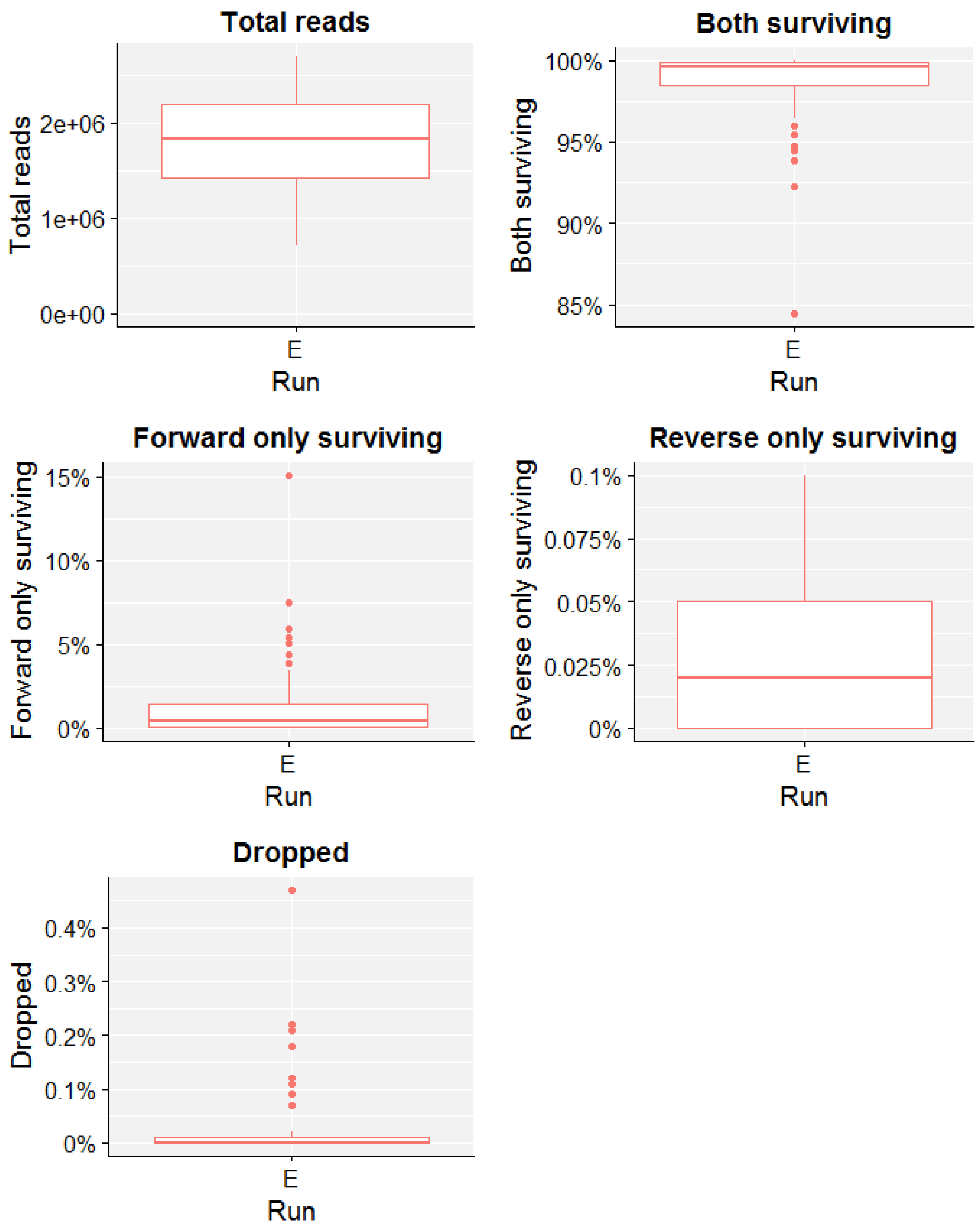

Figure S7: **Overview of read trimming statistics for the extended validation dataset.** Simplified overview of the total number of reads and reads surviving trimming, as denoted by the title above each boxplot, for the extended validation dataset. The ordinate represents the

number of reads (total reads), or the fraction of reads (percentage) that belong to the given category after trimming. Note that the ordinate for the graph depicting ‘Both surviving’ starts at 84% instead of 0% to enable illustrating the variation more clearly. See also supplementary Table S21 for detailed values for all samples.

4.1.2 Figure S8

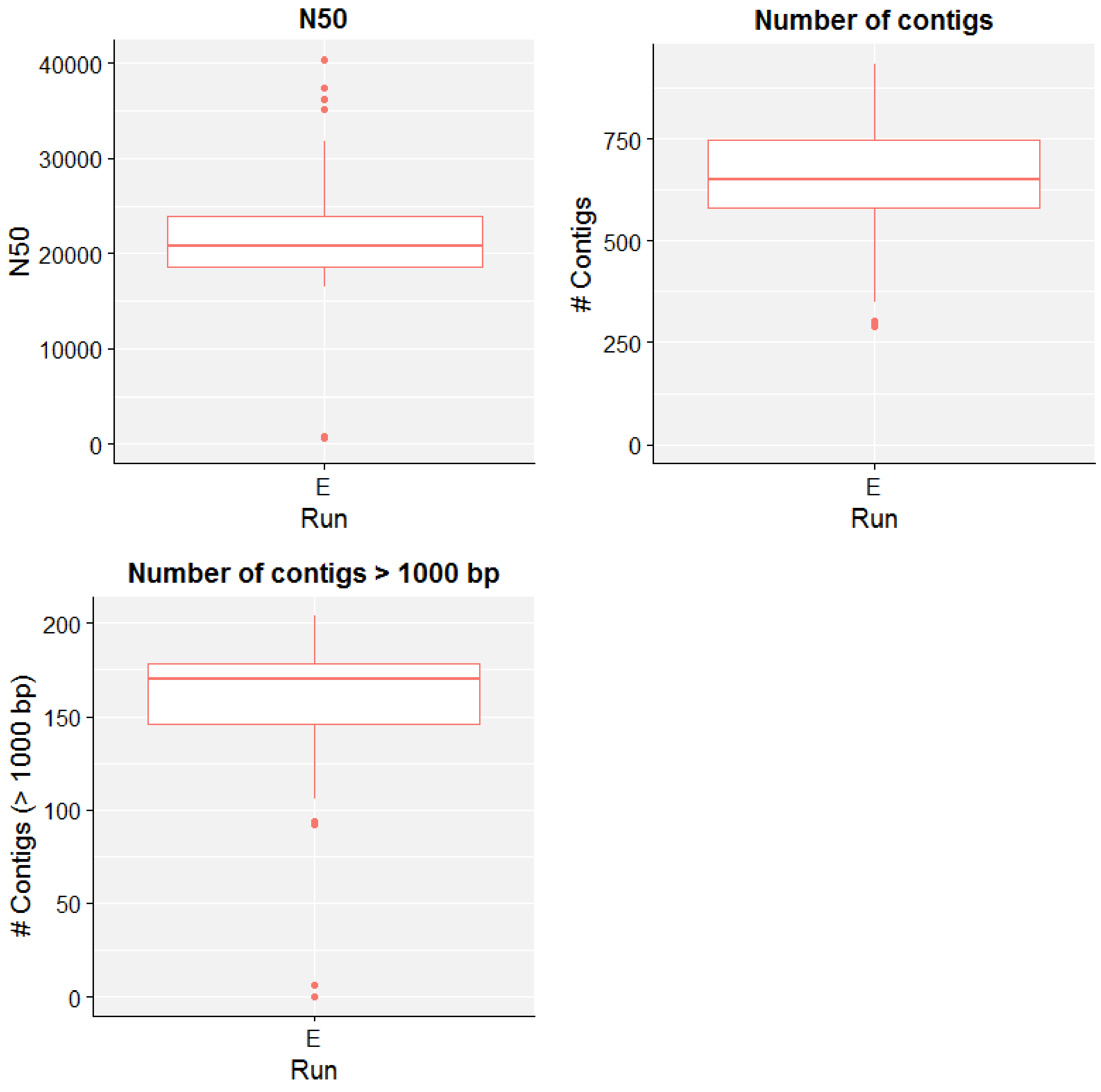

Figure S8: **Overview of assembly statistics for the extended validation dataset.** The abscissa depicts the sequencing run (A, B or C), while the ordinate represents the value for the N50, total number of contigs and number of contigs larger than 1000 bases, as indicated by the title above each boxplot. See also supplementary Table S22 for detailed values for all samples and runs.

4.1.3 Figure S9

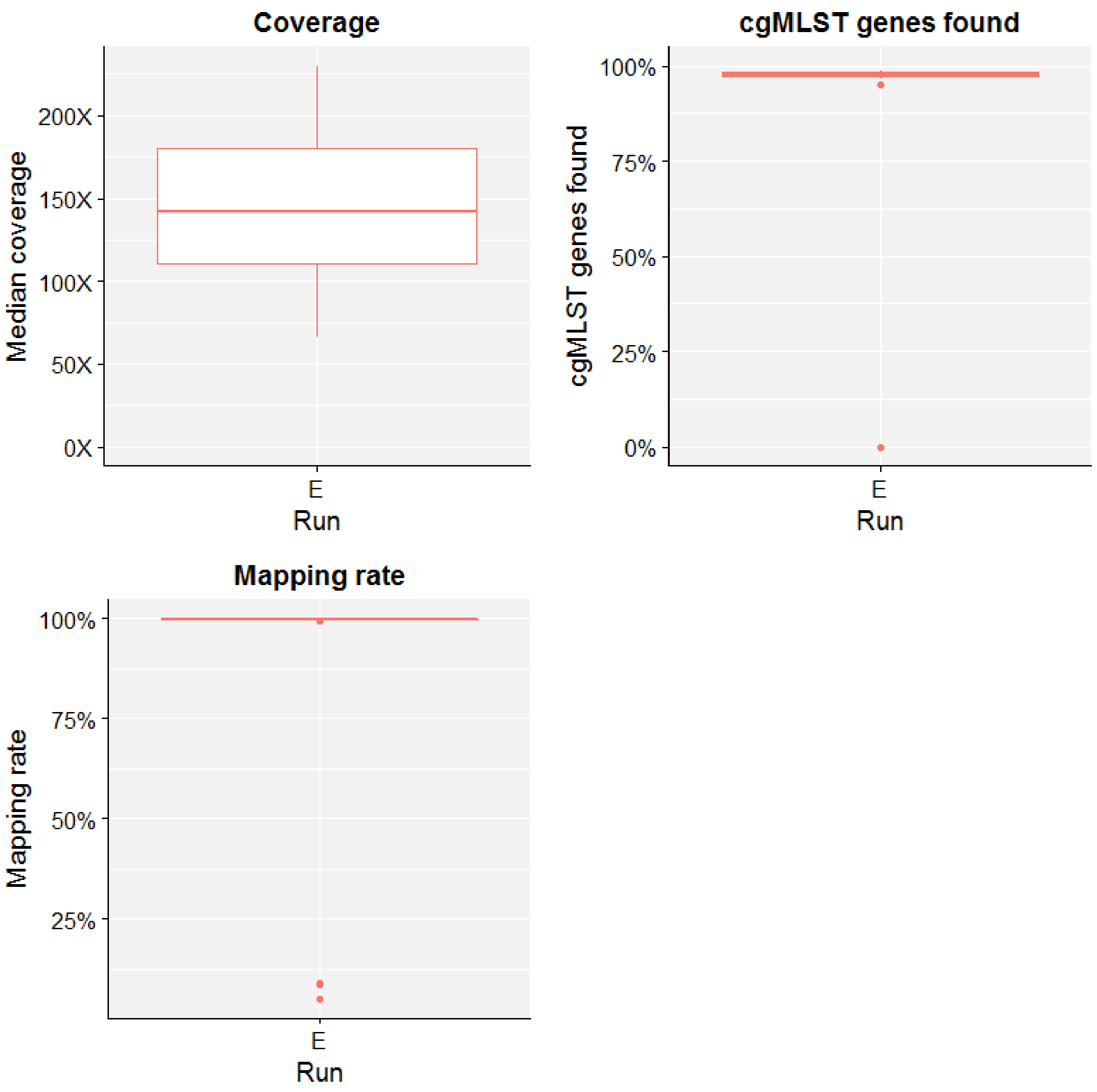

Figure S9: **Overview of advanced quality control statistics for the extended validation dataset**. The ordinate represents the median coverage, percentage of cgMLST genes found and percentage of reads mapping back to the assembly, as indicated by the title above each boxplot. See also supplementary Table S23 for detailed values for all samples and runs.

4.2 Tables

4.2.1 Table S20

Table S20: Overview of samples of the extended validation dataset. The first and second columns list the sample name corresponding to the accession number as listed in the European Nucleotide Archive (ENA) and read length, respectively.

Sample (ENA)	Read length
ERR1134987	100
ERR1161550	100
ERR1292406	50-100
ERR1292463	50-100
ERR1292468	50-100
ERR1292481	50-100
ERR133718	100
ERR1389276	50-100
ERR1389332	50-100
ERR1389340	50-100
ERR1389419	50-100
ERR1389441	50-100
ERR1389456	50-100
ERR1389464	50-100
ERR1554071	50-101
ERR1554129	50-101
ERR1554144	50-101
ERR1717538	50-101
ERR1717540	50-101
ERR1717541	50-101
ERR1717542	50-101
ERR1717544	50-101
ERR1717547	50-101
ERR1717554	50-101
ERR1717556	50-101
ERR1717561	50-101
ERR1717563	50-101
ERR1717566	50-101
ERR1717608	50-101
ERR1717611	50-101
ERR1717612	50-101
ERR1717950	50-100
ERR1717951	50-100
ERR1717952	50-100
ERR1994693	150

ERR1994694	150
ERR1994699	150
ERR2258955	150
ERR2258984	150
ERR2258996	150
ERR278691	100
ERR278692	100
ERR294382	100
ERR294387	100
ERR294391	100
ERR310981	100
ERR310983	100
ERR310986	100
ERR310987	100
ERR310992	100
ERR314115	100
ERR314124	100
ERR314131	100
ERR314148	100
ERR314155	100
ERR314167	100
ERR314197	100
ERR314199	100
ERR314207	100
ERR406036	100
ERR406037	100
ERR406038	100
ERR406575	100
ERR406582	100

4.2.2 Table S21

Table S21: Overview of read trimming statistics for the extended validation dataset. The first and second columns list the sample name and the total number of reads, respectively. The third, fourth, fifth and sixth columns list the fraction of total reads where both members of the read pair passed trimming, the fraction of total reads where only the forward read passed read trimming, the fraction of total reads where only the reverse read passed read trimming, and the fraction of total reads where both read pair members were dropped in the trimming step. See also supplementary Figure S7 for a simplified overview.

Sample	Total reads	Both reads surviving (%)	Forward read only surviving (%)	Reverse read only surviving (%)	Both reads dropped (%)
ERR2258996	1445210	97.51	2.38	0.10	0.01
ERR2258984	1462959	97.81	2.09	0.09	0.01
ERR2258955	1641867	98.06	1.83	0.10	0.01
ERR1994699	702296	98.50	1.43	0.07	0.01
ERR1994694	1018579	98.74	1.17	0.08	0.01
ERR1994693	710864	98.64	1.28	0.07	0.01
ERR1717952	1310983	92.29	7.47	0.02	0.22
ERR1717951	1224121	95.41	4.40	0.02	0.18
ERR1717950	1554131	93.87	5.90	0.02	0.21
ERR1717612	1823691	99.88	0.10	0.02	0.00
ERR1717611	2164663	99.89	0.09	0.02	0.00
ERR1717608	2351660	99.88	0.10	0.02	0.00
ERR1717566	1597199	99.90	0.09	0.02	0.00
ERR1717563	2202006	99.89	0.09	0.02	0.00
ERR1717561	1762066	99.89	0.09	0.02	0.00
ERR1717556	1658252	99.90	0.08	0.02	0.00
ERR1717554	1347885	99.89	0.09	0.02	0.00
ERR1717547	1914156	99.87	0.12	0.01	0.00
ERR1717544	2317864	99.86	0.13	0.01	0.00
ERR1717542	1782567	99.88	0.11	0.02	0.00
ERR1717541	1695627	99.89	0.10	0.01	0.00
ERR1717540	1504053	99.88	0.11	0.02	0.00
ERR1717538	1386879	99.89	0.09	0.02	0.00
ERR1554144	1428463	99.95	0.04	0.01	0.00
ERR1554129	1892369	99.95	0.04	0.01	0.00
ERR1554071	1831289	99.96	0.03	0.01	0.00
ERR1389464	1172896	99.94	0.06	0.00	0.00
ERR1389456	1237114	99.94	0.05	0.00	0.00
ERR1389441	1317254	99.95	0.04	0.00	0.00
ERR1389419	1324923	99.93	0.06	0.00	0.00
ERR1389340	1033646	99.96	0.03	0.00	0.00
ERR1389332	1352509	99.96	0.04	0.00	0.00

ERR1389276	1572670	99.98	0.02	0.00	0.00
ERR1292481	2000230	94.75	5.09	0.05	0.11
ERR1292468	1253882	96.01	3.85	0.04	0.09
ERR1292463	1116628	94.44	5.40	0.04	0.12
ERR1292406	1474880	96.46	3.46	0.01	0.07
ERR1161550	1606485	96.78	3.14	0.01	0.07
ERR1134987	1648860	84.45	15.08	0.01	0.47
ERR406582	1829266	98.53	1.42	0.04	0.01
ERR406575	1339720	97.71	2.23	0.04	0.02
ERR406038	2501544	98.40	1.53	0.06	0.01
ERR406037	2148357	98.38	1.55	0.06	0.01
ERR406036	2474744	98.17	1.76	0.06	0.01
ERR314207	2211596	99.64	0.36	0.00	0.00
ERR314199	2696844	99.58	0.42	0.00	0.00
ERR314197	2507704	99.56	0.43	0.00	0.00
ERR314167	2432881	99.62	0.37	0.00	0.00
ERR314155	2637120	99.64	0.35	0.00	0.00
ERR314148	2554854	99.58	0.41	0.00	0.00
ERR314131	2314911	99.58	0.42	0.00	0.00
ERR314124	2581291	99.61	0.39	0.00	0.00
ERR314115	2186193	99.60	0.40	0.00	0.00
ERR310992	2485538	99.44	0.50	0.06	0.01
ERR310987	2243944	99.48	0.45	0.06	0.00
ERR310986	2230140	99.47	0.47	0.06	0.00
ERR310983	1980273	99.41	0.52	0.06	0.00
ERR310981	2037035	99.47	0.46	0.06	0.00
ERR294391	2065116	99.57	0.43	0.00	0.00
ERR294387	1899285	99.54	0.45	0.00	0.00
ERR294382	2139790	99.55	0.45	0.00	0.00
ERR278692	2187200	99.52	0.42	0.05	0.01
ERR278691	2052419	99.49	0.45	0.05	0.01
ERR133718	2056771	99.58	0.42	0.00	0.00

4.2.3 Table S22

Table S22: Overview of assembly statistics for the extended validation dataset. The first columns list the sample name. The second, third and fourth columns list the N50, number of contigs, and number of contigs > 1,000 bases, respectively. See also supplementary Figure S8 for a simplified overview.

**Sample**	**N50**	**Number of contigs**	**Number of contigs > 1000 bases**
ERR2258996	37341	295	92
ERR2258984	40407	350	106
ERR2258955	31583	356	111
ERR1994699	36161	288	94
ERR1994694	31707	398	122
ERR1994693	35240	305	107
ERR1717952	20796	656	172
ERR1717951	21047	631	166
ERR1717950	21434	577	165
ERR1717612	20923	660	170
ERR1717611	16709	753	199
ERR1717608	18526	901	196
ERR1717566	21046	658	173
ERR1717563	18532	652	182
ERR1717561	18805	613	180
ERR1717556	19676	656	172
ERR1717554	19819	628	168
ERR1717547	20852	643	169
ERR1717544	19985	642	179
ERR1717542	18965	634	179
ERR1717541	18401	653	179
ERR1717540	21047	612	152
ERR1717538	19365	616	175
ERR1554144	20840	796	173
ERR1554129	17811	826	179
ERR1554071	18683	641	180
ERR1389464	18227	727	182
ERR1389456	18863	691	160
ERR1389441	18540	712	173
ERR1389419	18121	718	178
ERR1389340	20009	872	170
ERR1389332	17811	752	179
ERR1389276	16839	913	188
ERR1292481	18552	729	178
ERR1292468	19750	933	196
ERR1292463	17021	780	192

ERR1292406	21019	611	170
ERR1161550	19691	652	172
ERR1134987	16527	896	191
ERR406582	29916	721	113
ERR406575	28879	935	113
ERR406038	28097	496	121
ERR406037	27618	626	123
ERR406036	26446	575	126
ERR314207	23549	487	156
ERR314199	22412	541	170
ERR314197	26765	477	144
ERR314167	20013	911	204
ERR314155	21939	530	171
ERR314148	24181	600	166
ERR314131	691	846	6
ERR314124	20196	774	175
ERR314115	828	842	6
ERR310992	25784	596	146
ERR310987	23848	626	150
ERR310986	22322	660	168
ERR310983	24330	538	145
ERR310981	25028	704	148
ERR294391	21287	743	165
ERR294387	20088	647	171
ERR294382	19843	525	160
ERR278692	22958	582	150
ERR278691	787	860	0
ERR133718	20733	543	171

4.2.4 Table S23

Table S23: Overview of advanced quality control statistics for the extended validation dataset. The first column lists the sample name. The second, third and fourth columns list the percentage of reads mapping back to the assembly, median coverage and number of cgMLST genes detected (on a total of 1605 loci - see Table 2), respectively. See also supplementary Figure S9 for a simplified overview.

Sample	Mapping rate (%)	Median coverage	# of cgMLST loci found
ERR2258996	99.59	166	1551
ERR2258984	99.57	172	1577
ERR2258955	99.55	196	1576
ERR1994699	99.56	83	1587
ERR1994694	99.54	123	1582
ERR1994693	99.62	87	1587
ERR1717952	99.6	96	1582
ERR1717951	99.55	93	1578
ERR1717950	99.64	116	1577
ERR1717612	99.55	147	1577
ERR1717611	99.6	160	1569
ERR1717608	99.41	177	1579
ERR1717566	99.56	130	1580
ERR1717563	99.61	173	1568
ERR1717561	99.62	141	1576
ERR1717556	99.64	134	1581
ERR1717554	99.62	110	1576
ERR1717547	99.63	154	1572
ERR1717544	99.58	185	1581
ERR1717542	99.58	140	1581
ERR1717541	99.6	135	1579
ERR1717540	99.61	125	1582
ERR1717538	99.63	110	1582
ERR1554144	99.54	100	1561
ERR1554129	99.52	150	1559
ERR1554071	99.63	141	1562
ERR1389464	99.59	93	1560
ERR1389456	99.59	102	1569
ERR1389441	99.34	104	1553
ERR1389419	99.63	106	1564
ERR1389340	99.47	82	1559
ERR1389332	99.63	111	1573
ERR1389276	99.53	109	1560
ERR1292481	99.61	143	1570
ERR1292468	99.35	86	1566
ERR1292463	99.57	80	1571

ERR1292406	99.7	113	1566
ERR1161550	99.22	116	1555
ERR1134987	99.29	116	1567
ERR406582	99.44	155	1566
ERR406575	99.24	109	1567
ERR406038	99.64	207	1565
ERR406037	99.53	178	1568
ERR406036	99.57	201	1557
ERR314207	99.64	192	1577
ERR314199	99.65	219	1566
ERR314197	99.65	218	1578
ERR314167	99.47	196	1568
ERR314155	99.65	230	1583
ERR314148	99.6	208	1570
ERR314131	8.98	128	0
ERR314124	99.55	212	1561
ERR314115	8.8	123	0
ERR310992	99.62	214	1578
ERR310987	99.54	194	1577
ERR310986	99.47	182	1573
ERR310983	99.57	171	1579
ERR310981	99.53	175	1582
ERR294391	99.52	180	1564
ERR294387	99.57	167	1569
ERR294382	99.61	185	1556
ERR278692	99.63	191	1556
ERR278691	5.09	66	0
ERR133718	99.62	170	1528

4.2.5 Table S24

Table S24: Detected genes for the resistance characterization assay for the extended validation dataset. The first and second columns list the sample name and database, respectively. The third column contains the name of the gene, while the fourth and fifth columns contain the corresponding percentage sequence identity and percentage of query sequence covered, respectively.

**Sample**	**Database**	**Gene**	**% Identity**	**% Query covered**
ERR1134987	CARD	*farA*	99.01	96.54
ERR1134987	CARD	*farB*	95.68	100
ERR1134987	CARD	*macA*	96.27	100
ERR1134987	CARD	*macB*	97	100
ERR1134987	CARD	*mtrC*	97.34	100
ERR1134987	CARD	*mtrD*	99.16	100
ERR1134987	CARD	*mtrE*	94.38	100
ERR1134987	CARD	*mtrR*	97.63	100
ERR1161550	CARD	*farA*	99.01	96.54
ERR1161550	CARD	*farB*	95.68	100
ERR1161550	CARD	*macA*	96.27	100
ERR1161550	CARD	*macB*	97	100
ERR1161550	CARD	*mtrC*	97.34	100
ERR1161550	CARD	*mtrD*	99.16	100
ERR1161550	CARD	*mtrE*	94.38	100
ERR1161550	CARD	*mtrR*	97.63	100
ERR1292406	CARD	*farA*	99.55	96.54
ERR1292406	CARD	*farB*	97.12	100
ERR1292406	CARD	*macA*	96.1	100
ERR1292406	CARD	*macB*	96.85	100
ERR1292406	CARD	*mtrC*	97.98	100
ERR1292406	CARD	*mtrD*	99.25	100
ERR1292406	CARD	*mtrE*	94.31	100
ERR1292406	CARD	*mtrR*	98.11	100
ERR1292463	CARD	*farA*	99.01	96.54
ERR1292463	CARD	*farB*	95.68	100
ERR1292463	CARD	*macA*	96.27	100
ERR1292463	CARD	*macB*	97	100
ERR1292463	CARD	*mtrC*	97.34	100
ERR1292463	CARD	*mtrD*	99.16	100
ERR1292463	CARD	*mtrE*	94.38	100
ERR1292463	CARD	*mtrR*	97.63	100
ERR1292468	CARD	*farA*	99.01	96.54
ERR1292468	CARD	*farB*	95.68	100
ERR1292468	CARD	*macA*	96.61	100

ERR1292468	CARD	*macB*	97.05	100
ERR1292468	CARD	*mtrC*	97.5	100
ERR1292468	CARD	*mtrD*	99.28	100
ERR1292468	CARD	*mtrE*	94.38	100
ERR1292468	CARD	*mtrR*	97.63	100
ERR1292481	CARD	*farA*	99.01	96.54
ERR1292481	CARD	*farB*	95.68	100
ERR1292481	CARD	*macA*	96.27	100
ERR1292481	CARD	*macB*	97	100
ERR1292481	CARD	*mtrC*	97.34	100
ERR1292481	CARD	*mtrD*	99.16	100
ERR1292481	CARD	*mtrE*	94.38	100
ERR1292481	CARD	*mtrR*	97.63	100
ERR133718	CARD	*farA*	99.1	96.54
ERR133718	CARD	*farB*	97.12	100
ERR133718	CARD	*macA*	96.52	100
ERR133718	CARD	*macB*	97.31	100
ERR133718	CARD	*mtrC*	98.14	100
ERR133718	CARD	*mtrD*	99.09	100
ERR133718	CARD	*mtrE*	93.88	100
ERR133718	CARD	*mtrR*	97.63	100
ERR1389276	CARD	*farA*	99.01	96.54
ERR1389276	CARD	*farB*	95.61	100
ERR1389276	CARD	*macA*	96.27	100
ERR1389276	CARD	*macB*	97	100
ERR1389276	CARD	*mtrC*	97.34	100
ERR1389276	CARD	*mtrD*	99.16	100
ERR1389276	CARD	*mtrE*	94.38	100
ERR1389276	CARD	*mtrR*	97.63	100
ERR1389332	CARD	*farA*	99.01	96.54
ERR1389332	CARD	*farB*	95.68	100
ERR1389332	CARD	*macA*	96.27	100
ERR1389332	CARD	*macB*	97	100
ERR1389332	CARD	*mtrC*	97.34	100
ERR1389332	CARD	*mtrD*	99.16	100
ERR1389332	CARD	*mtrE*	94.38	100
ERR1389332	CARD	*mtrR*	97.63	100
ERR1389340	CARD	*farA*	99.19	96.54
ERR1389340	CARD	*farB*	97.84	100
ERR1389340	CARD	*macA*	96.52	100
ERR1389340	CARD	*macB*	96.95	100
ERR1389340	CARD	*mtrC*	98.14	100
ERR1389340	CARD	*mtrD*	99.16	100
ERR1389340	CARD	*mtrE*	94.1	100

ERR1389340	CARD	*mtrR*	97.48	100
ERR1389419	CARD	*farA*	99.01	96.54
ERR1389419	CARD	*farB*	95.68	100
ERR1389419	CARD	*macA*	96.27	100
ERR1389419	CARD	*macB*	97	100
ERR1389419	CARD	*mtrC*	97.34	100
ERR1389419	CARD	*mtrD*	99.16	100
ERR1389419	CARD	*mtrE*	94.38	100
ERR1389419	CARD	*mtrR*	97.63	100
ERR1389441	CARD	*farA*	99.13	100
ERR1389441	CARD	*farB*	98.69	100
ERR1389441	CARD	*macA*	96.1	100
ERR1389441	CARD	*macB*	96.95	100
ERR1389441	CARD	*mtrC*	97.34	100
ERR1389441	CARD	*mtrD*	99.06	100
ERR1389441	CARD	*mtrE*	94.1	100
ERR1389441	CARD	*mtrR*	97.63	100
ERR1389456	CARD	*farA*	99.01	96.54
ERR1389456	CARD	*farB*	95.68	100
ERR1389456	CARD	*macA*	96.27	100
ERR1389456	CARD	*macB*	97	100
ERR1389456	CARD	*mtrC*	97.18	100
ERR1389456	CARD	*mtrD*	99.13	100
ERR1389456	CARD	*mtrE*	94.45	100
ERR1389456	CARD	*mtrR*	97.63	100
ERR1389464	CARD	*farA*	99.01	96.54
ERR1389464	CARD	*farB*	95.68	100
ERR1389464	CARD	*macA*	96.27	100
ERR1389464	CARD	*macB*	97	100
ERR1389464	CARD	*mtrC*	97.34	100
ERR1389464	CARD	*mtrD*	99.16	100
ERR1389464	CARD	*mtrE*	94.38	100
ERR1389464	CARD	*mtrR*	97.48	100
ERR1554071	CARD	*farA*	99.01	96.54
ERR1554071	CARD	*farB*	95.68	100
ERR1554071	CARD	*macA*	96.27	100
ERR1554071	CARD	*macB*	97	100
ERR1554071	CARD	*mtrC*	97.34	100
ERR1554071	CARD	*mtrD*	99.16	100
ERR1554071	CARD	*mtrE*	94.38	100
ERR1554071	CARD	*mtrR*	97.63	100
ERR1554129	CARD	*farA*	99.01	96.54
ERR1554129	CARD	*farB*	95.68	100
ERR1554129	CARD	*macA*	96.27	100

ERR1554129	CARD	*macB*	97	100
ERR1554129	CARD	*mtrC*	97.34	100
ERR1554129	CARD	*mtrD*	99.16	100
ERR1554129	CARD	*mtrE*	94.38	100
ERR1554129	CARD	*mtrR*	97.63	100
ERR1554144	CARD	*farA*	98.96	100
ERR1554144	CARD	*farB*	96.27	100
ERR1554144	CARD	*macA*	96.01	100
ERR1554144	CARD	*macB*	97.05	100
ERR1554144	CARD	*mtrC*	98.06	100
ERR1554144	CARD	*mtrD*	99.19	100
ERR1554144	CARD	*mtrE*	94.24	100
ERR1554144	CARD	*mtrR*	97.32	100
ERR1717538	CARD	*farA*	99.19	96.54
ERR1717538	CARD	*farB*	97.84	100
ERR1717538	CARD	*macA*	96.35	100
ERR1717538	CARD	*macB*	97.26	100
ERR1717538	CARD	*mtrC*	97.42	100
ERR1717538	CARD	*mtrD*	99.13	100
ERR1717538	CARD	*mtrE*	94.31	100
ERR1717538	CARD	*mtrR*	97.63	100
ERR1717538	ResFinder	*tet(K)*	100	69.93
ERR1717540	CARD	*farA*	99.19	96.54
ERR1717540	CARD	*farB*	97.84	100
ERR1717540	CARD	*macA*	96.35	100
ERR1717540	CARD	*macB*	97.26	100
ERR1717540	CARD	*mtrC*	97.5	100
ERR1717540	CARD	*mtrD*	99.13	100
ERR1717540	CARD	*mtrE*	94.31	100
ERR1717540	CARD	*mtrR*	97.48	100
ERR1717541	CARD	*farA*	99.19	96.54
ERR1717541	CARD	*farB*	97.84	100
ERR1717541	CARD	*macA*	96.35	100
ERR1717541	CARD	*macB*	97.26	100
ERR1717541	CARD	*mtrC*	97.5	100
ERR1717541	CARD	*mtrD*	99.13	100
ERR1717541	CARD	*mtrE*	94.31	100
ERR1717541	CARD	*mtrR*	97.63	100
ERR1717542	CARD	*farA*	99.19	96.54
ERR1717542	CARD	*farB*	97.84	100
ERR1717542	CARD	*macA*	96.35	100
ERR1717542	CARD	*macB*	97.26	100
ERR1717542	CARD	*mtrC*	97.5	100
ERR1717542	CARD	*mtrD*	99.13	100

ERR1717542	CARD	*mtrE*	94.31	100
ERR1717542	CARD	*mtrR*	97.63	100
ERR1717544	CARD	*farA*	99.19	96.54
ERR1717544	CARD	*farB*	97.84	100
ERR1717544	CARD	*macA*	96.35	100
ERR1717544	CARD	*macB*	97.26	100
ERR1717544	CARD	*mtrC*	97.5	100
ERR1717544	CARD	*mtrD*	99.13	100
ERR1717544	CARD	*mtrE*	94.31	100
ERR1717544	CARD	*mtrR*	97.63	100
ERR1717547	CARD	*farA*	99.19	96.54
ERR1717547	CARD	*farB*	97.84	100
ERR1717547	CARD	*macA*	96.35	100
ERR1717547	CARD	*macB*	97.26	100
ERR1717547	CARD	*mtrC*	97.5	100
ERR1717547	CARD	*mtrD*	99.13	100
ERR1717547	CARD	*mtrE*	94.31	100
ERR1717547	CARD	*mtrR*	97.63	100
ERR1717554	CARD	*farA*	99.19	96.54
ERR1717554	CARD	*farB*	97.84	100
ERR1717554	CARD	*macA*	96.35	100
ERR1717554	CARD	*macB*	97.21	100
ERR1717554	CARD	*mtrC*	97.5	100
ERR1717554	CARD	*mtrD*	99.13	100
ERR1717554	CARD	*mtrE*	94.31	100
ERR1717554	CARD	*mtrR*	97.48	100
ERR1717556	CARD	*farA*	99.19	96.54
ERR1717556	CARD	*farB*	97.84	100
ERR1717556	CARD	*macA*	96.35	100
ERR1717556	CARD	*macB*	97.26	100
ERR1717556	CARD	*mtrC*	97.5	100
ERR1717556	CARD	*mtrD*	99.13	100
ERR1717556	CARD	*mtrE*	94.31	100
ERR1717556	CARD	*mtrR*	97.63	100
ERR1717561	CARD	*farA*	99.19	96.54
ERR1717561	CARD	*farB*	97.84	100
ERR1717561	CARD	*macA*	96.35	100
ERR1717561	CARD	*macB*	97.26	100
ERR1717561	CARD	*mtrC*	97.5	100
ERR1717561	CARD	*mtrD*	99.13	100
ERR1717561	CARD	*mtrE*	94.31	100
ERR1717561	CARD	*mtrR*	97.63	100
ERR1717563	CARD	*farA*	99.19	96.54
ERR1717563	CARD	*farB*	97.84	100

ERR1717563	CARD	*macA*	96.35	100
ERR1717563	CARD	*macB*	97.26	100
ERR1717563	CARD	*mtrC*	98.14	100
ERR1717563	CARD	*mtrD*	99.03	100
ERR1717563	CARD	*mtrE*	94.45	100
ERR1717563	CARD	*mtrR*	97.63	100
ERR1717566	CARD	*farA*	99.19	96.54
ERR1717566	CARD	*farB*	97.84	100
ERR1717566	CARD	*macA*	96.35	100
ERR1717566	CARD	*macB*	97.26	100
ERR1717566	CARD	*mtrC*	97.5	100
ERR1717566	CARD	*mtrD*	99.09	100
ERR1717566	CARD	*mtrE*	94.31	100
ERR1717566	CARD	*mtrR*	97.48	100
ERR1717608	CARD	*farA*	99.19	96.54
ERR1717608	CARD	*farB*	97.84	100
ERR1717608	CARD	*macA*	96.35	100
ERR1717608	CARD	*macB*	97.26	100
ERR1717608	CARD	*mtrC*	97.5	100
ERR1717608	CARD	*mtrD*	99.13	100
ERR1717608	CARD	*mtrE*	94.31	100
ERR1717608	CARD	*mtrR*	97.48	100
ERR1717611	CARD	*farA*	99.05	100
ERR1717611	CARD	*farB*	96.27	100
ERR1717611	CARD	*macA*	96.44	100
ERR1717611	CARD	*macB*	96.95	100
ERR1717611	CARD	*mtrC*	96.61	100
ERR1717611	CARD	*mtrD*	99.16	100
ERR1717611	CARD	*mtrE*	94.24	100
ERR1717611	CARD	*mtrR*	97.95	100
ERR1717611	ResFinder	*penA*	92.11	100
ERR1717611	ARG-ANNOT	*penA*	90.25	100
ERR1717612	CARD	*farA*	99.19	96.54
ERR1717612	CARD	*farB*	97.84	100
ERR1717612	CARD	*macA*	96.35	100
ERR1717612	CARD	*macB*	97.54	65.12
ERR1717612	CARD	*mtrC*	97.5	100
ERR1717612	CARD	*mtrD*	99.13	100
ERR1717612	CARD	*mtrE*	94.31	100
ERR1717612	CARD	*mtrR*	97.48	100
ERR1717950	CARD	*farA*	99.19	96.54
ERR1717950	CARD	*farB*	97.84	100
ERR1717950	CARD	*macA*	96.35	100
ERR1717950	CARD	*macB*	97.26	100

ERR1717950	CARD	*mtrC*	97.5	100
ERR1717950	CARD	*mtrD*	99.13	100
ERR1717950	CARD	*mtrE*	94.31	100
ERR1717950	CARD	*mtrR*	97.48	100
ERR1717951	CARD	*farA*	99.19	96.54
ERR1717951	CARD	*farB*	97.84	100
ERR1717951	CARD	*macA*	96.35	100
ERR1717951	CARD	*macB*	97.26	100
ERR1717951	CARD	*mtrC*	97.5	100
ERR1717951	CARD	*mtrD*	99.13	100
ERR1717951	CARD	*mtrE*	94.31	100
ERR1717951	CARD	*mtrR*	97.48	100
ERR1717952	CARD	*farA*	99.19	96.54
ERR1717952	CARD	*farB*	97.84	100
ERR1717952	CARD	*macA*	96.35	100
ERR1717952	CARD	*macB*	97.26	100
ERR1717952	CARD	*mtrC*	97.5	100
ERR1717952	CARD	*mtrD*	99.13	100
ERR1717952	CARD	*mtrE*	94.31	100
ERR1717952	CARD	*mtrR*	97.48	100
ERR1994693	CARD	*farA*	99.19	96.54
ERR1994693	CARD	*farB*	97.84	100
ERR1994693	CARD	*macA*	96.52	100
ERR1994693	CARD	*macB*	97.26	100
ERR1994693	CARD	*mtrC*	97.5	100
ERR1994693	CARD	*mtrD*	99.13	100
ERR1994693	CARD	*mtrE*	94.31	100
ERR1994693	CARD	*mtrR*	97.63	100
ERR1994694	CARD	*farA*	99.19	96.54
ERR1994694	CARD	*farB*	97.84	100
ERR1994694	CARD	*macA*	96.35	100
ERR1994694	CARD	*macB*	97.26	100
ERR1994694	CARD	*mtrC*	97.5	100
ERR1994694	CARD	*mtrD*	99.13	100
ERR1994694	CARD	*mtrE*	94.31	100
ERR1994694	CARD	*mtrR*	97.48	100
ERR1994699	CARD	*farA*	99.19	96.54
ERR1994699	CARD	*farB*	97.84	100
ERR1994699	CARD	*macA*	96.35	100
ERR1994699	CARD	*macB*	97.26	100
ERR1994699	CARD	*mtrC*	97.5	100
ERR1994699	CARD	*mtrD*	99.13	100
ERR1994699	CARD	*mtrE*	94.31	100
ERR1994699	CARD	*mtrR*	97.48	100

ERR2258955	CARD	*farA*	99.01	96.54
ERR2258955	CARD	*farB*	95.68	100
ERR2258955	CARD	*macA*	96.27	100
ERR2258955	CARD	*macB*	97	100
ERR2258955	CARD	*mtrC*	97.34	100
ERR2258955	CARD	*mtrD*	99.16	100
ERR2258955	CARD	*mtrE*	94.38	100
ERR2258955	CARD	*mtrR*	97.63	100
ERR2258984	CARD	*farA*	99.01	96.54
ERR2258984	CARD	*farB*	95.68	100
ERR2258984	CARD	*macA*	96.27	100
ERR2258984	CARD	*macB*	97	100
ERR2258984	CARD	*mtrC*	97.34	100
ERR2258984	CARD	*mtrD*	99.16	100
ERR2258984	CARD	*mtrE*	94.38	100
ERR2258984	CARD	*mtrR*	97.63	100
ERR2258996	CARD	*farA*	99.13	100
ERR2258996	CARD	*farB*	97.97	100
ERR2258996	CARD	*macA*	96.1	100
ERR2258996	CARD	*macB*	96.9	100
ERR2258996	CARD	*mtrC*	98.06	100
ERR2258996	CARD	*mtrD*	99.22	100
ERR2258996	CARD	*mtrE*	94.81	100
ERR2258996	CARD	*mtrR*	97.32	100
ERR2258996	ResFinder	*penA*	92.05	100
ERR2258996	ARG-ANNOT	*penA*	90.19	100
ERR278692	CARD	*farA*	99.13	100
ERR278692	CARD	*farB*	97.97	100
ERR278692	CARD	*macA*	96.1	100
ERR278692	CARD	*macB*	96.9	100
ERR278692	CARD	*mtrC*	98.06	100
ERR278692	CARD	*mtrD*	99.22	100
ERR278692	CARD	*mtrE*	94.81	100
ERR278692	CARD	*mtrR*	97.32	100
ERR278692	ResFinder	*penA*	92.05	100
ERR278692	ARG-ANNOT	*penA*	90.19	100
ERR294382	CARD	*farA*	99.39	100
ERR294382	CARD	*farB*	95.48	100
ERR294382	CARD	*macA*	96.52	100
ERR294382	CARD	*macB*	97	100
ERR294382	CARD	*mtrC*	98.23	100
ERR294382	CARD	*mtrD*	99.06	100
ERR294382	CARD	*mtrE*	93.88	100
ERR294382	CARD	*mtrR*	97.63	100

ERR294387	CARD	*farA*	99.01	96.54
ERR294387	CARD	*farB*	95.68	100
ERR294387	CARD	*macA*	96.27	100
ERR294387	CARD	*macB*	97	100
ERR294387	CARD	*mtrC*	97.34	100
ERR294387	CARD	*mtrD*	99.16	100
ERR294387	CARD	*mtrE*	94.38	100
ERR294387	CARD	*mtrR*	97.63	100
ERR294391	CARD	*farA*	99.01	96.54
ERR294391	CARD	*farB*	95.61	100
ERR294391	CARD	*macA*	96.27	100
ERR294391	CARD	*macB*	97	100
ERR294391	CARD	*mtrC*	97.18	100
ERR294391	CARD	*mtrD*	99.16	100
ERR294391	CARD	*mtrE*	94.31	100
ERR294391	CARD	*mtrR*	97.63	100
ERR310981	CARD	*farA*	99.19	96.54
ERR310981	CARD	*farB*	96.27	100
ERR310981	CARD	*macA*	96.35	100
ERR310981	CARD	*macB*	97.26	100
ERR310981	CARD	*mtrC*	97.5	100
ERR310981	CARD	*mtrD*	99.13	100
ERR310981	CARD	*mtrE*	94.31	100
ERR310981	CARD	*mtrR*	97.63	100
ERR310983	CARD	*farA*	99.19	96.54
ERR310983	CARD	*farB*	97.84	100
ERR310983	CARD	*macA*	96.35	100
ERR310983	CARD	*macB*	97.26	100
ERR310983	CARD	*mtrC*	97.5	100
ERR310983	CARD	*mtrD*	99.13	100
ERR310983	CARD	*mtrE*	94.31	100
ERR310983	CARD	*mtrR*	97.63	100
ERR310986	CARD	*farA*	99.05	100
ERR310986	CARD	*farB*	96.27	100
ERR310986	CARD	*macA*	96.44	100
ERR310986	CARD	*macB*	96.95	100
ERR310986	CARD	*mtrC*	98.06	100
ERR310986	CARD	*mtrD*	99.19	100
ERR310986	CARD	*mtrE*	94.24	100
ERR310986	CARD	*mtrR*	97.48	100
ERR310986	ResFinder	*penA*	92.11	100
ERR310986	ARG-ANNOT	*penA*	90.25	100
ERR310987	CARD	*farA*	99.19	96.54
ERR310987	CARD	*farB*	96.27	100

ERR310987	CARD	*macA*	96.35	100
ERR310987	CARD	*macB*	97.26	100
ERR310987	CARD	*mtrC*	97.5	100
ERR310987	CARD	*mtrD*	99.13	100
ERR310987	CARD	*mtrE*	94.31	100
ERR310987	CARD	*mtrR*	97.63	100
ERR310992	CARD	*farA*	99.19	96.54
ERR310992	CARD	*farB*	97.84	100
ERR310992	CARD	*macA*	96.35	100
ERR310992	CARD	*macB*	97.26	100
ERR310992	CARD	*mtrC*	97.5	100
ERR310992	CARD	*mtrD*	99.13	100
ERR310992	CARD	*mtrE*	94.31	100
ERR310992	CARD	*mtrR*	97.63	100
ERR314124	CARD	*farA*	99.05	100
ERR314124	CARD	*farB*	96.27	100
ERR314124	CARD	*macA*	96.44	100
ERR314124	CARD	*macB*	96.95	100
ERR314124	CARD	*mtrC*	98.06	100
ERR314124	CARD	*mtrD*	99.19	100
ERR314124	CARD	*mtrE*	94.24	100
ERR314124	CARD	*mtrR*	97.48	100
ERR314124	ResFinder	*penA*	92.11	100
ERR314124	ARG-ANNOT	*penA*	90.25	100
ERR314148	CARD	*farA*	99.05	100
ERR314148	CARD	*farB*	96.27	100
ERR314148	CARD	*macA*	96.44	100
ERR314148	CARD	*macB*	96.95	100
ERR314148	CARD	*mtrC*	98.06	100
ERR314148	CARD	*mtrD*	99.19	100
ERR314148	CARD	*mtrE*	94.24	100
ERR314148	CARD	*mtrR*	97.48	100
ERR314148	ResFinder	*penA*	92.11	100
ERR314148	ARG-ANNOT	*penA*	90.25	100
ERR314155	CARD	*farA*	99.19	96.54
ERR314155	CARD	*farB*	97.84	100
ERR314155	CARD	*macA*	96.35	100
ERR314155	CARD	*macB*	97.26	100
ERR314155	CARD	*mtrC*	97.5	100
ERR314155	CARD	*mtrD*	99.13	100
ERR314155	CARD	*mtrE*	94.31	100
ERR314155	CARD	*mtrR*	97.63	100
ERR314167	CARD	*farA*	99.05	100
ERR314167	CARD	*farB*	96.27	100

ERR314167	CARD	*macA*	96.44	100
ERR314167	CARD	*macB*	96.95	100
ERR314167	CARD	*mtrC*	98.06	100
ERR314167	CARD	*mtrD*	99.19	100
ERR314167	CARD	*mtrE*	94.24	100
ERR314167	CARD	*mtrR*	96.37	100
ERR314167	ResFinder	*penA*	92.11	100
ERR314167	ARG-ANNOT	*penA*	90.25	100
ERR314197	CARD	*farA*	99.19	96.54
ERR314197	CARD	*farB*	97.84	100
ERR314197	CARD	*macA*	96.35	100
ERR314197	CARD	*macB*	97.26	100
ERR314197	CARD	*mtrC*	97.5	100
ERR314197	CARD	*mtrD*	99.13	100
ERR314197	CARD	*mtrE*	94.31	100
ERR314197	CARD	*mtrR*	97.63	100
ERR314199	CARD	*farA*	99.05	100
ERR314199	CARD	*farB*	96.27	100
ERR314199	CARD	*macA*	96.44	100
ERR314199	CARD	*macB*	96.95	100
ERR314199	CARD	*mtrC*	98.31	100
ERR314199	CARD	*mtrD*	99.34	100
ERR314199	CARD	*mtrE*	94.24	100
ERR314199	CARD	*mtrR*	97.79	100
ERR314199	ResFinder	*penA*	92.11	100
ERR314199	ARG-ANNOT	*penA*	90.25	100
ERR314207	CARD	*farA*	99.19	96.54
ERR314207	CARD	*farB*	97.84	100
ERR314207	CARD	*macA*	96.35	100
ERR314207	CARD	*macB*	97.26	100
ERR314207	CARD	*mtrC*	97.5	100
ERR314207	CARD	*mtrD*	99.13	100
ERR314207	CARD	*mtrE*	94.31	100
ERR314207	CARD	*mtrR*	97.63	100
ERR406036	CARD	*farA*	99.39	100
ERR406036	CARD	*farB*	95.48	100
ERR406036	CARD	*macA*	96.52	100
ERR406036	CARD	*macB*	97	100
ERR406036	CARD	*mtrC*	98.23	100
ERR406036	CARD	*mtrD*	99.13	100
ERR406036	CARD	*mtrE*	93.88	100
ERR406036	CARD	*mtrR*	97	100
ERR406037	CARD	*farA*	99.01	96.54
ERR406037	CARD	*farB*	95.68	100

ERR406037	CARD	*macA*	96.27	100
ERR406037	CARD	*macB*	97	100
ERR406037	CARD	*mtrC*	97.26	100
ERR406037	CARD	*mtrD*	99.16	100
ERR406037	CARD	*mtrE*	94.38	100
ERR406037	CARD	*mtrR*	97.63	100
ERR406038	CARD	*farA*	99.39	100
ERR406038	CARD	*farB*	95.48	100
ERR406038	CARD	*macA*	96.52	100
ERR406038	CARD	*macB*	97	100
ERR406038	CARD	*mtrC*	98.14	100
ERR406038	CARD	*mtrD*	99.09	100
ERR406038	CARD	*mtrE*	93.88	100
ERR406038	CARD	*mtrR*	97.63	100
ERR406575	CARD	*farA*	98.03	96.54
ERR406575	CARD	*farB*	95.68	100
ERR406575	CARD	*macA*	96.27	100
ERR406575	CARD	*macB*	97	100
ERR406575	CARD	*mtrC*	97.26	100
ERR406575	CARD	*mtrD*	99.19	100
ERR406575	CARD	*mtrE*	94.38	100
ERR406575	CARD	*mtrR*	97.32	100
ERR406582	CARD	*farA*	99.01	96.54
ERR406582	CARD	*farB*	95.68	100
ERR406582	CARD	*macA*	96.27	100
ERR406582	CARD	*macB*	97	100
ERR406582	CARD	*mtrC*	97.34	100
ERR406582	CARD	*mtrD*	99.16	100
ERR406582	CARD	*mtrE*	94.38	100
ERR406582	CARD	*mtrR*	97.63	100

4.2.6 Table S25

Table S25: Overview of tool standard results of the sequence typing assay for the extended validation dataset. The first column lists the sample name. The second, third and fourth columns list the number of concordant loci, the number of multiple perfect hits and the number of other mismatches, respectively. See also Figure 4 for a simplified overview.

Sample	Concordant	Multiple perfect hits	Other mismatches
ERR2258996	1597	8	0
ERR2258984	1597	8	0
ERR2258955	1595	10	0
ERR1994699	1596	9	0
ERR1994694	1597	8	0
ERR1994693	1598	7	0
ERR1717952	1597	8	0
ERR1717951	1597	8	0
ERR1717950	1597	8	0
ERR1717612	1598	7	0
ERR1717611	1596	9	0
ERR1717608	1598	7	0
ERR1717566	1597	8	0
ERR1717563	1598	7	0
ERR1717561	1599	6	0
ERR1717556	1597	8	0
ERR1717554	1598	7	0
ERR1717547	1597	8	0
ERR1717544	1597	8	0
ERR1717542	1597	8	0
ERR1717541	1598	7	0
ERR1717540	1596	9	0
ERR1717538	1597	8	0
ERR1554144	1596	9	0
ERR1554129	1596	9	0
ERR1554071	1598	7	0
ERR1389464	1601	4	0
ERR1389456	1598	7	0
ERR1389441	1596	9	0
ERR1389419	1599	6	0
ERR1389340	1596	9	0
ERR1389332	1594	11	0
ERR1389276	1593	12	0
ERR1292481	1598	7	0
ERR1292468	1597	8	0
ERR1292463	1599	6	0

ERR1292406	1598	7	0
ERR1161550	1600	5	0
ERR1134987	1599	6	0
ERR406582	1593	12	0
ERR406575	1595	10	0
ERR406038	1597	8	0
ERR406037	1595	10	0
ERR406036	1599	6	0
ERR314207	1598	7	0
ERR314199	1596	9	0
ERR314197	1597	8	0
ERR314167	1597	8	0
ERR314155	1598	7	0
ERR314148	1597	8	0
ERR314131	1605	0	0
ERR314124	1598	7	0
ERR314115	1605	0	0
ERR310992	1598	7	0
ERR310987	1597	8	0
ERR310986	1597	8	0
ERR310983	1598	7	0
ERR310981	1596	9	0
ERR294391	1594	11	0
ERR294387	1595	10	0
ERR294382	1599	6	0
ERR278692	1594	11	0
ERR278691	1605	0	0
ERR133718	1597	8	0

4.2.7 Table S26

Table S26: Overview of database standard results of the serogroup determination assay for the extended validation dataset. The first column lists the sample name, while the second and third columns display the serogroup detected by the pipeline and the serogroup in the reference PubMLST database, respectively.

**Sample**	**Detected serogroup**	**Expected serogroup**
ERR2258996	Y	Y
ERR2258984	Y	Y
ERR2258955	Y	Y
ERR1994699	W	W
ERR1994694	W	W
ERR1994693	W	W
ERR1717952	W	W
ERR1717951	W	W
ERR1717950	W	W
ERR1717612	W	W
ERR1717611	W	W
ERR1717608	W	W
ERR1717566	W	W
ERR1717563	W	W
ERR1717561	W	W
ERR1717556	W	W
ERR1717554	W	W
ERR1717547	W	W
ERR1717544	W	W
ERR1717542	W	W
ERR1717541	W	W
ERR1717540	W	W
ERR1717538	W	W
ERR1554144	Y	Y
ERR1554129	Y	Y
ERR1554071	Y	Y
ERR1389464	Y	Y
ERR1389456	Y	Y
ERR1389441	Y	Y
ERR1389419	Y	Y
ERR1389340	W	Y
ERR1389332	Y	Y
ERR1389276	Y	Y
ERR1292481	Y	Y
ERR1292468	Y	Y
ERR1292463	Y	Y

ERR1292406	Y	Y
ERR1161550	Y	Y
ERR1134987	Y	Y
ERR406582	Y	Y
ERR406575	Y	Y
ERR406038	Y	Y
ERR406037	B	Y
ERR406036	Y	Y
ERR314207	W	W
ERR314199	W	W
ERR314197	W	W
ERR314167	W	W
ERR314155	W	W
ERR314148	W	W
ERR314131	A	W
ERR314124	W	W
ERR314115	A	W
ERR310992	W	W
ERR310987	W	W
ERR310986	W	W
ERR310983	W	W
ERR310981	W	W
ERR294391	Y	Y
ERR294387	Y	Y
ERR294382	Y	Y
ERR278692	Y	Y
ERR278691	A	Y
ERR133718	Y	Y

4.2.8 Table S27

Table S27: Overview of tool standard results of the serogroup determination assay for the extended validation dataset. The first column lists the sample name. The next columns list the percentage of loci found for the corresponding serogroup as indicated in the column title. The penultimate column contains the serogroup as detected by the reference tool when 100% of capsule loci had been detected and '-' otherwise. The last column contains the serogroup detected by the pipeline

Sample	A	B	C	E	H	L	W	X	Y	Z	Serogroup (PubMLST)	Serogroup (Pipeline)
ERR1134987	0%	80%	67%	0%	0%	0%	83%	0%	100%	0%	Y	Y
ERR1161550	0%	80%	67%	0%	0%	0%	83%	0%	100%	0%	Y	Y
ERR1292406	0%	80%	67%	0%	0%	0%	83%	0%	100%	0%	Y	Y
ERR1292463	0%	80%	67%	0%	0%	0%	83%	0%	100%	0%	Y	Y
ERR1292468	0%	80%	67%	0%	0%	0%	83%	0%	100%	0%	Y	Y
ERR1292481	0%	80%	67%	0%	0%	0%	83%	0%	100%	0%	Y	Y
ERR133718	0%	80%	67%	0%	0%	0%	83%	0%	100%	0%	Y	Y
ERR1389276	0%	80%	67%	0%	0%	0%	83%	0%	100%	0%	Y	Y
ERR1389332	0%	80%	67%	0%	0%	0%	83%	0%	100%	0%	Y	Y
ERR1389340	0%	80%	67%	0%	0%	0%	83%	0%	83%	0%	-	W
ERR1389419	0%	80%	67%	0%	0%	0%	83%	0%	100%	0%	Y	Y
ERR1389441	0%	80%	67%	0%	0%	0%	83%	0%	100%	0%	Y	Y
ERR1389456	0%	80%	67%	0%	0%	0%	83%	0%	100%	0%	Y	Y
ERR1389464	0%	80%	67%	0%	0%	0%	67%	0%	83%	0%	-	Y
ERR1554071	0%	80%	67%	0%	0%	0%	83%	0%	100%	0%	Y	Y
ERR1554129	0%	80%	67%	0%	0%	0%	83%	0%	100%	0%	Y	Y
ERR1554144	0%	80%	67%	0%	0%	0%	67%	0%	83%	0%	-	Y
ERR1717538	0%	80%	67%	0%	0%	0%	83%	0%	67%	0%	-	W
ERR1717540	0%	80%	67%	0%	0%	0%	83%	0%	67%	0%	-	W
ERR1717541	0%	80%	67%	0%	0%	0%	83%	0%	67%	0%	-	W
ERR1717542	0%	80%	67%	0%	0%	0%	83%	0%	67%	0%	-	W
ERR1717544	0%	80%	67%	0%	0%	0%	83%	0%	67%	0%	-	W
ERR1717547	0%	80%	67%	0%	0%	0%	83%	0%	67%	0%	-	W
ERR1717554	0%	80%	67%	0%	0%	0%	83%	0%	67%	0%	-	W
ERR1717556	0%	80%	67%	0%	0%	0%	83%	0%	67%	0%	-	W
ERR1717561	0%	80%	67%	0%	0%	0%	83%	0%	67%	0%	-	W
ERR1717563	0%	80%	67%	0%	0%	0%	83%	0%	67%	0%	-	W
ERR1717566	0%	80%	67%	0%	0%	0%	83%	0%	67%	0%	-	W
ERR1717608	0%	80%	67%	0%	0%	0%	83%	0%	67%	0%	-	W
ERR1717611	0%	80%	67%	0%	0%	0%	83%	0%	67%	0%	-	W
ERR1717612	0%	80%	67%	0%	0%	0%	83%	0%	67%	0%	-	W
ERR1717950	0%	80%	67%	0%	0%	0%	83%	0%	67%	0%	-	W
ERR1717951	0%	80%	67%	0%	0%	0%	83%	0%	67%	0%	-	W
ERR1717952	0%	80%	67%	0%	0%	0%	83%	0%	67%	0%	-	W

ERR1994693	0%	80%	67%	0%	0%	0%	83%	0%	67%	0%	-	W
ERR1994694	0%	80%	67%	0%	0%	0%	83%	0%	67%	0%	-	W
ERR1994699	0%	80%	67%	0%	0%	0%	83%	0%	67%	0%	-	W
ERR2258955	0%	80%	67%	0%	0%	0%	83%	0%	100%	0%	Y	Y
ERR2258984	0%	80%	67%	0%	0%	0%	83%	0%	100%	0%	Y	Y
ERR2258996	0%	60%	50%	0%	0%	0%	67%	0%	83%	0%	-	Y
ERR278691	0%	0%	0%	0%	0%	0%	0%	0%	0%	0%	-	A
ERR278692	0%	80%	67%	0%	0%	0%	83%	0%	100%	0%	Y	Y
ERR294382	0%	80%	67%	0%	0%	0%	83%	0%	100%	0%	Y	Y
ERR294387	0%	80%	67%	0%	0%	0%	83%	0%	100%	0%	Y	Y
ERR294391	0%	80%	67%	0%	0%	0%	83%	0%	100%	0%	Y	Y
ERR310981	0%	80%	67%	0%	0%	0%	83%	0%	67%	0%	-	W
ERR310983	0%	80%	67%	0%	0%	0%	83%	0%	67%	0%	-	W
ERR310986	0%	80%	67%	0%	0%	0%	83%	0%	67%	0%	-	W
ERR310987	0%	80%	67%	0%	0%	0%	83%	0%	67%	0%	-	W
ERR310992	0%	80%	67%	0%	0%	0%	83%	0%	67%	0%	-	W
ERR314115	0%	0%	0%	0%	0%	0%	0%	0%	0%	0%	-	A
ERR314124	0%	80%	67%	0%	0%	0%	83%	0%	67%	0%	-	W
ERR314131	0%	0%	0%	0%	0%	0%	0%	0%	0%	0%	-	A
ERR314148	0%	80%	67%	0%	0%	0%	83%	0%	67%	0%	-	W
ERR314155	0%	80%	67%	0%	0%	0%	83%	0%	67%	0%	-	W
ERR314167	0%	80%	67%	0%	0%	0%	83%	0%	67%	0%	-	W
ERR314197	0%	80%	67%	0%	0%	0%	83%	0%	67%	0%	-	W
ERR314199	0%	80%	67%	0%	0%	0%	83%	0%	67%	0%	-	W
ERR314207	0%	80%	67%	0%	0%	0%	83%	0%	67%	0%	-	W
ERR406036	0%	80%	67%	0%	0%	0%	83%	0%	100%	0%	Y	Y
ERR406037	0%	80%	67%	0%	0%	0%	67%	0%	67%	0%	-	B
ERR406038	0%	80%	67%	0%	0%	0%	83%	0%	100%	0%	Y	Y
ERR406575	0%	80%	67%	0%	0%	0%	83%	0%	100%	0%	Y	Y
ERR406582	0%	80%	67%	0%	0%	0%	83%	0%	100%	0%	Y	Y

4.2.9 Table S28

Table S28: Confusion matrices for the tool standard results of the resistance gene detection assays for the extended validation dataset. The first table contains the definitions of true positives (TP), false positives (FP), false negatives (FN) and true negatives (TN). '+' and '-' refer to the presence and absence of a result. The following tables contain the results for the CARD and ResFinder databases. The reference tools are RGI and the online ResFinder interface for the CARD and ResFinder databases, respectively.

Legend

		Actual +	Actual -
**Predicted**	+	**TP:** Gene detected by pipeline and reference tool	**FP:** Gene detected by pipeline and not by reference tool
	-	**FN:** Gene detected by reference tool and not by pipeline	**TN:** Gene not detected by pipeline and reference tool

Extended - CARD

		Actual +	Actual -
**Predicted**	+	**TP** 427	**FP** 61
	-	**FN** 0	**TN** 49880

Extended - ResFinder

		Actual +	Actual -
**Predicted**	+	**TP** 9	**FP** 0
	-	**FN** 0	**TN** 35959

Extended - NDARO

		Actual +	Actual -
**Predicted**	+	**TP** 0	**FP** 0

-	**FN**<br>0	**TN**<br>57536

4.2.10 Table S29

Table S29: Confusion matrices for the tool standard results of the sequence typing assay for the extended validation dataset. The first table contains the definitions of true positives (TP), false positives (FP), false negatives (FN) and true negatives (TN). '+' and '-' refer to the presence and absence of a result. The second table contains the corresponding values.

Legend

		Actual: +	Actual: -
**Predicted**	+	**TP:** Detected allele matches reference	**FP:** Allele detected when challenged with *Listeria*
**Predicted**	-	**FN:** Detected allele does not match the reference	**TN:** No allele detected when challenged with *Listeria*

Results

		Actual: +	Actual: -
**Predicted**	+	**TP** 102232	**FP** 0
**Predicted**	-	**FN** 488	**TN** 111872

4.2.11 Table S30

Table S30: Confusion matrices for the database standard results of the serogroup determination assay for the extended validation dataset. The first table contains the definitions of true positives (TP), false positives (FP), false negatives (FN) and true negatives (TN). '+' and '-' refer to the presence and absence of a result. The second table contains the corresponding values.

Legend

Actual

Predicted		+	-
	+	**TP:** Detected serogroup matches reference	**FP:** Serogroup detected when challenged with *Listeria*
	-	**FN:** Detected serogroup does not match the reference	**TN:** No serogroup detected when challenged with *Listeria*

Results

Actual

Predicted		+	-
	+	**TP** 59	**FP** 0
	-	**FN** 5	**TN** 64

4.2.12 Table S31

Table S31: Confusion matrices for the tool standard results of the serogroup determination assay for the extended validation dataset. The first table contains the definitions of true positives (TP), false positives (FP), false negatives (FN) and true negatives (TN). '+' and '-' refer to the presence and absence of a result. The second table contains the corresponding values.

Legend

Actual

Predicted		+	-
	+	**TP:** Detected serogroup matches reference	**FP:** Serogroup detected when challenged with *Listeria*

-	**FN:** Detected serogroup does not match the reference	**TN:** No serogroup detected when challenged with *Listeria*

Results

Predicted		Actual +	Actual -
	+	**TP** 24	**FP** 0
	-	**FN** 0	**TN** 64

5 References

Bratcher, H. B., Corton, C., Jolley, K. A., Parkhill, J., and Maiden, M. C. (2014). A gene-by-gene population genomics platform: de novo assembly, annotation and genealogical analysis of 108 representative Neisseria meningitidis genomes. *BMC Genomics* 15, 1138. doi:10.1186/1471-2164-15-1138.

Jolley, K. A., and Maiden, M. C. J. (2010). BIGSdb: Scalable analysis of bacterial genome variation at the population level. *BMC Bioinformatics* 11, 595. doi:10.1186/1471-2105-11-595.

O'Leary, N. A., Wright, M. W., Brister, J. R., Ciufo, S., Haddad, D., McVeigh, R., et al. (2016). Reference sequence (RefSeq) database at NCBI: Current status, taxonomic expansion, and functional annotation. *Nucleic Acids Res.* 44, D733–D745. doi:10.1093/nar/gkv1189.

Ondov, B. D., Bergman, N. H., and Phillippy, A. M. (2011). Interactive metagenomic visualization in a Web browser. *BMC Bioinformatics* 12. doi:10.1186/1471-2105-12-385.

Wood, D. E., and Salzberg, S. L. (2014). Kraken: ultrafast metagenomic sequence classification using exact alignments. *Genome Biol.* 15, R46.
